# Influence of Non-Biodegradable ZrO_2_ and Y_2_O_3_ Nanoparticles on the Genomic and Redox Status of Chicken Embryo

**DOI:** 10.3390/nano16211347

**Published:** 2026-10-23

**Authors:** Bartłomiej Dominiak, Julita Rosowska, Agnieszka Wal, Alicja Majewska, Lena M. Rudy, Bartłomiej S. Witkowski, Łukasz Wachnicki, Jarosław Kaszewski, Anna Słońska, Joanna Cymerys, Mikołaj A. Gralak, Marek Godlewski, Michał M. Godlewski

**Affiliations:** 1 Department of Physiological Sciences, Institute of Veterinary Medicine, Warsaw University of Life Sciences—SGGW, Nowoursynowska 159, 02-776 Warsaw, Poland; julita_rosowska@sggw.edu.pl (J.R.); alicja_majewska@sggw.edu.pl (A.M.); mikolaj_gralak@sggw.edu.pl (M.A.G.); michal_godlewski@sggw.edu.pl (M.M.G.); 2 Institute of Physics, Polish Academy of Sciences, Al. Lotników 32/46, 02-668 Warsaw, Poland; bwitkow@ifpan.edu.pl (B.S.W.); lwachn@ifpan.edu.pl (Ł.W.); kaszewski@ifpan.edu.pl (J.K.); godlew@ifpan.edu.pl (M.G.); 3 Department of Botany and Plant Physiology, Institute of Biology, Warsaw University of Life Sciences—SGGW, 02-776 Warsaw, Poland; agnieszka_wal@sggw.edu.pl; 4 Scientific Society of Veterinary Medicine Students, Warsaw University of Life Sciences—SGGW, Nowoursynowska 159, 02-776 Warsaw, Poland; s222313@sggw.edu.pl; 5 Division of Microbiology, Department of Preclinical Sciences, Institute of Veterinary Medicine, Warsaw University of Life Sciences—SGGW, Ciszewskiego 8, 02-786 Warsaw, Poland; anna_slonska-zielonka@sggw.edu.pl (A.S.); joanna_cymerys@sggw.edu.pl (J.C.)

**Keywords:** ZrO_2_ nanoparticles, Y_2_O_3_ nanoparticles, shape of the nanoparticles, embryotoxicity, full genomic microarrays, oxidative stress, chicken embryo model

## Abstract

With the growing use of nanotechnology, the issue of exposure to non-biodegradable nanomaterials is becoming increasingly significant, raising concerns about their potential impact on developing organisms. Their use in dentistry, optoelectronics and biomedicine leads to environmental exposure. Zirconium oxide (ZrO_2_) and yttrium oxide (Y_2_O_3_) nanoparticles are chemically stable and poorly soluble materials; however, their embryotoxicity remains extremely poorly characterised. This study assessed the effect of ZrO_2_ and Y_2_O_3_ nanoparticles on the development of chicken embryos. The nanoparticles were administered into the air chamber of the egg on the first day of incubation at concentrations of 10 and 100 µg/mL. The redox status was assessed based on superoxide dismutase activity, malondialdehyde content and the level of carbonylated proteins. Transcriptomic responses were evaluated using a whole-genome microarray, and selected genes were verified by qPCR. Both types of nanoparticles induced time-dependent changes in markers of oxidative stress. Transcriptomic analysis revealed distinctly different responses, with seven genes showing altered expression following ZrO_2_ administration, compared with 555 following Y_2_O_3_ administration. These results indicate that non-biodegradable metal oxide nanoparticles can influence the redox balance and gene expression during early embryonic development, with the extent of the response depending on the type of nanoparticle.

## 1. Introduction

The increasing use of ZrO_2_ and Y_2_O_3_ nanoparticles in technological and biomedical applications creates risks of exposure to humans and the environment. ZrO_2_ nanoparticles are of particular importance in dentistry, where they are used or being investigated in dental ceramics, implants, coatings and composite materials due to their mechanical properties, chemical stability and biocompatibility [1]. Y_2_O_3_ nanoparticles also have a broad range of applications in technology and biomedicine. Due to their physicochemical and optical properties, they are used or studied as matrix materials for rare-earth element dopants, phosphors and laser materials, as well as in optoelectronics, biosensors, bioimaging and photodynamic therapy [2]. Nanomaterials may be released at various stages of their life cycle, including during production, processing, use and disposal, which creates potential pathways for their release into the environment [3]. Therefore, the growing use of ZrO_2_ and Y_2_O_3_ nanomaterials highlights the need to better understand their potential biological effects, especially during critical stages of development.

Hitherto, data on the embryotoxicity and developmental safety of ZrO_2_-based nanoparticles (NPs) remains limited. One of the studies was conducted by Karthiga et al. [4], who exposed Danio rerio embryos to ZrO_2_ NPs from 24 to 96 h post-fertilisation at concentrations of 0.5, 1, 2, 3, 4, and 5 µg/mL. Increased embryo mortality was observed even at the lowest concentration, while exposure to concentrations from 1 µg/mL resulted in a high incidence of malformations, which frequently led to embryo death. In contrast, Sajtos et al. [5], using the same model organism, investigated three types of NPs consisting of a Si core and a Zr shell. They reported that these NPs, administered at 5, 10, and 20 µg/mL, did not affect zebrafish body weight and resulted in 100% survival in all experimental groups. A study on ZrO_2_ NP embryotoxicity was performed in rats by Lyashenko et al. [6]. In this study, pregnant females were orally exposed to ZrO_2_ NPs with a dose of 100 mg/kg body weight/day from gestation day 1 to day 20. Although no increase in foetal malformations or postnatal mortality was observed, offspring showed reduced body weight 14 days after birth and disturbances in liver metabolism two months after birth.

Data on the embryotoxicity of Y_2_O_3_ NPs is even more limited, despite growing interest in their physicochemical and biomedical potential. Yttrium oxide is an attractive material due to its high dielectric constant, high mechanical strength, high melting point, and good chemical stability [7]. In addition, Y_2_O_3_ is characterised by a wide band gap, high refractive index, and favourable optical properties, which support its use as an optical material [8].

From a biological perspective, Y_2_O_3_ NPs have been reported to exhibit antibacterial activity. Kannan and Sundrarajan [9], using Acalypha indica extract for green synthesis of Y_2_O_3_ NPs, observed antibacterial effects against Escherichia coli, Pseudomonas aeruginosa, Serratia marcescens, and Staphylococcus aureus. Moreover, Y_2_O_3_ NPs have been shown to exert hepatoprotective effects in a mouse model of acute liver failure at a dose of 250 mg/kg, reducing MDA levels, increasing glutathione peroxidase activity, preventing a decrease in SOD activity, suppressing NF-κB pathway activation, and alleviating histological liver damage [10]. The only available embryotoxicity study by Alhusayni et al. [11] investigated Y_2_O_3_ NPs in a mouse in vitro fertilisation and embryo culture model, using two concentrations, 25 and 50 µg/mL. The authors showed that, despite no effect on early cleavage, the higher concentration impaired blastocyst development, suggesting that Y_2_O_3_ NPs may affect later developmental processes. Therefore, studies using models that enable the analysis of later stages of embryo development are needed to evaluate their impact beyond the earliest preimplantation stages, particularly during tissue differentiation and organogenesis.

Lack of comprehensive or, in the case of Y_2_O_3_, even incidental studies raises a significant question about the safety of exposure to ZrO_2_ and Y_2_O_3_ NPs during pregnancy and embryonic development. The chicken embryo serves as a useful in ovo vertebrate model for studying the effects of nanoparticles during development, allowing direct exposure of the embryo regardless of the mother’s metabolism and transport through the placenta. This article aims at bridging this gap and highlights the need for further, more systematic studies using well-characterised NPs and comparable experimental conditions.

## 2. Materials and Methods

### 2.1. Hydrothermal Preparation of Rare-Earth-Doped Oxide NPs

Rare-earth-doped oxide NPs with distinct morphologies were synthesised using a microwave-assisted hydrothermal method. The experimental conditions were tailored to obtain elongated Y_2_O_3_ and quasi-spherical ZrO_2_ NPs.

The Y_2_O_3_ sample, containing 0.5 mol% europium ions, was prepared from aqueous solutions of yttrium(III) nitrate hexahydrate (Y(NO_3_)_3_ּ 6H_2_O, Sigma-Aldrich, Poznan, Poland) and europium(III) nitrate pentahydrate (Eu(NO_3_)_3_ּּּ 5H_2_O, Sigma-Aldrich, Poznan, Poland). The precursor salts were dissolved in distilled water under continuous magnetic stirring at room temperature until completely dissolved. Subsequently, a potassium hydroxide solution (1 M KOH) was added dropwise to the reaction mixture to induce precipitation and adjust the pH level to 10.

ZrO_2_ NPs were synthesised using zirconium tetrachloride (ZrCl_4_, Sigma-Aldrich) as the zirconium precursor. Terbium(III) nitrate hexahydrate (Tb(NO_3_)_3_·6H_2_O, Sigma-Aldrich Poznan, Poland) and yttrium(III) nitrate tetrahydrate (Y(NO_3_)_3_·4H_2_O) were used as the terbium and yttrium precursors, respectively. The precursor amounts were selected to yield a nominal composition of 0.5 mol% Tb and 3 mol% Y with respect to ZrO_2_. The precipitation process was performed by the gradual addition of potassium hydroxide (1 M KOH) until a pH of 9 was reached.

The resulting suspensions were transferred into Teflon-lined reaction vessels and subjected to microwave-assisted hydrothermal treatment in a Magnum II reactor (Ertec, Wroclaw, Poland) equipped with a 700 W microwave heating system. The operating parameters, including temperature, pressure, and reaction time, were continuously monitored using Magnum V2 software (version 1.9.8.14). The reaction was conducted at a maximum temperature of 300 °C for 20 min under elevated pressure. After completion of the hydrothermal process, the products were collected and dried at 60 °C for 24 h. The dried powders were subsequently ground in an agate mortar.

To improve the crystallinity and luminescence of the materials, an additional thermal treatment was performed in air for 2 h. The europium-doped yttrium oxide NPs were annealed at 1250 °C, whereas the ZrO_2_ NPs were calcined at 1000 °C.

### 2.2. Structural, Morphological and Optical Studies

The crystal structure of the synthesised NPs was identified, and the possible occurrence of secondary crystalline phases was evaluated by X-ray diffraction (XRD) analysis. The measurements were performed using a Panalytical X’ert Powder diffractometer (Westborough, MA, USA) equipped with a Cu Kα radiation source (λ = 1.54060 Å). Diffraction patterns were recorded at room temperature within the 2Ɵ range of 20–70° using a step size of 0.05°.

The morphology and size of the obtained nanomaterials were evaluated by scanning electron microscopy (SEM) using a Hitachi SU-70 microscope (Hitachi High-Tech Corporation, Tokyo, Japan). The instrument provided a spatial resolution of 1 nm. Particle dimensions were determined from SEM images by measuring approximately 100 NPs. The results are presented as mean values with standard error of the mean.

Optical properties were studied by means of photoluminescence spectroscopy. Emission (PL) and excitation (PLE) spectra were collected at room temperature using a Horiba/Jobin-Yvon Fluorolog-3 spectrofluorometer (HORIBA Jobin Yvon, NJ, USA). The experimental setup consisted of a 450 W xenon lamp serving as the excitation source, an IHR550 monochromator (HORIBA Jobin Yvon, NJ, USA)for wavelength selection, and a Hamamatsu R928P photomultiplier tube (Hamamatsu Photonics, Iwata, Shizuoka, Japan) used as the detector.

The colloidal stability of NP suspensions was assessed through measurements of the zeta potential and hydrodynamic particle size. Analyses were performed at approximately 25 °C using a Zetasizer Ultra instrument (Malvern Panalytical, Malvern, UK) equipped with dynamic light scattering (DLS) and electrophoretic light scattering (ELS) modules. Before the measurements, the NPs were dispersed in deionised water and sonicated for 4 min using a Sonic VCCX500 ultrasonic processor (Sonic and Materials Inc., Newton, CT, USA) to minimise particle agglomeration. No pH adjustment was applied, and measurements were performed under native suspension conditions. Each sample was measured five times, and the reported values represent the mean ± standard deviation.

### 2.3. Isolation of Chicken Embryo Fibroblasts (CEFs)

Primary chicken embryo fibroblasts (CEFs) were isolated from 12-day-old chicken embryos following the protocol originally described by Hernandez and Brown [12] and further applied by Dominiak et al. [13]. Briefly, embryos were removed under aseptic conditions and dissected to separate non-fibroblast tissues. The remaining tissue was mechanically fragmented, washed with phosphate-buffered saline (PBS) containing calcium and magnesium, and then enzymatically digested with 0.25% trypsin-EDTA (Gibco Life Technologies, Paisley, UK) at 37 °C for 15–20 min.

The obtained cell suspension was filtered and centrifuged for 5 min at 1200 rpm. The cell pellet was then resuspended in Dulbecco’s Modified Eagle Medium (DMEM) with GlutaMAX™, supplemented with 10% foetal bovine serum (FBS) and 1% penicillin/streptomycin (Gibco Life Technologies, Carlsbad, CA, USA). Cells were counted, seeded at a density of 1 × 10^6^ cells per 25 cm^2^ flask, and maintained in a humidified incubator at 37 °C with 5% CO_2_.

### 2.4. Cell Viability Measurement

The cell viability experiment on 24 h CEF cultures was intended as a complementary in vitro assessment of the early cellular response to NP exposure, whereas the in ovo experiments were designed to follow the biological consequences of a single NP administration over subsequent stages of embryonic development. For the cell viability assay, CEFs were seeded in 24-well culture plates at a density of 1 × 10^6^ cells per well and cultured until they reached 80–90% confluence. The cells were then exposed to decreasing concentrations of NPs, ranging from 0.1 to 1000 µg/mL, for 24 h. After treatment, cells were detached using 0.2% trypsin-EDTA, resuspended in PBS, and stained with trypan blue (Sigma-Aldrich, Burlington, MA, USA).

Cell viability was assessed using an automated Olympus Cell Counter R1 (Olympus, Warsaw, Poland) and expressed as the percentage of viable cells. The experiment was performed in four replicates.

### 2.5. In Ovo Experimental Model

The in ovo experiment does not represent chronic or repeated exposure, as the NPs were administered only once, on the first day of incubation, and the effects of this single exposure were subsequently evaluated at different developmental time points. ZrO_2_ and Y_2_O_3_ NPs were used for biological studies at two concentrations: 10 µg/mL and 100 µg/mL, prepared in PBS. The experiments were performed using an in ovo model of a chicken egg [13]. After a small hole was drilled in the eggshell, 100 µL of the NP suspension was administered into the air chamber of fertilised Ross 308 eggs. The opening was then sealed with Parafilm. The control group (CTRL) received 100 µL of PBS. Eggs were incubated in commercial egg incubators at 39 °C and 70% relative humidity, with automatic egg rotation every hour. Embryo mortality was assessed on the day of sample collection, with no differences found between the nanoparticle-treated groups and the control group.

According to current regulations, approval from the local ethics committee was not required, as sample collection was completed before day 12 of embryonic development.

For microarray experiment RNA was isolated from chicken embryos from days 4 and 5 of incubation.

To assess oxidative stress, superoxide dismutase (SOD) activity and the contents of protein carbonyls (CP) and malondialdehyde (MDA) were measured in chicken embryos between days 4 and 10 of incubation. The number of embryos required for each biological replicate depended on the day of incubation and the embryo’s weight. At earlier stages of development, tissues from embryos were pooled to obtain the 100 mg quantity of material necessary to perform a single biological replicate.

### 2.6. Microarray Experiment

In this part of the experiment, RNA was isolated from chicken embryos incubated 4 and 5 days after treatment with 100 µL of ZrO_2_ or Y_2_O_3_ NPs suspended in PBS (suspension concentration 10 µg/mL). The control group was treated with 100 µL of pure PBS. Total RNA was extracted using the RNeasy Mini Kit (Qiagen, Hilden, Germany). During RNA isolation DNase (RNase-Free DNase Set, Qiagen, Hilden, Germany) was used to purify the isolated RNA from DNA contamination. The entire procedure was performed according to the manufacturer’s instructions. The NanoDrop™ 2000 spectrophotometer (Thermo Fisher Scientific, Waltham, MA, USA) was used to assessment of RNA purity and concentration. RNA integrity was verified using the Agilent Bioanalyzer 2100 with the Agilent RNA 6000 Nano Kit (Agilent Technologies, Palo Alto, CA, USA). Samples showed revealing high integrity (RIN = 10).

Twenty-four gene expression microarrays were performed to identify genes with altered expression. The experiment utilised Chicken (V2) Gene Expression Microarrays, 4 × 44 k (Agilent Technologies, Santa Clara, CA, USA) and an Agilent Technologies Reagent Set according to the manufacturer’s procedure. Samples for hybridisation were prepared using the Low Input Quick Amp Labeling Kit, one-color, to amplify and label (Cy3) target RNA and to generate complementary RNA (cRNA); the RNA Spike-In Kit was used as an internal control, the Gene Expression Hybridisation Kit was also utilised. The microarrays were hybridised at 65 °C for 17 h in the hybridisation oven. After hybridisation, the microarrays were washed using dedicated buffers from the Gene Expression Wash Buffer Kit (Agilent Technologies, Santa Clara, CA, USA). Acquisition and analysis of hybridisation intensities were performed using Agilent DNA Microarray Scanner G2505C (Agilent Technologies, Santa Clara, CA, USA). Agilent Feature Extraction (FE) Software, version 10.7.3.1. was applied to extract data from microarrays and subtract the background.

### 2.7. Quantitative PCR

To validate the microarray results, the expression levels of selected genes, including *COL6A2*, encoding collagen alpha-2(VI) chain; *SDHA*, encoding succinate dehydrogenase [ubiquinone] flavoprotein subunit, mitochondrial; *PSMB7*, encoding proteasome 20S subunit beta 7; and *STEAP3*, encoding STEAP3 metalloreductase, were analysed by real-time PCR.

Commercially available primer sets were used for amplification of the selected genes: PrimePCR SYBR Green Assay, Chicken (Bio-Rad, Warsaw, Poland). The *RPL13* gene, encoding 60S ribosomal protein L13, was used as the reference gene after being identified as the most stable gene using the geNorm algorithm.

Reverse transcription was performed using the iScript cDNA Synthesis Kit (Bio-Rad), according to the manufacturer’s instructions. Real-time PCR was performed using iTaq Universal SYBR Green Supermix (Bio-Rad), on an AriaMx Real-Time PCR System (Agilent Technologies, Santa Clara, CA, USA).

The real-time PCR conditions were as follows: initial polymerase activation at 95 °C for 2 min, followed by 40 amplification cycles consisting of denaturation at 95 °C for 5 s and primer annealing/extension at 60 °C for 30 s. Relative gene expression was calculated using the 2^−ΔΔCt^ method [14].

### 2.8. Measurement of Superoxide Dismutase (SOD) Activity

SOD activity was measured with the spectrophotometric method [13,15], with modifications. A total of 100 mg of isolated chicken embryo tissues were homogenised in 0.5 mL of protein isolation buffer (PBS, 1 Dithiothreitol (DTT), 1 mM Phenylmethanesulfonyl fluoride (PMSF), 3 mM EDTA, 3 mM MgSO4). Homogenates were centrifuged 12,000× *g* for 10 min at 4 °C and the supernatant was collected to the Eppendorf tubes. To the 50 µL of protein extract, 100 µL methionine 0.19 M, 100 µL riboflavin 0.11 mM, 50 µL Nitro blue tetrazolium chloride (NBT) 1.7 mM and 500 µL PBS were added. To the blank, protein isolation buffer was added instead of protein extract. After 6 min of incubation under light, absorbance was measured at 560 nm using a CLARIOstar Plus microplate reader (BMG LABTECH, Ortenberg, Baden-Württemberg, Germany). The results were expressed as U/µg protein. A total of 1 U of SOD activity is the amount of extract that inhibits the formation of formazan by 50%. The experiments were performed in 6 biological replicates and in 3 technical repetitions.

### 2.9. Measurement of Protein Carbonyl (CP) Content

Protein carbonyl content was determined spectrophotometrically according to the method described by Mesquita et al. [16]. Protein extracts were prepared as described above and diluted with PBS to a final concentration of 1 µg/µL. Then, 400 µL of each protein extract was mixed with an equal volume of 10 mM 2,4-dinitrophenylhydrazine (DNPH) prepared in 0.5 M phosphoric acid (H_3_PO_4_). The reaction mixtures were incubated for 10 min at room temperature. For the blank samples, 0.5 M H_3_PO_4_ without DNPH was used.

After incubation, 200 µL of 6 M NaOH was added to each sample, followed by an additional 10 min incubation at room temperature. Absorbance was measured at 450 nm using a CLARIOstar Plus microplate reader (BMG LABTECH Ortenberg, Baden-Württemberg, Germany). Protein carbonyl concentration was calculated using the molar extinction coefficient ε = 22,308 M/cm and expressed as nmol/µg protein. The analysis was performed using 6 biological replicates and 3 technical replicates.

### 2.10. Measurement of Malondialdehyde (MDA) Level

Malondialdehyde content was determined spectrophotometrically using the Lipid Peroxidation (MDA) Assay Kit, Catalog Number MAK085 (Sigma-Aldrich, Poznan, Poland), according to the manufacturer’s protocol. Isolated chicken embryo tissues were homogenised on ice in MDA lysis buffer containing Butylated hydroxytoluene (BHT). The homogenates were then centrifuged at 13,000× *g* for 10 min.

The samples and MDA standards were mixed with thiobarbituric (TBA) acid solution and incubated at 95 °C for 60 min. After incubation, the samples were cooled to room temperature in an ice bath. The absorbance of the coloured reaction product was measured at 532 nm using CLARIOstar Plus microplate reader (BMG LABTECH, Ortenberg, Baden-Württemberg, Germany). MDA concentration was calculated from the standard curve and expressed as nmol/mg tissue. The experiments were performed in 6 biological replicates and in 3 technical repetitions.

### 2.11. Confocal Microscopy

To assess the fluorescence of the NPs and to confirm the distribution of ZrO_2_ and Y_2_O_3_ NPs within the tissues of developing embryos, confocal microscopy was employed. Chicken embryos were collected on days 5, 7, and 10 of incubation. The embryos were fixed in formalin for 24 h. and subsequently transferred to 70% ethanol. For paraffin embedding, the samples were processed through a graded ethanol series (70%, 80%, 96%, 100%), followed by xylene, and finally infiltrated with paraffin using a tissue processor (STP 120, Microm, Walldorf, Germany). The paraffin-embedded blocks were sectioned at a thickness of 5 µm. The prepared sections were then deparaffinised and stained with Hoechest 33342 (Biotium, CA, USA) to visualise cell nuclei. Imaging was performed at 20x lens under FV-500 Olympus Scanning Confocal System (Olympus, Warsaw, Poland). The following excitation-emission filters were used: 405 nm vs. 430–450 nm; 488 nm vs. 505–525 nm; 543 nm vs. 560 IF. Confocal microscopy figures were combined in Adobe Photoshop CS6; original microphotographs are attached as a Supplementary Materials (Figures S1–S24).

### 2.12. Statistical Analysis

For statistical evaluation, all obtained data were first assessed for homogeneity of variance and Gaussian distribution using the Kolmogorov–Smirnov test. Based on the distribution of the data, statistical comparisons were performed using one-way ANOVA followed by Tukey’s post hoc test for normally distributed data, or the Mann–Whitney test for non-Gaussian data. All statistical analyses were performed using GraphPad InStat 3.10 software. Differences were considered statistically significant at *p* ≤ 0.05, highly significant at *p* ≤ 0.01 or *p* ≤ 0.001, while *p* ≤ 0.1 was interpreted as a trend.

The statistical analysis of microarrays was performed using the Gene Spring 14.9.1 software platform (Agilent Technologies, Santa Clara, CA, USA). The probe sets were filtered by flags to remove poor-quality probes (absent flags). The statistical significance of the differences observed was evaluated using Moderated T-test (*p* < 0.05). Multiple testing correction was performed using Benjamini and Hochberg False Discovery Rate (FDR) < 0.05. Microarray data were deposited in the Gene Expression Omnibus data repository under the number GSE326080.

## 3. Results

### 3.1. Structural and Phase Composition Analysis by X-Ray Diffraction

**Figure 1:**
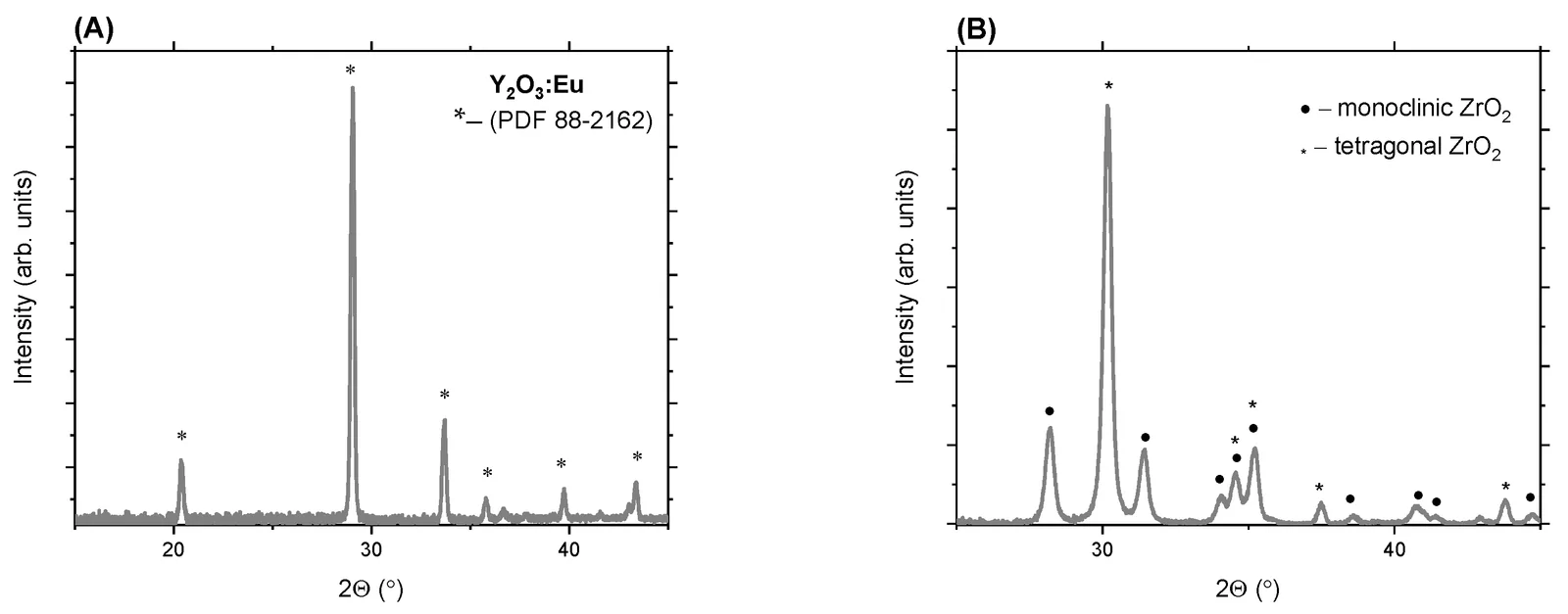
X-ray diffraction (XRD) patterns of Y_2_O_3_: Eu (**A**) and ZrO_2_:Tb, Y samples (**B**).

The diffraction pattern of the calcined Y_2_O_3_:Eu NPs is shown in Figure 1A and reveals the formation of a well-crystallised cubic yttrium oxide phase (PDF No. 88-2162). All observed diffraction peaks can be indexed to the cubic Y_2_O_3_ structure, and no additional reflections corresponding to residual precursor phases or other crystalline impurities were detected within the detection limits of XRD.

In contrast to the as-synthesised material, in which the presence of monoclinic yttrium hydroxynitrate Y_4_O(OH)_9_(NO_3_) was previously identified, thermal treatment resulted in a complete phase transformation into crystalline Y_2_O_3_. Figure 1B shows the XRD pattern of ZrO_2_:Y, Tb (3 mol.% of yttrium) after annealing at 1000 °C for 2 h. The diffraction peaks can be assigned to two zirconia polymorphs: the monoclinic baddeleyite (space group P2_1_/c) and tetragonal phases (space group P4_2_/nmc). The reflections corresponding to the monoclinic phase are located at 2Ɵ: 28.2°,31.5°,34.1°, 34.4°, 35.3°, 38.6°, 40.8°, 41.2°, 41.4°; and they are consistent with the reference pattern PDF No. 00-007-0343 [17]. The tetragonal zirconia phase is identified by reflections at 2Ɵ: 30.1°, 34.6°, 35.2°, 37.5°, 43.8°; this is in agreement with PDF No. 01-079-1767 [18]. The most intense diffraction peak, located at 2Ɵ = 30.2°, corresponds to the (101) reflection of tetragonal zirconia, suggesting a significant contribution of the tetragonal phase in the obtained material.

### 3.2. Morphology and Particle Size Analysis

**Figure 2:**
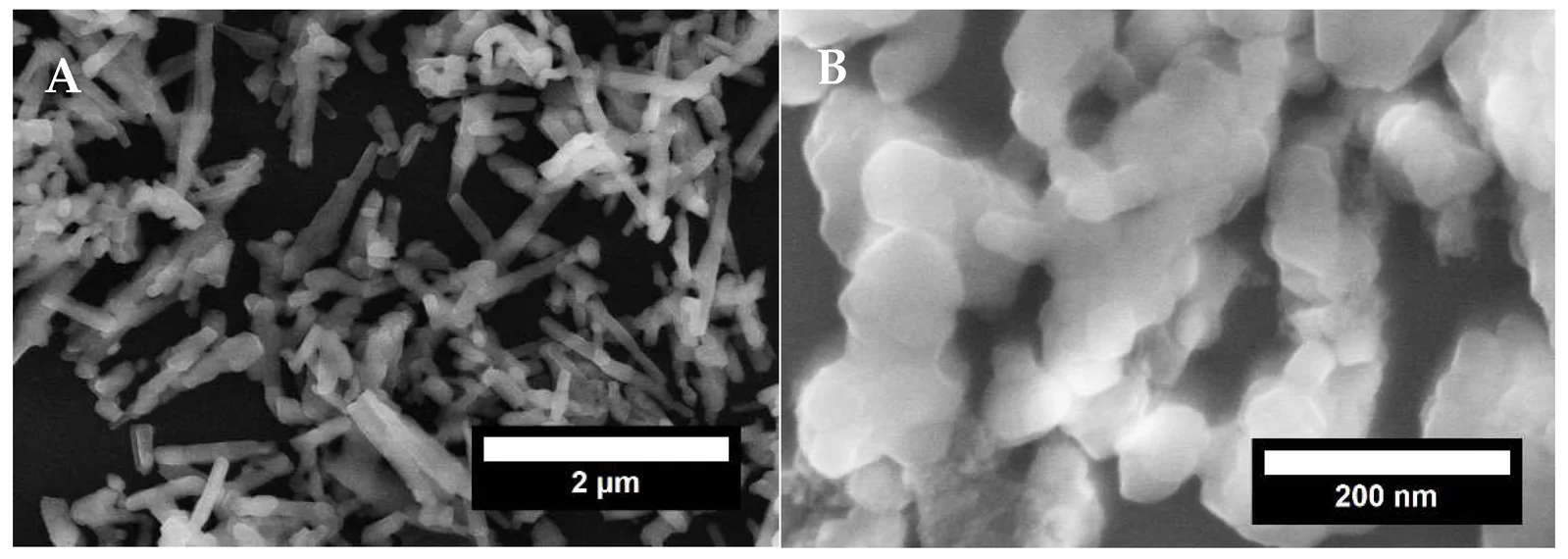
Representative SEM images of Y_2_O_3_:Eu (**A**) and Y-stabilised Tb-doped ZrO_2_ NPs (**B**).

Representative micrographs of scanning electron microscopy for calcined Y_2_O_3_:Eu NPs are shown in Figure 2A. The synthesised material consists predominantly of elongated, rod-like NPs with a relatively uniform morphology. Partial agglomeration of the particles is observed. Nevertheless, individual NPs remain readily distinguishable, allowing estimation of their dimensions.

**Figure 3:**
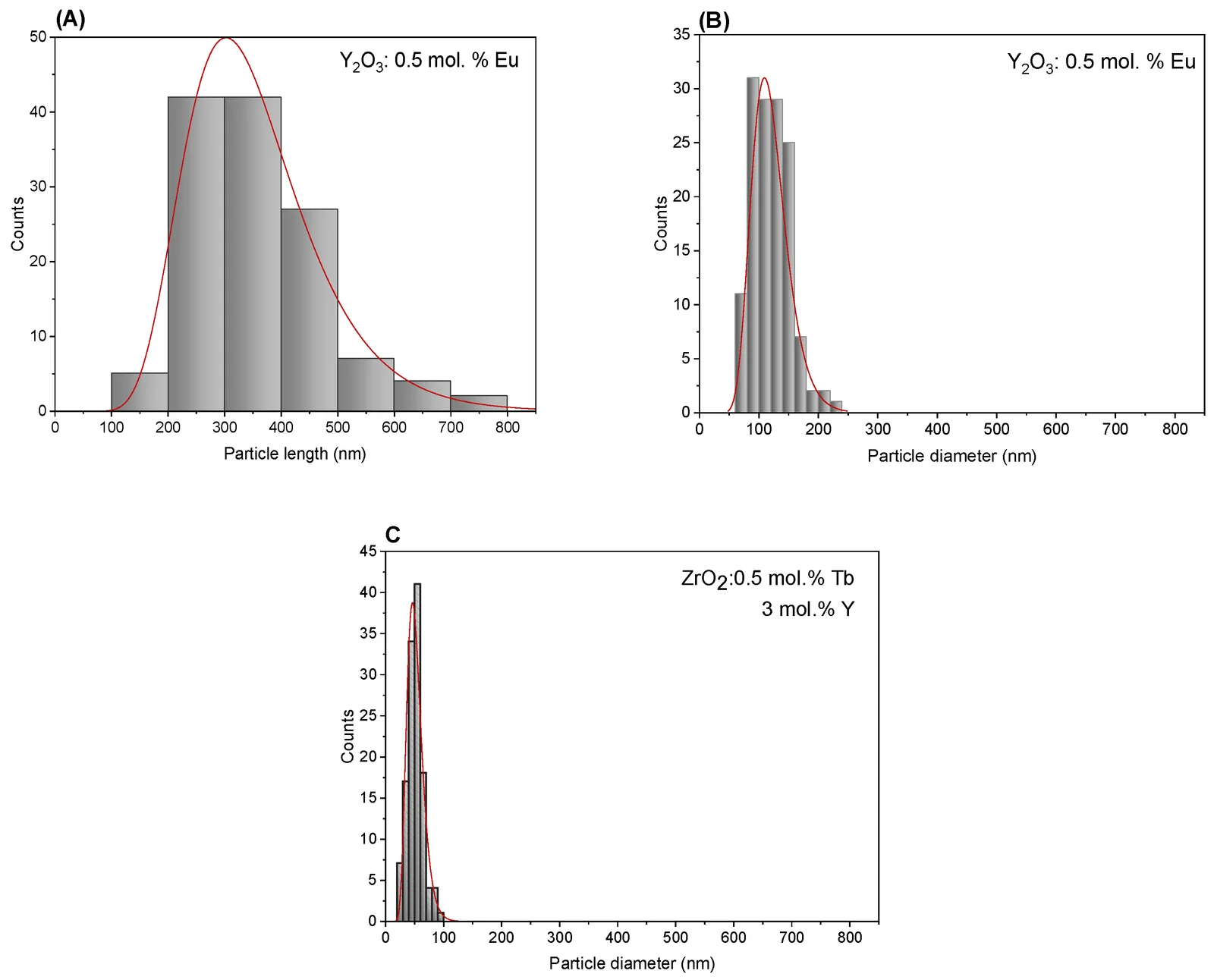
Histograms of particle length (**A**) and diameter (**B**) of calcined Y_2_O_3_: Eu NPs and particle size distribution of Y-stabilised Tb-doped ZrO_2_ NPs (C) ZrO_2_: Y, Tb sample (**C**) obtained from SEM image analysis. The solid red lines represent log-normal fits.

The average nanoparticle length and diameter were determined to be (354 ± 10) nm and (120 ± 3) nm, respectively, corresponding to an aspect ratio of approximately 3. The particle length and diameter distributions obtained from SEM image analysis are presented in Figure 3A and Figure 3B respectively. Both histograms exhibit a unimodal asymmetric character and are well described by a log-normal function. The diameter distribution is narrower than the corresponding length distribution, indicating lower variability in the lateral dimensions of the NPs.

The SEM image of yttrium-stabilised (3 mol% Y_2_O_3_) and Tb^3+^-doped ZrO_2_ NPs after thermal treatment at 1000 °C (Figure 2B) reveals agglomerated structures with quasi-spherical morphology. Individual NPs are difficult to distinguish, indicating partial coalescence into larger aggregates. As a result of calcination, intergranular boundaries became less visible. The SEM image (Figure 2B) shows irregularly shaped, rounded grains with no distinct sharp edges.

The particle size distribution obtained from SEM image analysis is presented in Figure 3C. The histogram reveals a relatively narrow, unimodal distribution of particle diameters. The calculated mean nanoparticle size was (52 ± 5) nm. Following thermal treatment, the average particle diameter increased by approximately 10 nm compared with the as-grown NPs.

### 3.3. Colloidal Properties and Stability of NPs in Aqueous Suspensionorphology and Particle Size Analysis

**Figure 4:**
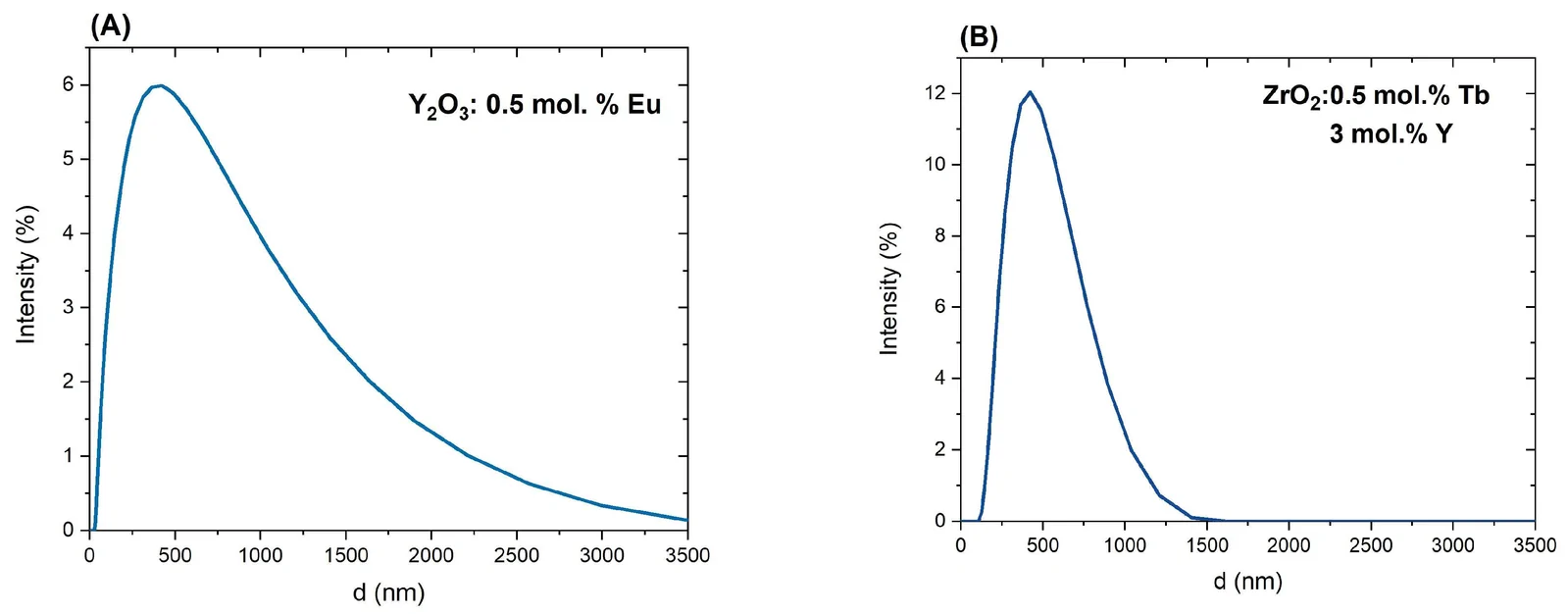
Hydrodynamic size distributions of Y_2_O_3_:Eu (**A**) and Y-stabilised ZrO_2_:Tb NPs (**B**) determined by dynamic light scattering (DLS).

The hydrodynamic size distribution and zeta potential of the synthesised NPs are summarised in Table 1. The Eu-doped Y_2_O_3_ NPs exhibited an average hydrodynamic diameter of 364 nm, whereas the ZrO_2_:Tb, Y NPs showed a larger average hydrodynamic diameter of 471 nm. The Y_2_O_3_:Eu suspension showed a relatively broad size distribution with a polydispersity index (PDI) of 0.6, while the zirconia-based NPs were characterised by a considerably narrower distribution (PDI = 0.2) (Figure 4).

The measured zeta potential values were +25 ± 1 mV for Y_2_O_3_:Eu and −27 ± 1 mV for the Y-stabilised Tb-doped ZrO_2_ NPs, indicating opposite surface charge characteristics of the two NP systems in an aqueous solution.

### 3.4. Optical Properties

**Figure 5:**
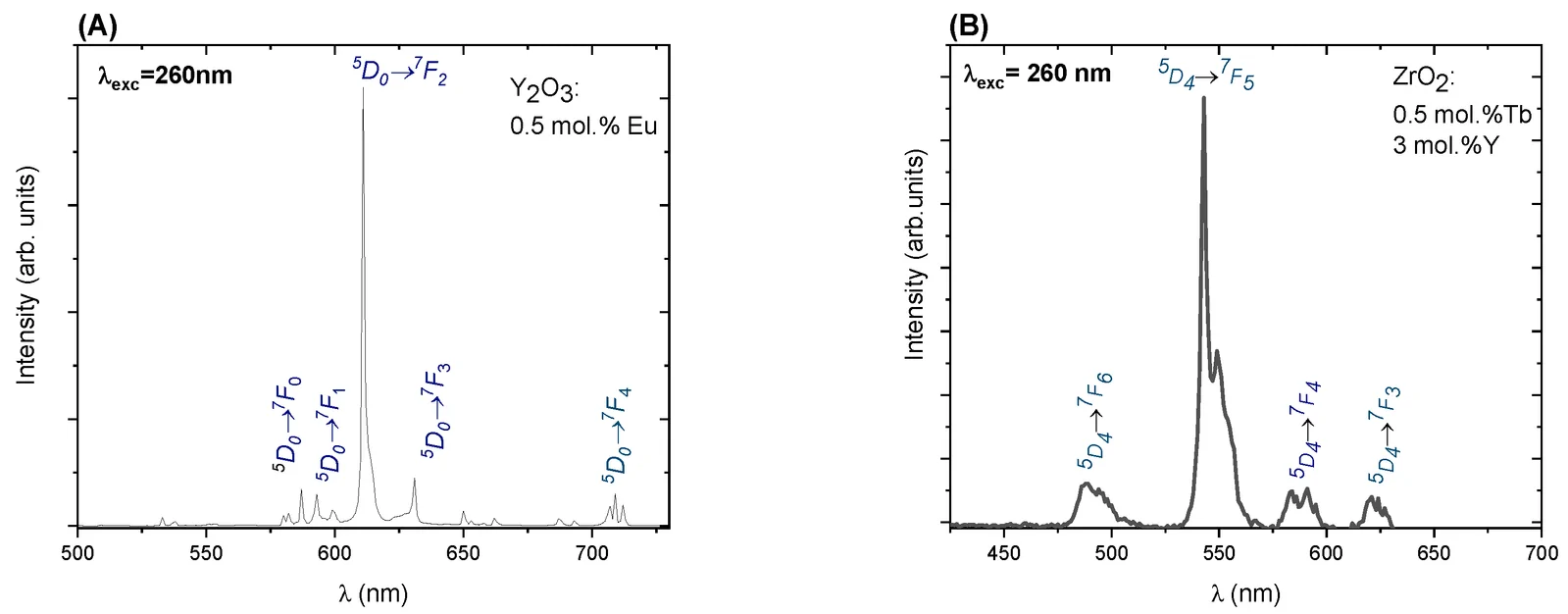
Photoluminescence emission spectra of Y_2_O_3_:Eu (**A**) and Y-stabilised ZrO_2_:Tb NPs (**B**) obtained for λ_exc_ = 260 nm. Blue annotations indicate the characteristic emission transitions of Eu^3+^ ions in (**A**) and Tb^3+^ ions in (**B**).

The room-temperature photoluminescence (PL) emission spectrum of the calcined Y_2_O_3_:Eu NPs, recorded under 260 nm excitation, is shown in Figure 5A and is dominated by a series of sharp emission lines characteristic of the intra-4f transitions of Eu^3+^ ions. The main emission peaks are located at approximately 590, 611, 630, and 710 nm, corresponding to the ^5^D_0_→^7^F_1_, ^5^D_0_→^7^F_2_, ^5^D_0_→^7^F_3_, and ^5^D_0_→^7^F_4_ transitions of Eu^3+^, respectively [19,20]. The ^5^D_0_→^7^F_2_ hypersensitive transition at approximately 611 nm is the most intense feature of the spectrum, whereas the magnetic-dipole ^5^D_0_→^7^F_1_ line at approximately 590 nm and other Eu^3+^ transitions are less intense.

The photoluminescence (PL) emission spectrum of the Y-stabilised Tb^3+^ doped zirconia after thermal treatment at 1000 °C was recorded using the same excitation wavelength (λ_exc_ = 260 nm) as for the Y_2_O_3_:Eu sample described above (Figure 5B). The spectrum exhibits several series of lines centred at approximately 490, 545, 585 and 620 nm, which were assigned to the ^5^D_4_→^7^F_6_, ^5^D_4_→^7^F_5_, ^5^D_4_→^7^F_4_ and ^5^D_4_→^7^F_3_ transitions of Tb^3+^ ions, respectively [21]. The most intense feature was observed in the green region at approximately 545 nm. It corresponds to the ^5^D_4_→^7^F_5_ transition of Tb^3+^ ions. Notably, the emission lines appear considerably broader and less distinctly resolved than those observed for the Y_2_O_3_:Eu NPs and show partially overlapping components.

**Figure 6:**
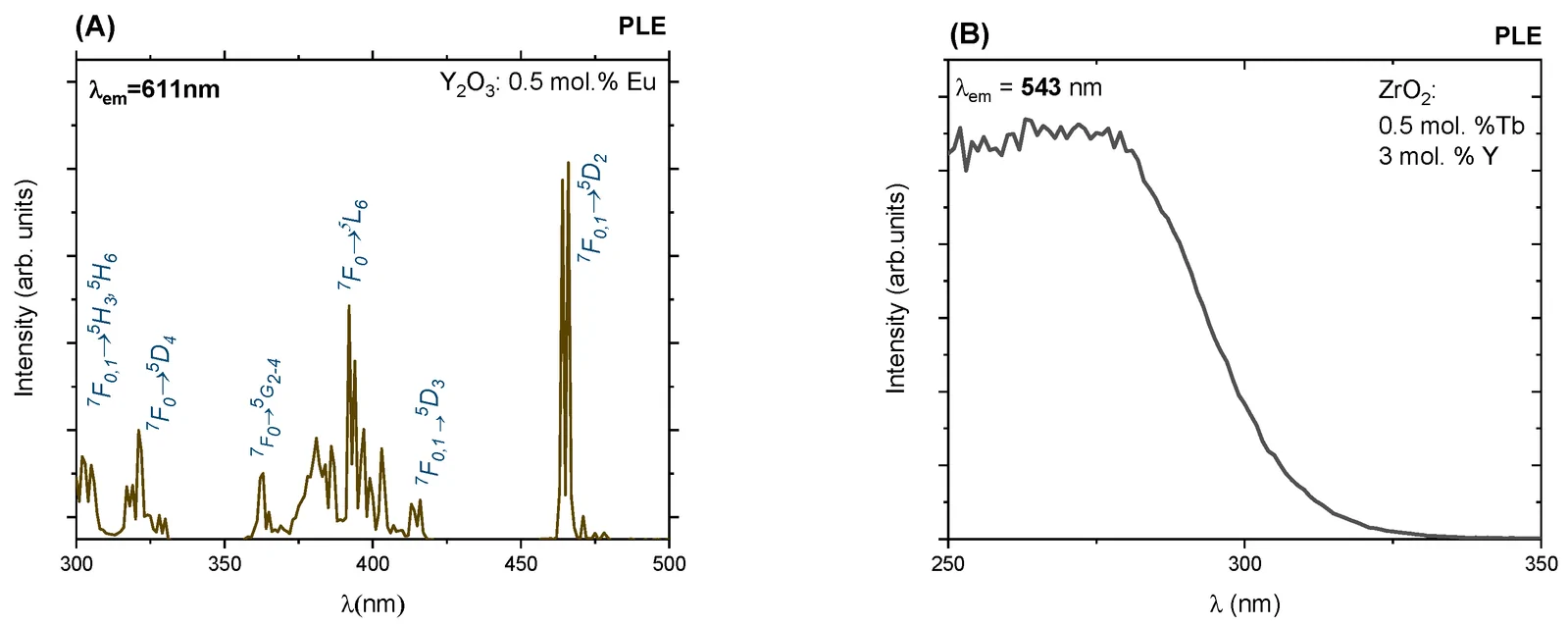
Photoluminescence excitation (PLE) spectra of Y_2_O_3_:0.5 mol% Eu obtained for λ_em_ = 611 nm (**A**) and ZrO_2_:0.5 mol% Tb, 3 mol% Y monitored at λ_em_ = 543 nm (**B**).

To further investigate the excitation mechanisms underlying the observed emission, excitation spectra were recorded by monitoring the most intense emission lines in the spectra of both samples (Figure 6).

The corresponding photoluminescence excitation (PLE) spectrum for Y_2_O_3_:Eu NPs, monitored at λ_em_ = 611 nm, is shown in Figure 6A in the 300–500 nm spectral range. It consists of a series of well-resolved, relatively narrow lines assigned to intra-configurational 4f-4f transitions of Eu^3+^ ions, with the most intense line at approximately 465 nm corresponding to the ^7^F_0,1_→^5^D_2_ transition. When the excitation spectrum was additionally recorded down to 200 nm, an intense broad band was also observed below ~ 230 nm. This band is several orders of magnitude more intense than the intra-4f excitation lines and is therefore not included in Figure 6A.

The PLE spectrum for ZrO_2_ NPs containing 0.5 mol% Tb^3+^ and 3 mol% Y^3+^ ions was recorded while monitoring the ^5^D_4_→^7^F_5_ emission at λ_em_ = 543 nm (Figure 6B). The PLE spectrum is dominated by a broad excitation band extending to 320 nm. It reaches its maximum in the deep ultraviolet region, around 260–270 nm, followed by a gradual decrease in intensity with increasing wavelength. No narrow, well-resolved lines characteristic of direct intra-configurational 4f-4f Tb^3+^ transitions were resolved within this broad excitation envelope.

**Figure 7:**
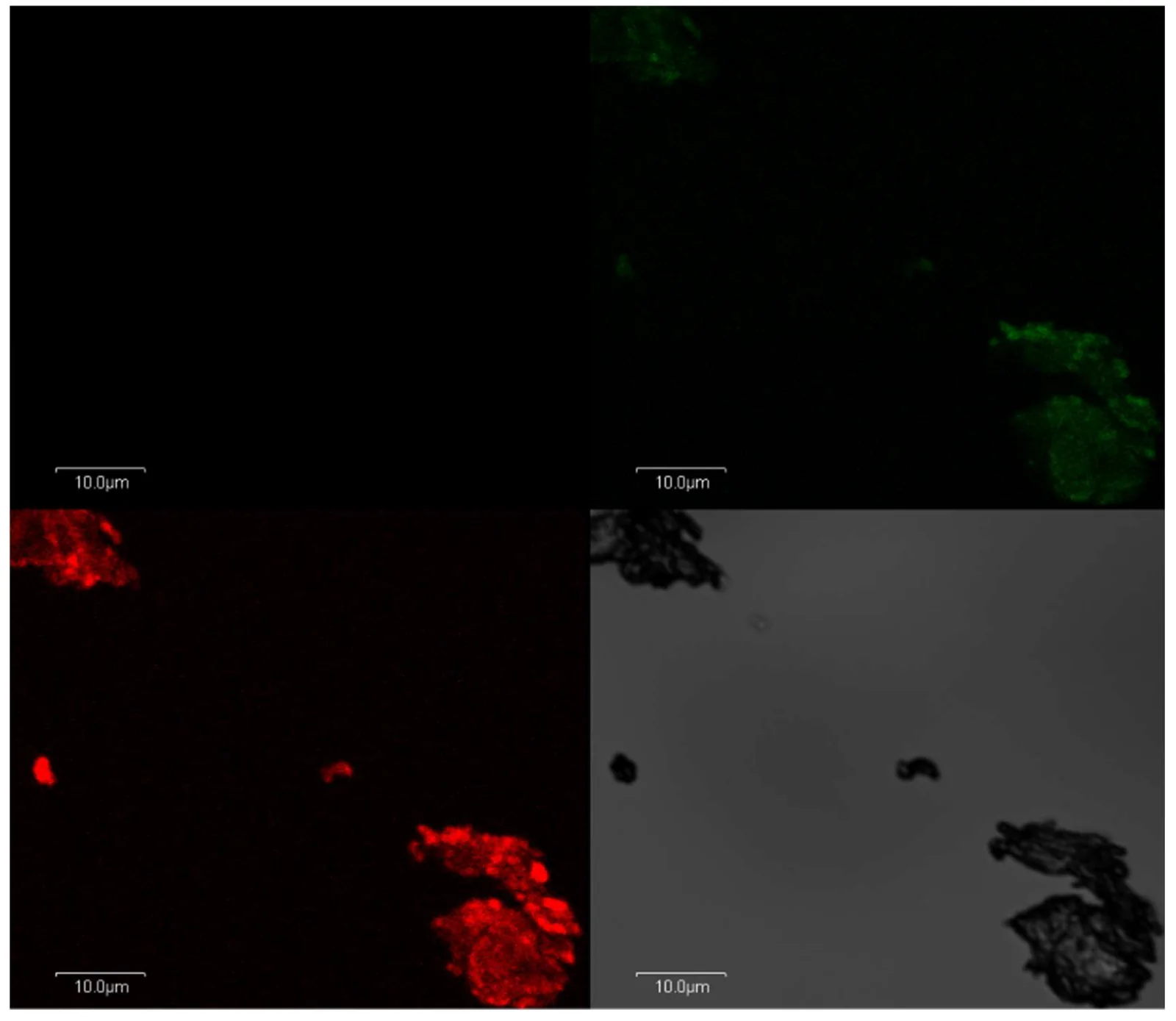
Confocal microscope images of water-dispersed Y_2_O_3_:Eu NPs showing fluorescence emission at 430–450 nm (for 405 nm excitation) - blue, 505–525 nm (for 488 nm excitation) - green, and 560 IF (for 543 nm excitation) – red. Objective magnification 40×, digital zoom 5×. Scale bar = 10 µm.

As a result of Eu doping (0.5 mol%) in the crystalline structure of Y_2_O_3_ NPs, a dominant red fluorescence emitted from the nanoparticle matrix could be observed after excitation with a 543 nm laser (Figure 7). In addition, weak green fluorescence was visible under 488 nm excitation. This green-range fluorescence was observed only in large nanoparticle aggregates and was not detected later in embryonic tissues.

**Figure 8:**
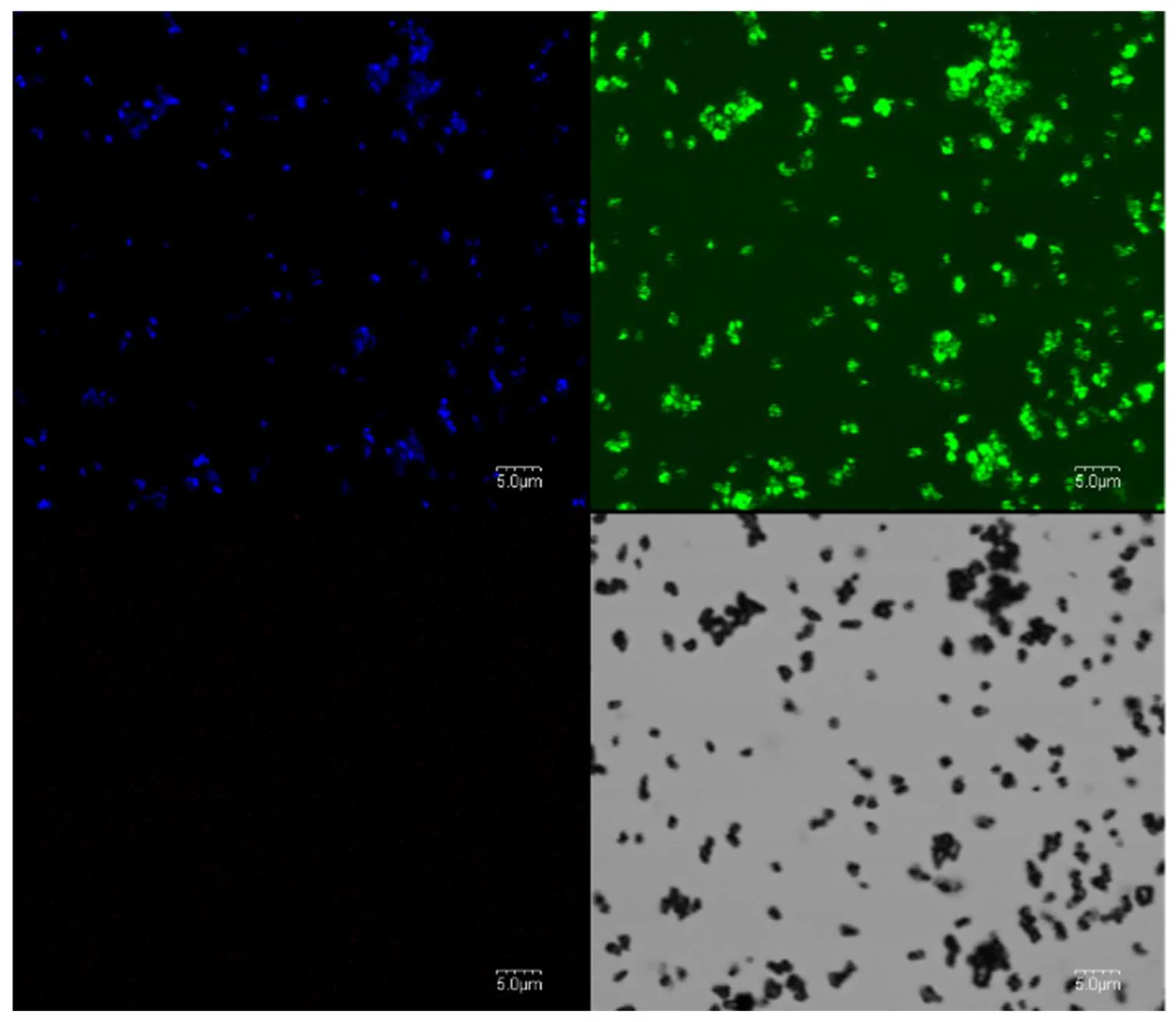
Confocal microscope images of water-dispersed ZrO_2_ NPs showing fluorescence emission at 430–450 nm (for 405 nm excitation) - blue, 505–525 nm (for 488 nm excitation) - green, and 560 IF (for 543 nm excitation) - red. Objective magnification 40×, digital zoom 5×. Scale bar = 5 µm.

Microscopic imaging (Figure 8) showed that Tb-doped ZrO_2_ NPs exhibited their main fluorescence emission in the 505–525 nm range under 488 nm excitation, which corresponds to the characteristic emission band of terbium. In addition, large nanoparticle aggregates showed fluorescence in the 430–450 nm range under 405 nm excitation, most likely associated with surface-related transition states.

### 3.5. Cell Viability Assay

**Figure 9:**
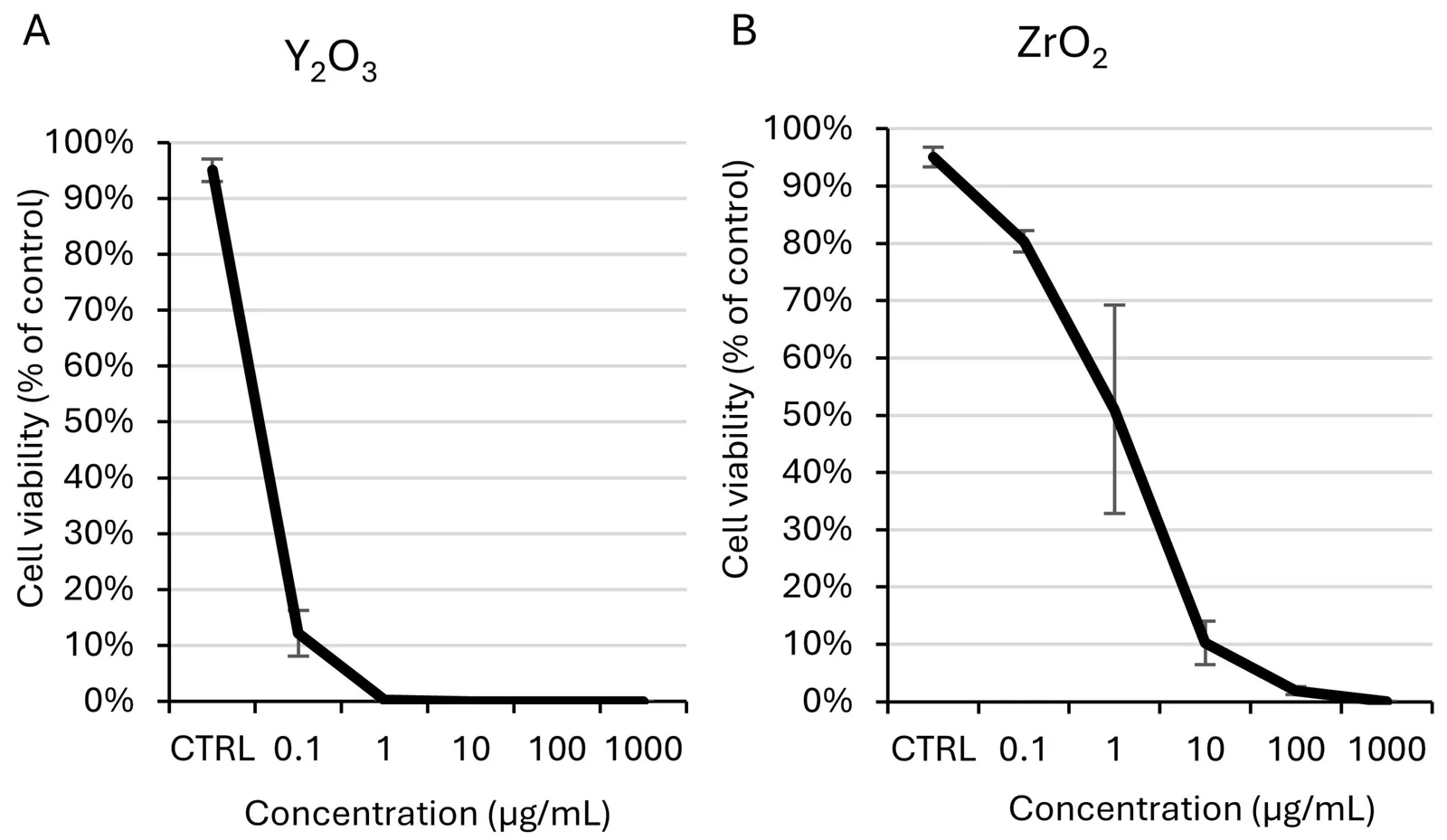
Cell viability of primary chicken embryo fibroblasts (CEFs), expressed as the percentage of viable cells, 24 h after administration of Y_2_O_3_ (**A**) and ZrO_2_ (**B**) NPs at different concentrations. For the CTRL group, the corresponding volume of PBS was added to the culture medium.

The cell viability assay showed that, after incubation with Y_2_O_3_ NPs at the lowest tested concentration of 0.1 µg/mL, CEF viability decreased to 10% (Figure 9A). The higher concentration, 1 µg/mL, resulted in 100% CEF mortality. Despite this strong cytotoxic effect observed in CEFs, no changes in embryo mortality were detected compared with the control group. Following incubation with ZrO_2_ NPs, CEF viability decreased to 50% at a concentration of 1 µg/mL (Figure 9B). At 10 µg/mL, CEF viability dropped further to 10%.

### 3.6. Fluorescence in Embryo Tissues

**Figure 10:**
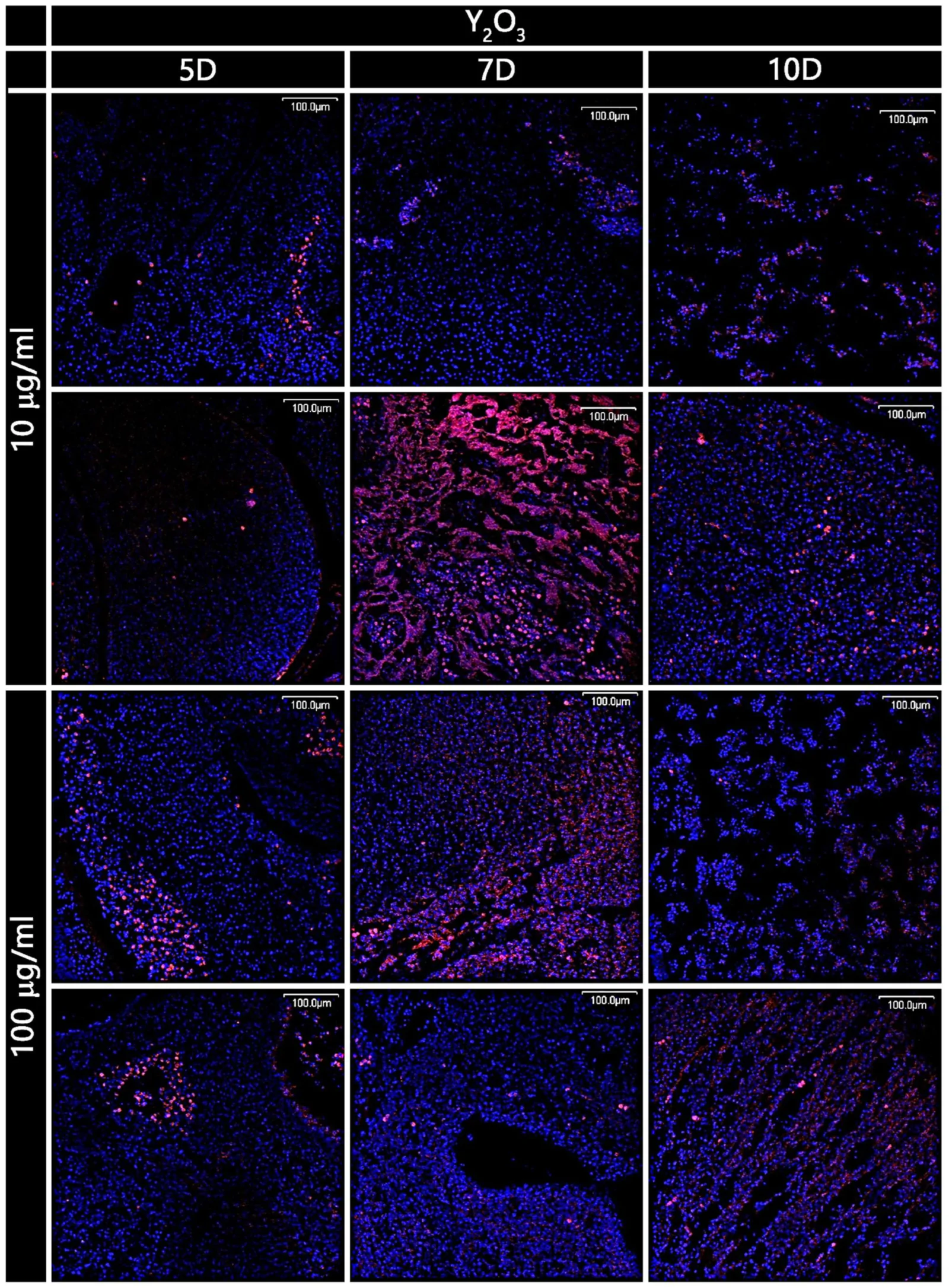
Confocal microscopy image of chicken embryo tissues after administration of Y_2_O_3_ NPs. Blue fluorescence corresponds to cell nuclei stained with Hoechst 33342, whereas red fluorescence indicates the presence of Y_2_O_3_ NPs. Objective magnification: 20×. Scale bar: 100 µm. Compilation created with the use of Photoshop CS6.

**Figure 11:**
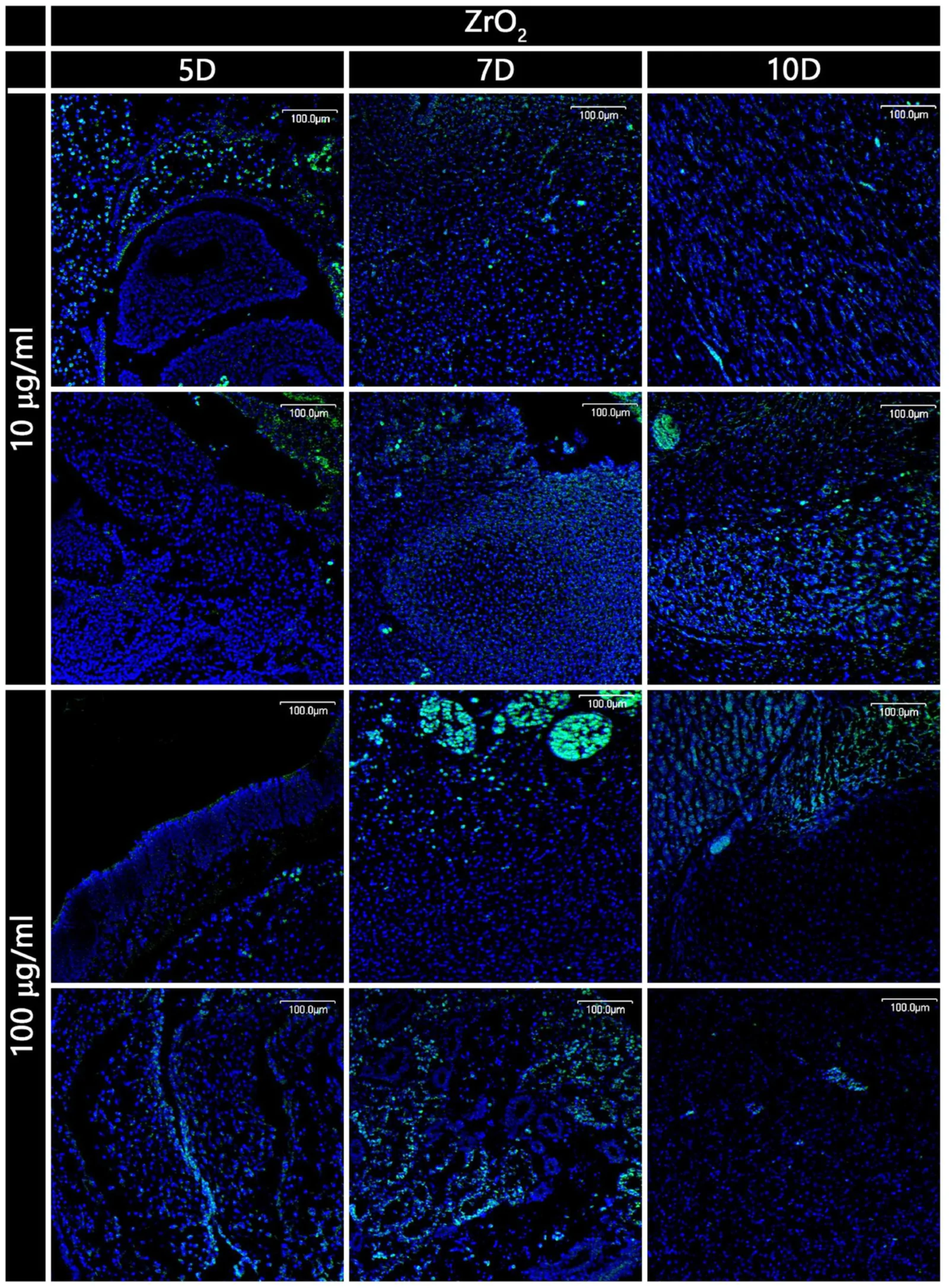
Confocal microscopy image of chicken embryo tissues after administration of ZrO_2_ NPs. Blue fluorescence corresponds to cell nuclei stained with Hoechst 33342, whereas green fluorescence indicates the presence of ZrO_2_ NPs. Objective magnification: 20×. Scale bar: 100 µm. Compilation created with the use of Photoshop CS6.

For both NPs, presence of NPs-related signal was visible in the embryonic tissues at all experimental days (Figure 10 and Figure 11). Confocal microscopy images showed that the tissue distribution of Y_2_O_3_ and ZrO_2_ NPs in chicken embryos varied between both nanoparticle concentration and incubation day. On day 7, the highest tissue concentration was observed for lower concentration (10 µg/mL) of Y_2_O_3_ NPs, and for day 5 and 7 for high (100 µg/mL) concentration (Figure 10), for ZrO_2_, the highest tissue signal was from day 5 for low concentration and days 7 and 10 for high concentration (Figure 11).

### 3.7. Activity of Superoxide Dismutase (SOD) in the Embryo

**Figure 12:**
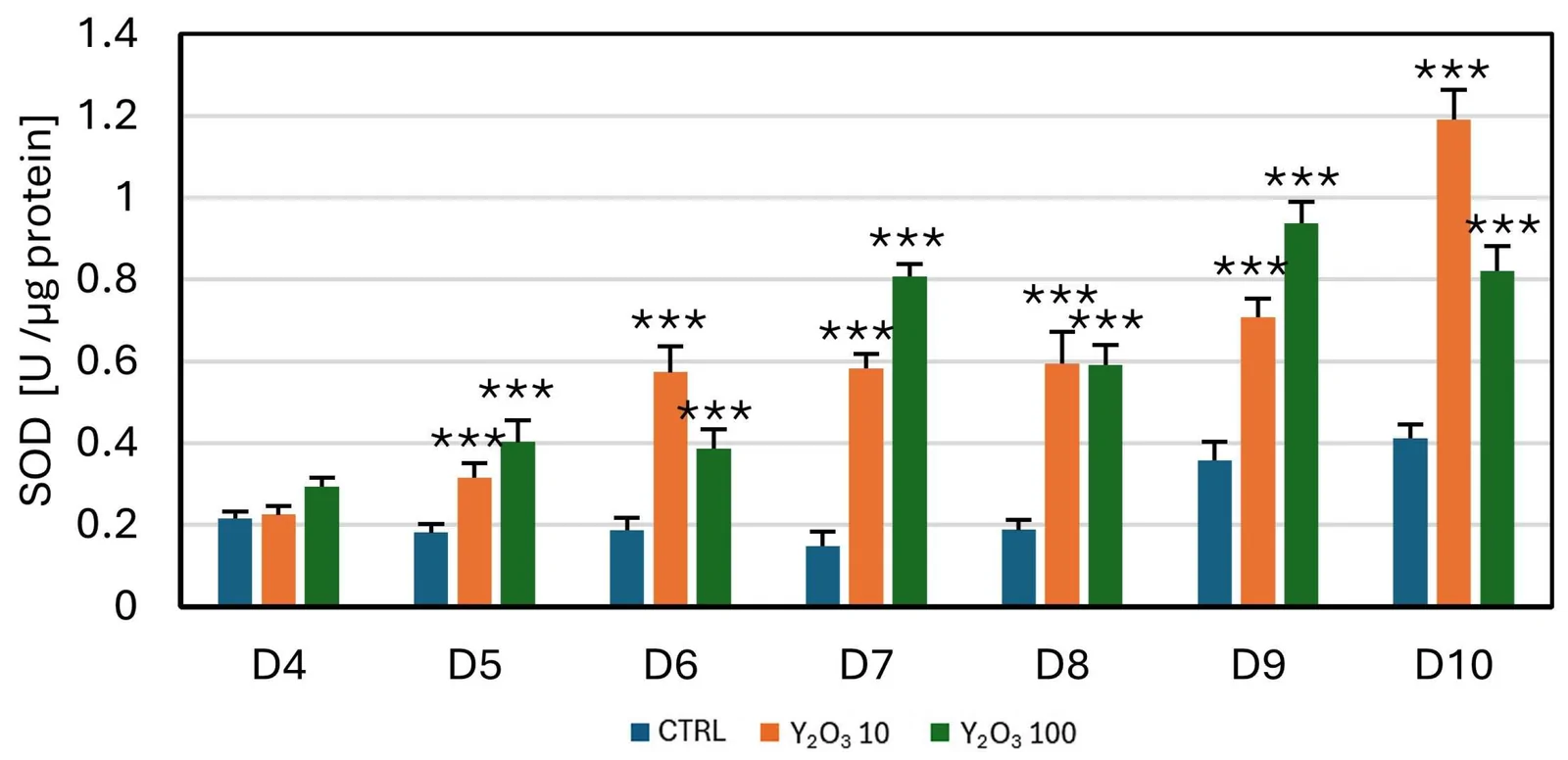
Superoxide dismutase (SOD) activity in chicken embryos from days 4 to 10 of incubation following administration of a single dose of 10 µg/mL (Y_2_O_3_ 10) or 100 µg/mL (Y_2_O_3_ 100) of Y_2_O_3_ NPs. Error bars represent standard deviations (SD). One unit of SOD activity (1 U) was defined as the amount of enzyme extract that inhibited formazan formation by 50%. Asterisks indicate significant differences compared with the CTRL group on the same day; *** *p* < 0.001.

A highly significant increase in SOD activity (Figure 12) was observed in embryos from days 4–5 to day 10 of development, regardless of the administered dose of Y_2_O_3_ NPs. The highest SOD activity was recorded on day 10 after treatment with 10 µg/mL Y_2_O_3_ NPs, reaching approximately 1.2 U/µg protein, which was about three times higher than in the control samples. On days 6 and 10, higher SOD activity was observed after administration of the lower concentration of Y_2_O_3_ NPs, whereas the opposite pattern was observed on days 5, 7 and 9.

**Figure 13:**
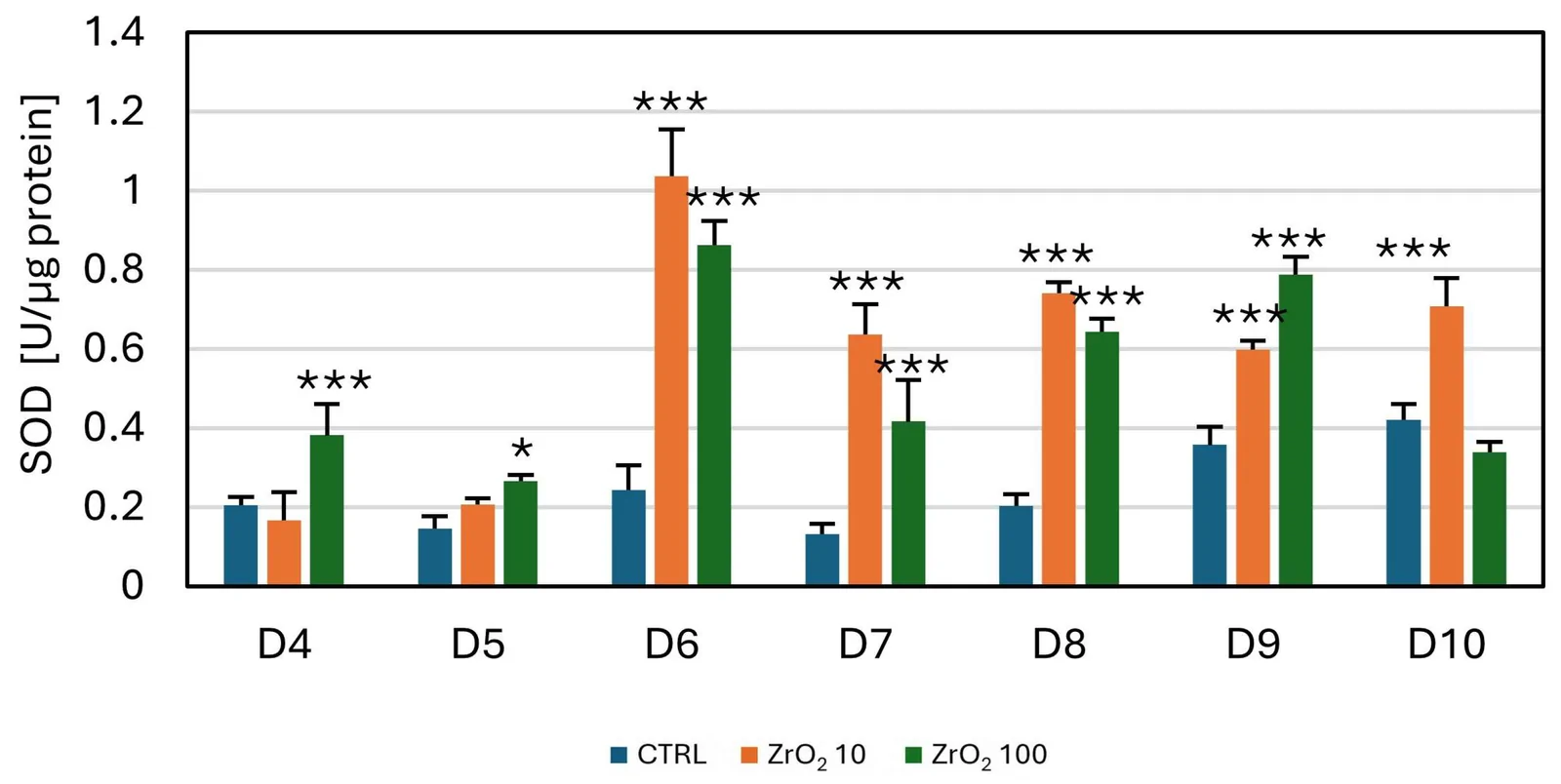
Superoxide dismutase (SOD) activity in chicken embryos from days 4 to 10 of incubation following administration of a single dose of 10 µg/mL (ZrO_2_ 10) or 100 µg/mL (ZrO_2_ 100) of ZrO_2_ NPs. Error bars represent standard deviations (SD). One unit of SOD activity (1 U) was defined as the amount of enzyme extract that inhibited formazan formation by 50%. Asterisks indicate significant differences compared with the CTRL group on the same day; * *p* < 0.05; *** *p* < 0.001.

Following administration of ZrO_2_ NPs, SOD activity (Figure 13) was significantly higher than in the CTRL group on day 4 (*p* < 0.001) and day 5 (*p* < 0.05) only after administration of the higher (100 µg/mL) concentration of NPs. From day 6 to day 9, both the lower (10 µg/mL) concentration and the higher (100 µg/mL) concentration, resulted in a highly significant increase in SOD activity when compared to CTRL. On days 6, 7 and 8, enzyme activity was higher after administration of 10 µg/mL than after 100 µg/mL. The opposite pattern was observed on day 9, when higher SOD activity was recorded for the 100 µg/mL dose. On day 10, a highly significant increase in SOD activity compared with CTRL was observed only in embryos treated with 10 µg/mL ZrO_2_ NPs.

### 3.8. Lipid Peroxidation Measured as the Content of Malondialdehyde (MDA) in the Embryo

**Figure 14:**
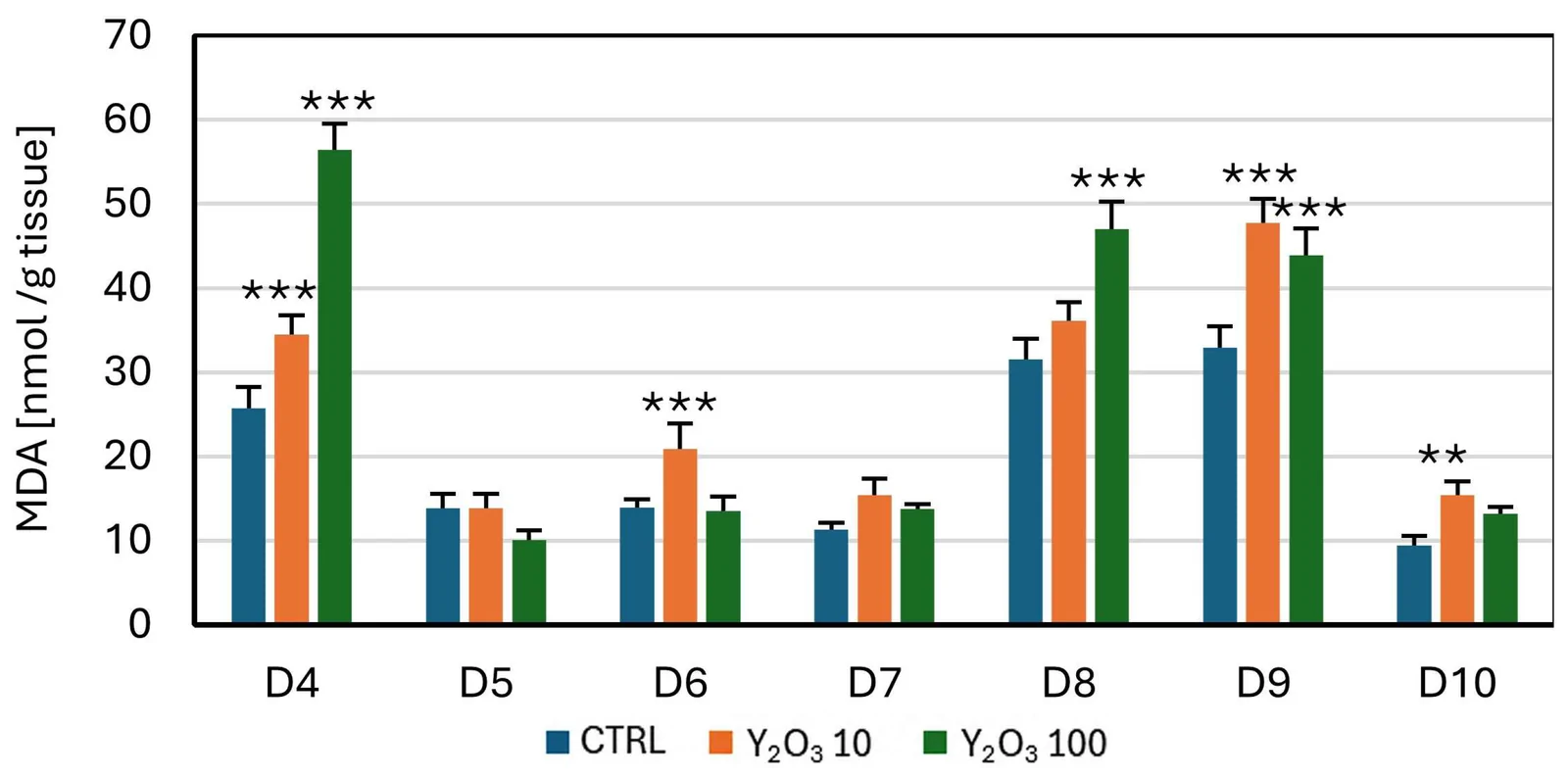
Malondialdehyde (MDA) content in chicken embryos from days 4 to 10 of incubation following administration of a single dose of 10 µg/mL (Y_2_O_3_ 10) or 100 µg/mL (Y_2_O_3_ 100) of Y_2_O_3_ NPs. Error bars represent standard deviations (SD). Asterisks indicate significant differences compared with the CTRL group on the same day;; ** *p* < 0.01; *** *p* < 0.001.

**Figure 15:**
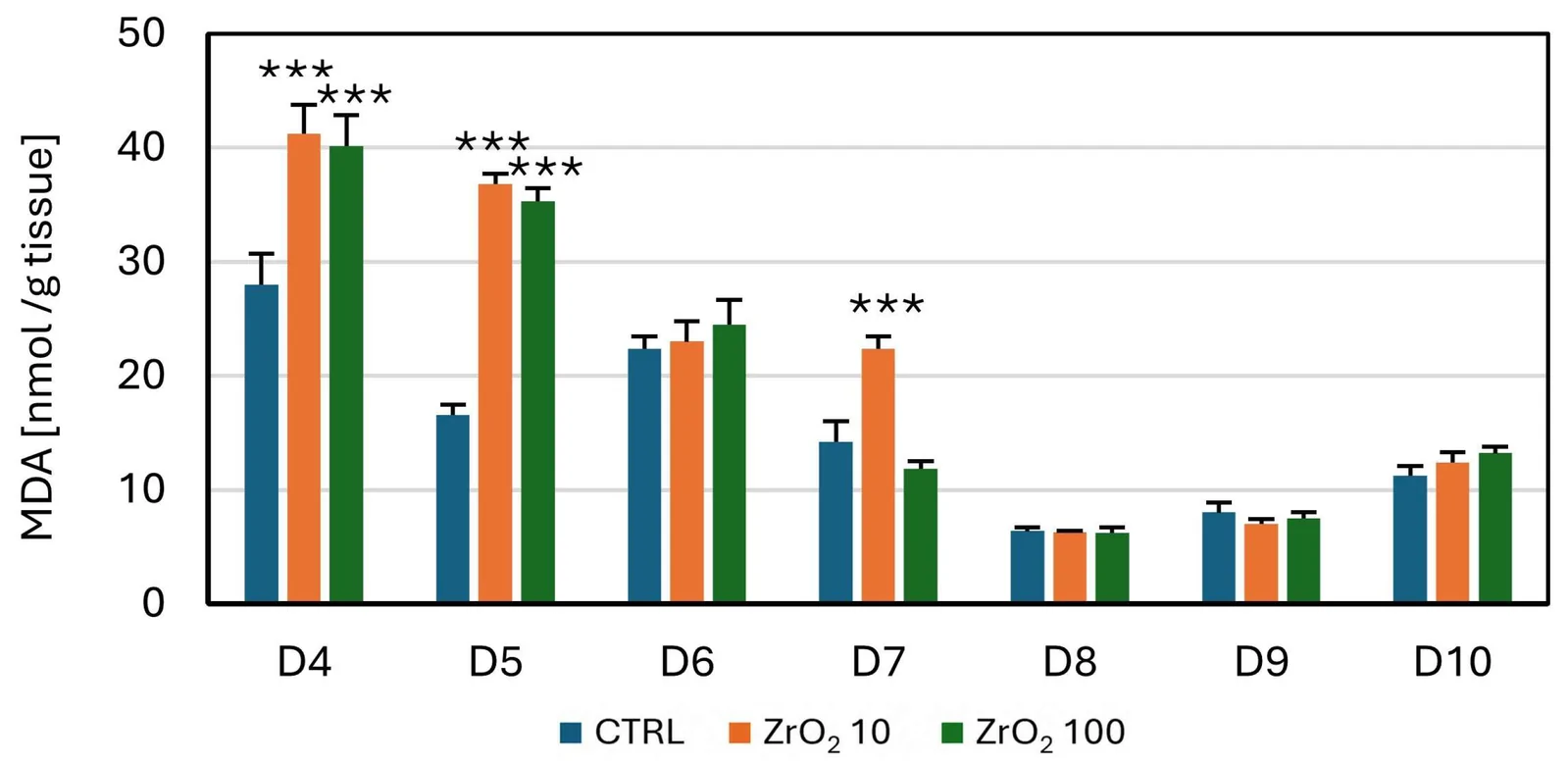
Malondialdehyde (MDA) content in chicken embryos from days 4 to 10 of incubation following administration of a single dose of 10 µg/mL (ZrO_2_ 10) or 100 µg/mL (ZrO_2_ 100) of ZrO_2_ NPs. Error bars represent standard deviations (SD). Asterisks indicate significant differences compared with the CTRL group on the same day; *** *p* < 0.001.

The most pronounced and highly statistically significant changes in MDA content were observed at the beginning of embryonic development following exposure to both NPs (Figure 14 and Figure 15). At later days of experiment, a statistically higher MDA concentration was observed at days 6 and 9 for lower dose and days 8 and 9 for higher dose of Y_2_O_3_ NPs (Figure 14), whereas for ZrO_2_ NPs (Figure 15), higher MDA content, when compared with control embryos, was recorded only on day 7 after 10 µg/mL nanoparticle administration.

### 3.9. Content of Carbonylated Proteins (CP) in the Embryo

**Figure 16:**
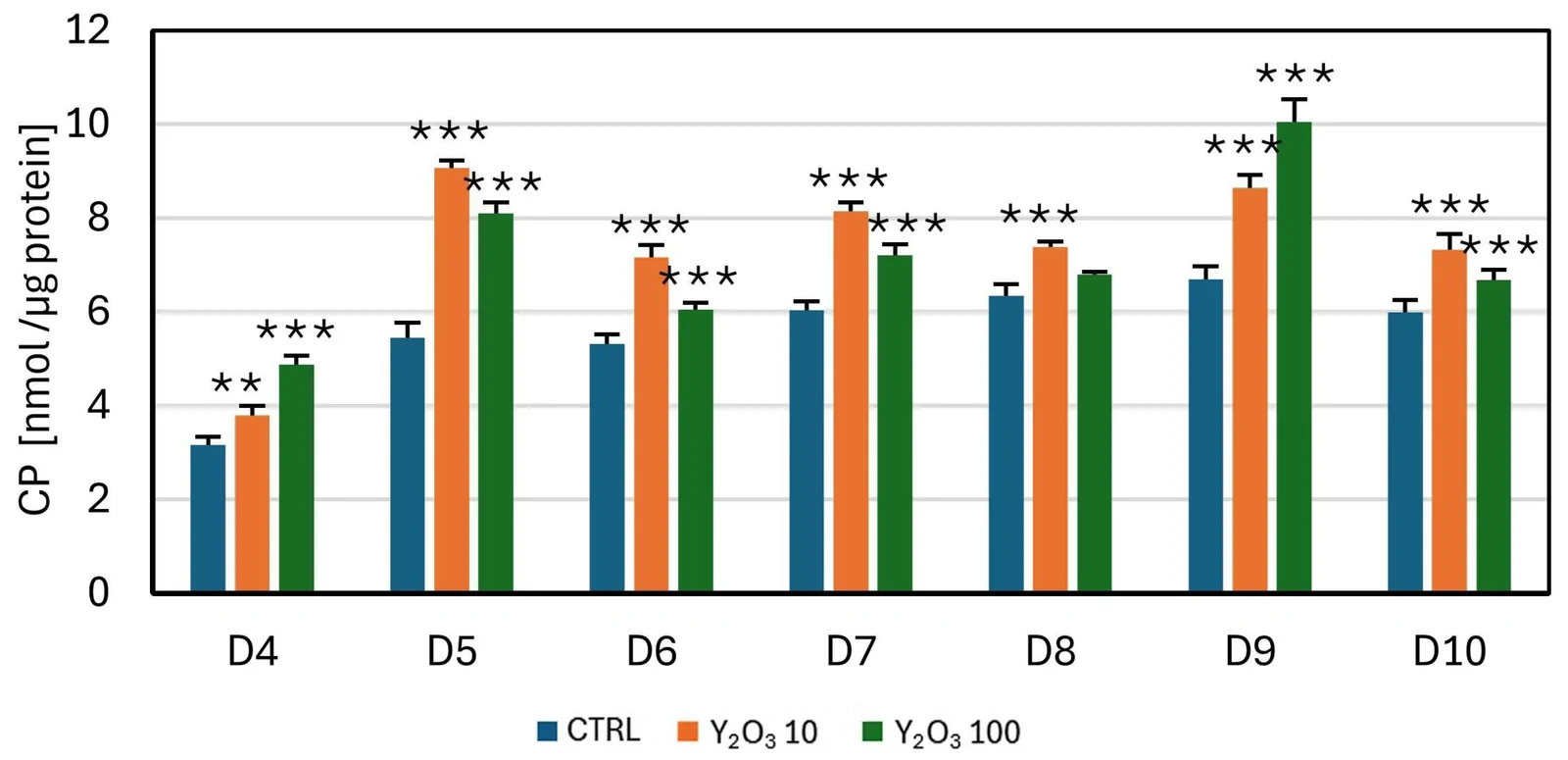
Carbonylated protein (CP) content in chicken embryos from days 4 to 10 of incubation following administration of a single dose of 10 µg/mL (Y_2_O_3_ 10) or 100 µg/mL (Y_2_O_3_ 100) of Y_2_O_3_ NPs. Error bars represent standard deviations (SD). Asterisks indicate significant differences compared with the CTRL group on the same day; ** *p* < 0.01; *** *p* < 0.001.

Carbonylated protein content showed highly significant changes at all analysed time points following incubation with both concentrations of Y_2_O_3_ NPs (Figure 16). The lower NP concentration, 10 µg/mL, had stronger effect at formation of carbonylated proteins on days 5, 6, 7, 8 and 10 of embryo development. The highest carbonylated protein content was recorded on day 9 after administration of the higher concentration of Y_2_O_3_ NPs.

**Figure 17:**
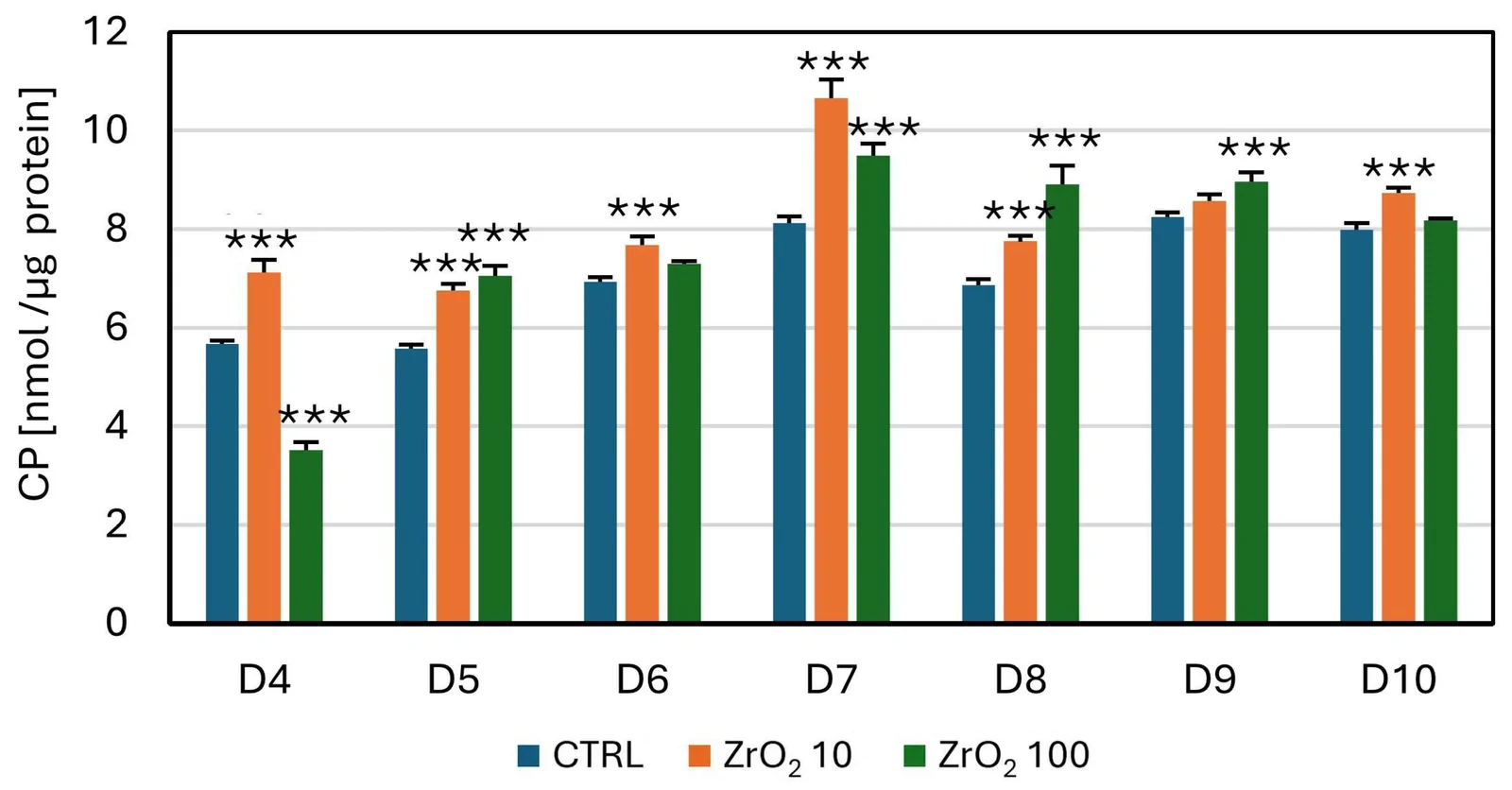
Carbonylated protein (CP) content in chicken embryos from days 4 to 10 of incubation following administration of a single dose of 10 µg/mL (ZrO_2_ 10) or 100 µg/mL (ZrO_2_ 100) of ZrO_2_ NPs. Error bars represent standard deviations (SD). Asterisks indicate significant differences compared with the CTRL group on the same day; *** *p* < 0.001.

Similarly to Y_2_O_3_ NPs, highly significant changes in carbonylated protein content after ZrO_2_ NP administration were observed in embryos from days 4 to 10 of incubation (Figure 17). However, on day 4, the lower (10 µg/mL) concentration of NPs caused a significant increase in carbonylated protein content, whereas higher (100 µg/mL) concentration of led to a significant decrease in carbonylated protein levels. On the remaining days, carbonylated protein content increased significantly after both NP doses, except for days 6 and 10 in the 100 µg/mL group and day 9 in the 10 µg/mL group, where the increase was not statistically significant.

### 3.10. Whole Genomic Microarray and Real-Time PCR

**Figure 18:**
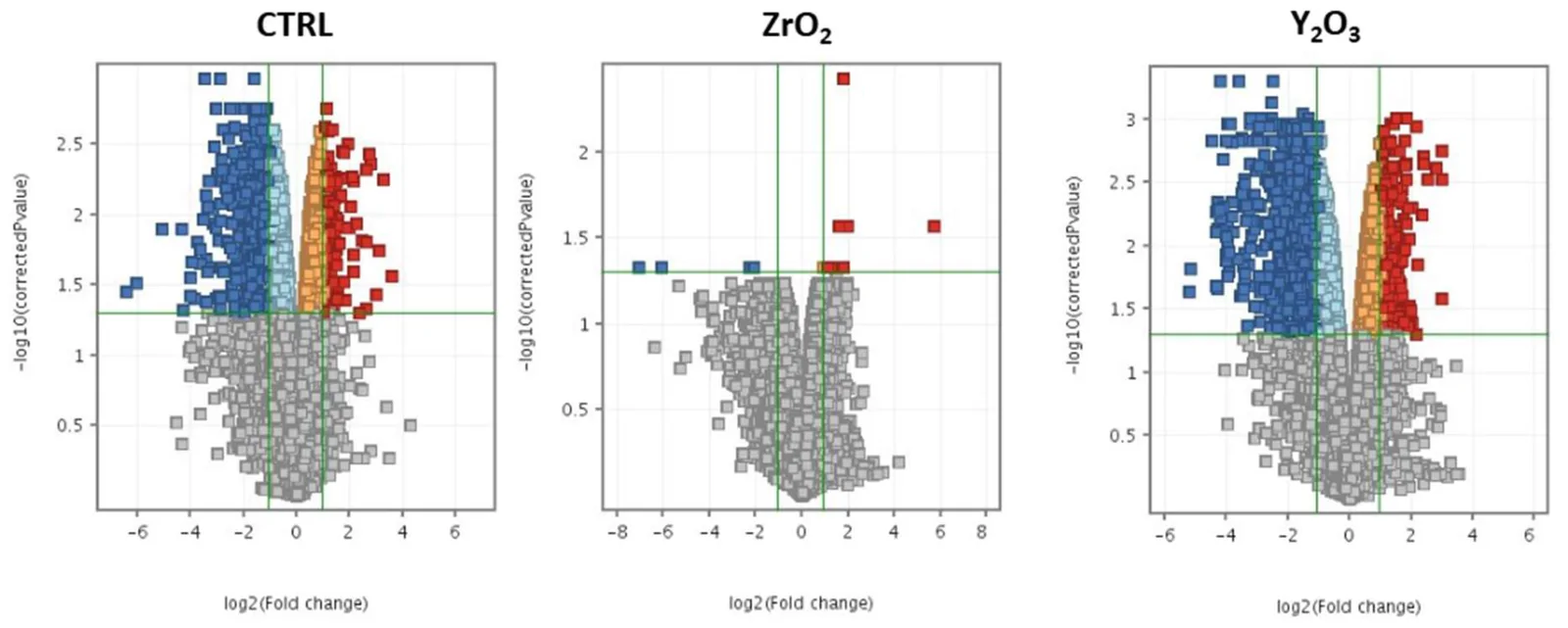
Volcano plots showing the number of transcripts rejected after taking into account a fold change (FC) ≥ 2 (shaded).

**Figure 19:**
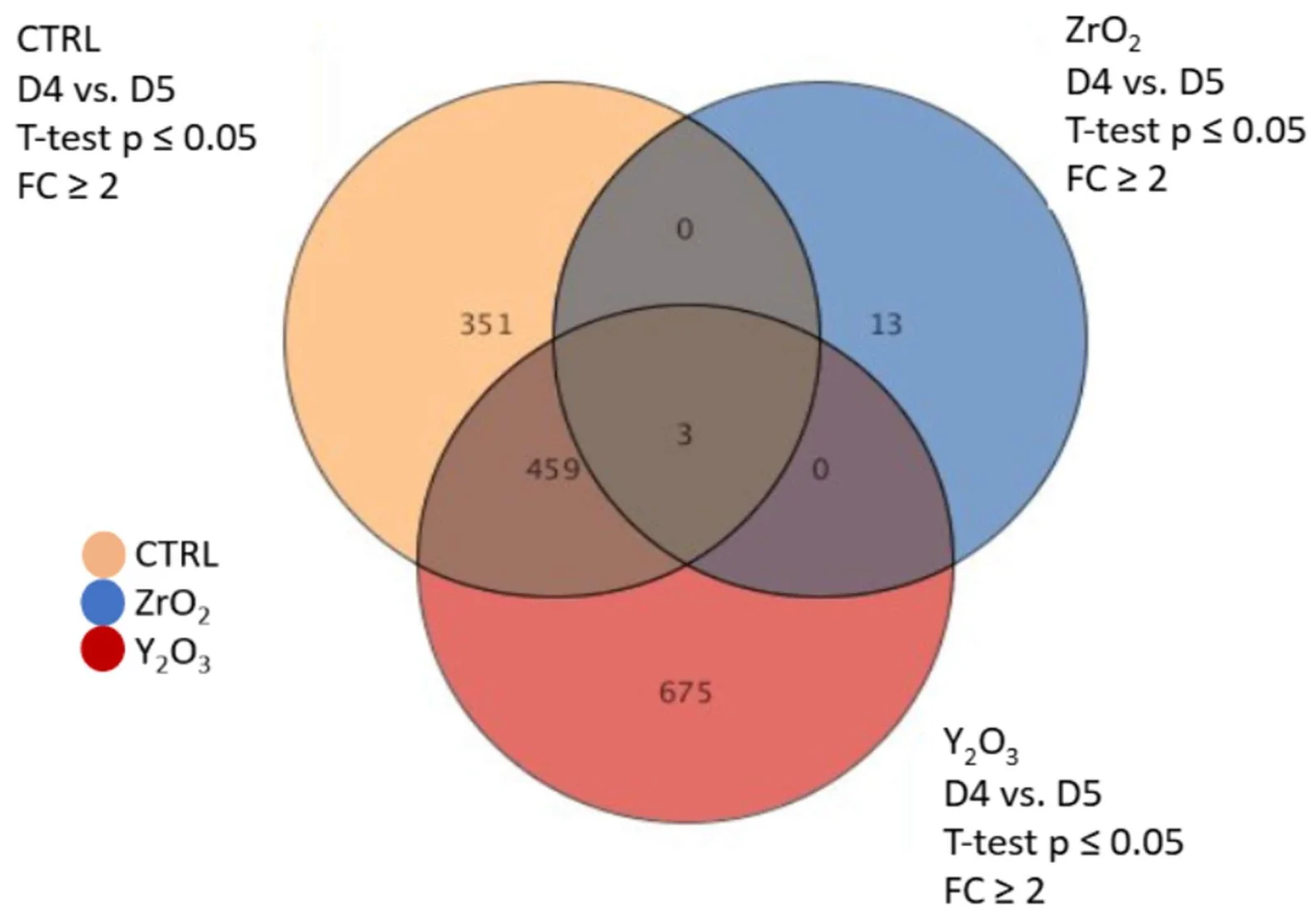
Total number of transcripts showing statistically significant changes in expression (moderated *t*-test, *p* ≤ 0.05, FDR < 0.05), after considering changes only in transcripts whose fold change (FC) ≥ 2.

To check the impact of ZrO_2_ and Y_2_O_3_ NPs at the genomic level, microarray analysis was performed with the results shown as volcano plots (Figure 18) and Venn diagrams (Figure 19).

In the CTRL group, numerous transcripts showed significant changes in expression between day four and day five, reflecting developmental changes occurring during this period. A similar pattern was observed after Y_2_O_3_ NP administration. In contrast, after ZrO_2_ NP administration, only a few differentially expressed transcripts were detected between days 4 and 5, suggesting impact of ZrO_2_ NPs resulting in the weaker transcriptomic response (Figure 18).

Comparing gene expression between the 4th and 5th day of incubation in the CTRL group, the change was observed for 4304 transcripts, 17 transcripts following ZrO_2_ NP administration, and 5373 transcripts following Y_2_O_3_ NP administration. Among them, after taking into account those with a fold change (FC) of two or more, 351 transcripts were specific to the CTRL group, only 13 transcripts were specific to ZrO_2_, and 675 transcripts were specific to Y_2_O_3_ NPs (Figure 19).

In the next step of the analysis, transcripts were selected that were assigned to specific genes and then, using the Reactome pathway database, we investigated which biological pathways they were associated with. Following ZrO_2_ NP administration, only seven genes were identified and assigned to biological pathways. These changes were mainly related to signal transduction and metabolism. In contrast, after Y_2_O_3_ NP administration, 555 genes were assigned to biological pathways, demonstrating a markedly broader transcriptomic response. The main changes occurred in genes related to signal transduction, metabolism, developmental biology, and extracellular matrix organisation.

**Figure 20:**
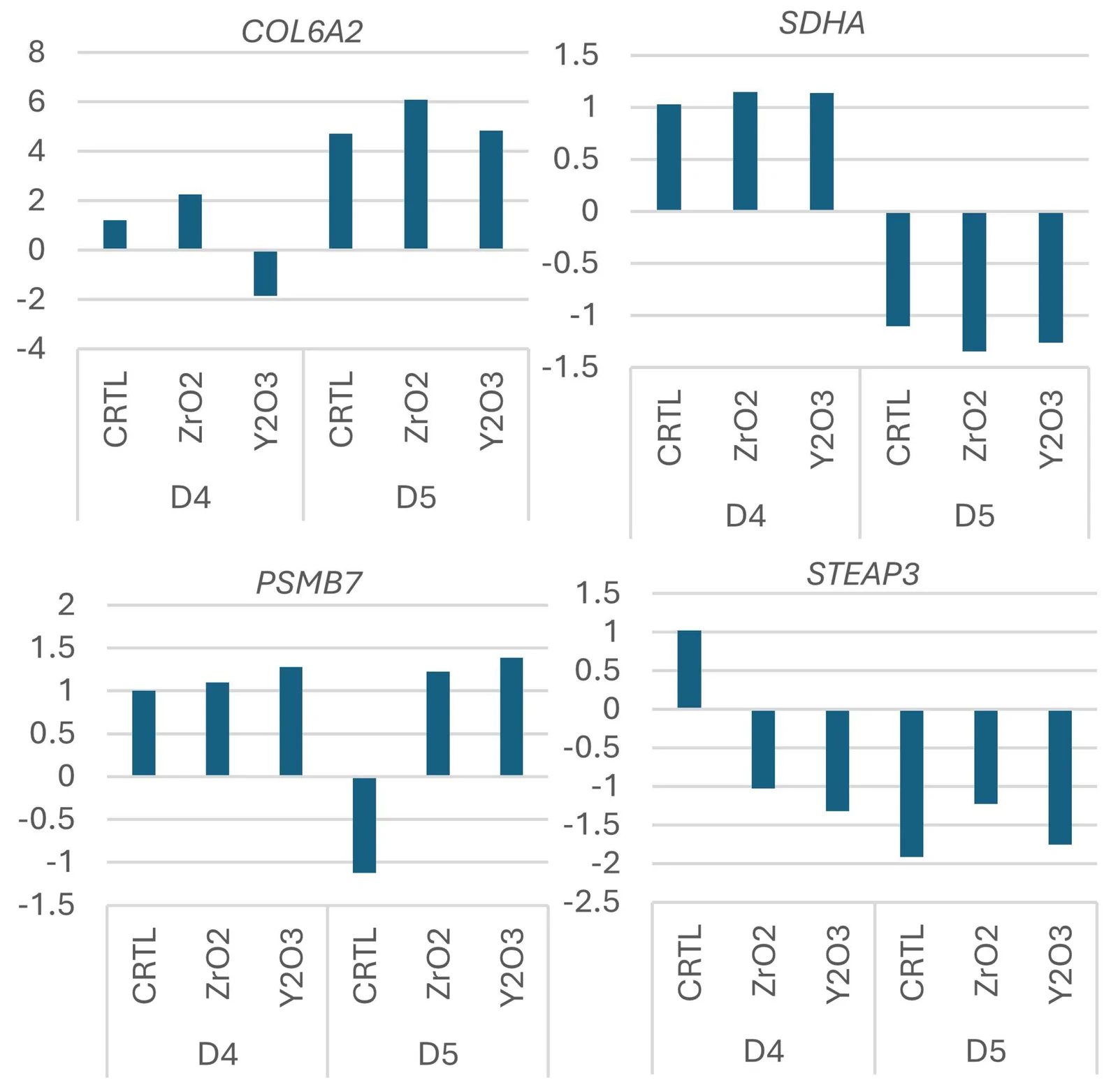
Relative quantification of *COL6A2*, *SDHA*, *PSMB7*, *STEAP3* gene expression. No statistical significance vs. CTRL was observed.

To validate the microarray results, real-time PCR analysis was performed for four selected genes: *COL6A2*, *SDHA*, *PSMB7*, and *STEAP3*. These genes were chosen for comparison with our previous study by Dominik et al., in which biodegradable ZnO NPs were investigated. A statistically significant drop in expression was observed only for *SDHA* on day five vs. CTRL following the administration of ZrO_2_ and Y_2_O_3_ NPs (*p* < 0.001) (Figure 20).

## 4. Discussion

### 4.1. Physicochemical Properties

In the case of Y_2_O_3_: Eu NPs, the complete transformation into the crystalline Y_2_O_3_ phase during calcination confirms the effectiveness of the additional thermal treatment applied after the microwave-assisted hydrothermal process in promoting crystallisation of the material. The absence of detectable secondary phases is particularly important for subsequent biological investigations, since the physicochemical properties of nanomaterials may strongly influence their interactions with biological systems. The use of phase-pure Y_2_O_3_:Eu NPs therefore provides a well-defined material for biological evaluation, reducing the possibility that the observed biological responses are influenced by residual precursor phases or crystalline impurities.

For ZrO_2_:TbY NPs, both tetragonal and monoclinic zirconia phases were detected after calcination. The presence of the tetragonal phase is consistent with partial stabilisation of this high-temperature phase by Y^3+^ ions. Substitution of Zr^4+^ by Y^3+^ is known to promote tetragonal-phase stabilisation through the formation of oxygen vacancies for charge compensation. The pronounced tetragonal (101) reflection at 2Ɵ ≈ 30.2° confirms the substantial contribution of the tetragonal phase to the overall crystal structure. The presence of Tb^3+^ co-dopant ions may additionally affect the local defect structure of ZrO_2_ NPs.

The Y_2_O_3_:Eu NPs retained their elongated morphology after calcination, indicating that the thermal treatment did not result in complete sintering or a substantial loss of the initial particle shape. The rod-like morphology and the broader distribution of particle lengths compared with diameters indicate anisotropic growth, with an aspect ratio of approximately 3.

In contrast, the ZrO_2_:TbY NPs exhibited a quasi-spherical morphology after calcination. A reduction in the visibility of interparticle boundaries and an increase in the average particle diameter of approximately 10 nm compared with the as-grown material were observed, indicating particle growth during thermal treatment without a pronounced change in overall morphology. Such thermal growth may be relevant for the biological behaviour of NPs, as particle size and morphology can affect cellular uptake and intracellular processing [22,23].

The differences between the particle dimensions determined by SEM and the hydrodynamic diameters measured by DLS are expected, because these techniques provide different information. The hydrodynamic diameter is connected to the diffusion behaviour of dispersed entities in aqueous suspension and is affected by the hydration layer, interactions between particles, and aggregation. These effects are particularly relevant for anisotropic NPs, for which the spherical-particle approximation used in DLS analysis may introduce additional deviations [24,25,26].

The Y_2_O_3_:Eu NPs showed a hydrodynamic diameter of 364 nm and a relatively high PDI of 0.6. The broad distribution may be related to the elongated particle morphology and the variation in particle dimensions observed by SEM. Partial aggregation cannot be excluded, although the zeta potential of 25 ± 1 mV indicates a measurable electrostatic surface charge. This value is within the range generally associated with moderate, rather than strong electrostatic stabilisation of the NP suspension [27].

The Y-stabilised ZrO_2_ NPs with additional content of Tb ions exhibited a larger hydrodynamic diameter of 471 nm but a considerably lower PDI of 0.2. This hydrodynamic size is much larger than the nanoparticle size of approximately 52 nm observed by SEM images and may reflect larger particle assemblies formed during thermal treatment and present in the aqueous dispersion. The relatively low PDI indicates a narrower distribution of the detected dispersed population. However, it is worth noting that the presence of a smaller, non-aggregated fraction of primary particles cannot be excluded. This is because intensity-weighted DLS measurements are inherently biased toward larger scattering entities, which produce a disproportionately strong signal and can therefore mask the contribution of smaller particles present in the same suspension [28].

The two materials showed similar absolute zeta-potential values, although with opposite signs: +25 ± 1 mV for Y_2_O_3_:Eu and −27 ± 1 mV for ZrO_2_:TbY NPs. The opposite surface charge suggests differences in the surface chemistry of the two oxide nanoparticle systems. Overall, the differences in hydrodynamic size and distribution are likely related to the combined effects of particle morphology, anisotropy, thermal treatment, and the resulting aggregation.

Photoluminescence measurements confirmed the optical activity of Y_2_O_3_:Eu NPs. The characteristic sharp emission lines originate from intra–4f transitions of trivalent europium ions, with the dominant emission at approximately 611 nm corresponding to the ^5^D_0_→^7^F_2_ transition. Its higher intensity compared with the ^5^D_0_→^7^F_1_ transition at approximately 590 nm suggests that a significant fraction of Eu^3+^ centres experience a locally noncentrosymmetric environment [29].

The excitation spectrum of Y_2_O_3_:Eu NPs contained narrow Eu^3+^ 4f-4f lines in the 300–500 nm range as well as a much broader and more intense band below approximately 230 nm, consistent with a ligand-to-metal charge transfer (LMCT) transition from oxide ions (O^2−^) to europium(III) ions (Eu^3+^) [20]. The presence of excitation bands in the visible region also indicates the possibility of selective excitation of Eu^3+^ ions using blue light, which may be advantageous for optical imaging applications not requiring deep-tissue penetration, such as in vitro studies or imaging of superficial structures.

Compared with the Y_2_O_3_:Eu sample, the excitation behaviour of the ZrO_2_:TbY sample was markedly different. The PLE spectrum monitored at 543 nm was dominated by a broad excitation band centred in the deep UV region (at approximately 260–270 nm), with no clearly resolved 4f-4f excitation lines of Tb^3+^ ions. This behaviour is consistent with excitation involving the ZrO_2_ host and/or defect-related states followed by energy transfer to Tb^3+^ ions, as reported previously for Y and Tb-containing ZrO_2_ NPs [21]. However, the precise contribution of individual excitation pathways cannot be resolved from the present steady-state measurements alone and would require complementary experiments, such as time-resolved spectroscopy.

### 4.2. Biological Study

Our confocal microscopy results indicate that the tissue distribution of ZrO_2_ NPs shares some similarities with the pattern previously reported for ZnO OVAL NPs in chicken embryos [13]. In that study, ZnO OVAL NP distribution depended on both nanoparticle concentration and incubation day, with a stronger signal observed on day 5 after administration of 10 µg/mL than 100 µg/mL [13]. A weaker signal at higher concentrations may result from increased nanoparticle aggregation, which may limit their early penetration into embryonic tissues [30]. In our study, a similar trend was observed for ZrO_2_ nanoparticles, as the lower concentration was detectable earlier than the higher one.

This developmental relationship is confirmed by studies conducted on pregnant mice, in which ZrO_2_ nanoparticles administered orally crossed the biological barriers of the mother and foetus and accumulated in the foetuses’ brains. Furthermore, Wang et al. demonstrated that this relocation depended on the degree of maturity of the blood–placental and blood–brain barriers [31]. Therefore, the pattern of distribution observed in our model of chicken embryos, which depends on the day of development, may be relevant to changes in tissue accessibility and development. In adult mice, Yang et al. also showed organ-dependent biodistribution of ZrO_2_ NPs after intravenous administration, with the highest Zr levels in the liver and spleen [32]. Although this model differs from avian embryonic exposure, it supports the view that ZrO_2_ NPs can be retained in specific tissues and cellular compartments rather than being uniformly distributed throughout the organism.

Meanwhile, the Y_2_O_3_ nanoparticles showed a different distribution pattern, with a more visible signal at the higher concentration on day 5. On day 7, the signal was stronger at the lower concentration, but on day 10, both concentrations showed comparable signal intensity. The distribution of Y_2_O_3_ NPs showed a less concentration-dependent pattern than ZrO_2_ and ZnO OVAL NPs [13], suggesting that nanoparticle behaviour in embryonic tissues is relevant not only by dose and incubation time, but also by material-specific properties. Research into the distribution of Y_2_O_3_ nanoparticles in embryonic tissues is extremely limited, but in vivo data from a model using adult rats suggest that these NPs may be distributed to various organs [33]. Authors showed that, after 28 days of repeated oral exposure in Wistar rats, Y was detected in multiple organs, including the liver, kidneys, blood, intestine, lungs, spleen, heart and brain. The highest Y levels were found in the liver, whereas the lowest were detected in the brain. The simultaneous detection of Y in renal tissue and its urinary excretion suggests that the kidneys may be involved in the processing or elimination of Y-containing substances [33]. Studies in adult mammalian models indicate that ZrO_2_ NPs may disturb redox homeostasis, although these effects were mainly observed under high-dose exposure conditions. Yang et al. showed that intravenous administration of ZrO_2_ NPs (150 nm) to six- to eight-week-old female mice affected oxidative stress markers measured in serum and liver tissue, as demonstrated by reduced SOD activity and increased MDA levels, particularly at the highest dose of 500 mg/kg [32]. Similarly, Arefian et al. reported changes in antioxidant defence after daily intraperitoneal administration of ZrO_2_ to two-month-old male Wistar rats at concentrations of 25, 50, or 100 ppm for one week. In that study, oxidative stress markers were measured in liver homogenates, and significant changes were mainly observed at the highest concentration, 100 ppm, which decreased SOD activity and increased MDA levels, whereas lower concentrations did not markedly affect antioxidant enzyme activity or lipid peroxidation [34].

In contrast, in our study ZrO_2_ NPs increased SOD activity in chicken embryos, which may indicate early activation of antioxidant defence mechanisms rather than depletion of enzymatic antioxidant capacity. Although MDA levels were elevated at the early stage of development, they gradually decreased over time and approached control values, suggesting that ZrO_2_ NPs did not induce long-term lipid peroxidation in chicken embryos. A comparison with our previous study using ZnO NPs suggests that NP morphology may not be the only factor influencing the redox response. Oval ZnO NPs increased SOD activity on day 5 at both concentrations tested, showing some similarity to the early activation of antioxidant mechanisms observed after administration of ZrO_2_ nanoparticles [13]. However, the further oxidative changes varied depending on the type of material. Following administration of ZnO OVAL, MDA concentrations showed an increase in the later stages of incubation, whilst in this experiment, the increase in MDA following administration of ZrO_2_ NPs occurred in the earlier stages and later became similar to the values observed in the CTRL. Differences were also observed in protein oxidation, as changes in CP content following ZnO OVAL NP administration occurred only at selected time points, whereas ZrO_2_ NPs increased CP levels at almost all analysed time points. These results indicate that, despite some similarities in the early antioxidant response, the redox changes were different for ZrO_2_ and ZnO nanoparticles and may also depend on the physicochemical properties of these materials. This different response may be due to the process of embryonic development, in which redox regulation increases very dynamically during organogenesis, and antioxidant systems may be rapidly activated in response to stressors. Jiao et al. showed that okadaic acid exposure induced oxidative stress in chicken embryos at day 1.5 of incubation, as evidenced by increased ROS levels and SOD activity, whereas MDA levels remained unchanged [35]. This suggests that increased SOD activity in early chick embryos does not necessarily coincide with sustained lipid peroxidation. Barbasz and Dyba showed that ZrO_2_ NPs in human immune cell models after 48 h of exposure lead to increased MDA level in HUT-78 cells, whereas no statistically significant differences were observed in HL-60 cells [36]. Driessen et al. investigated ZrO_2_ NPs in NRK-52E rat kidney epithelial cells. ZrO_2_ NPs used by authors were very small nanomaterials, with a size of 9 nm, whereas the ZrO_2_ NPs used in our study were larger, with an average size of 52 nm [37]. Thus, the size of NPs used in our study influenced their behaviour in the biological system, including cellular interactions, cell membrane trafficking, and following oxidative responses. In contrast to the results of the study by Driessen et al. [37], a statistically significant increase in the levels of carbonylated proteins was observed in chicken embryos following administration of ZrO_2_ NPs at almost all analysed time points. However, the increase was relatively small, indicating a measurable but limited level of oxidative protein modifications. This discrepancy may be mainly due to differences in nanoparticle size and biological model, as Driessen et al. [37] used small ZrO_2_ nanomaterials in an epithelial cell line, whereas our study employed larger ZrO_2_ NPs in a whole chicken embryo model.

The results of Asadpour et al. showed that, after 24 h of exposure to ZrO_2_ NPs, N2a cell viability decreased to approximately 50% at a concentration of 200 µg/mL, a mouse neuroblastoma cell line, whilst for PC12 cells, a neuronal model derived from a rat adrenal tumour, a similar reduction in viability was observed at approximately 1000 µg/mL [38]. In contrast, our results on CEF cells showed significantly greater sensitivity to ZrO_2_ NPs, with cell viability decreasing to approximately 50% at 1 µg/mL after 24 h of exposure (Figure 9B). This difference indicates that primary embryonic cells may be more sensitive to ZrO_2_ NPs induced cytotoxicity than immortalised neuronal cell models, highlighting the importance of using diverse experimental models, including systems that are not based solely on established cell lines. Despite the high sensitivity of CEF cells observed in vitro, the administration of ZrO_2_ NPs at concentrations of 10 and 100 µg/mL to fertilised chicken eggs did not affect embryo growth, development and mortality when compared with embryos from the control group. This suggests that results obtained in isolated primary cells should not be directly applied to whole embryos, where the final biological outcome may be influenced by the distribution of NPs, biological barriers, tissue-specific reactions, and compensatory mechanisms associated with development.

Emad et al. [39] reported that Y_2_O_3_ NPs reduced the viability of human triple-negative breast cancer MDA-MB-231 cells after 48 h of exposure, with an IC_50_ value of 74.4 µg/mL, whereas normal human dermal fibroblasts (HDF) were less sensitive, with an IC_50_ value estimated at 230.87 µg/mL. However, Akhtar et al. [40] did not observe cytotoxicity in human MCF-7 breast carcinoma cells or HT-1080 fibrosarcoma-derived fibroblast-like cells up to 200 µg/mL after 24 h of exposure. In our study, CEF viability decreased to approximately 10% already at the lowest tested concentration of Y_2_O_3_ NPs (0.1 µg/mL) after 24 h of exposure. This indicates that, like for ZrO_2_ NPs, CEFs were more sensitive to Y_2_O_3_ nanoparticle-induced cytotoxicity than the human cell lines. This difference once again shows the higher sensitivity of primary embryonic cells compared to established human cell lines. With regard to oxidative stress, Emad et al. [39] showed that Y_2_O_3_ NPs affected redox-related parameters in human MDA-MB-231 cells. After 48 h of exposure, Y_2_O_3_ NPs increased MDA levels by 1.3-fold and CAT activity by 1.4-fold, indicating enhanced lipid peroxidation accompanied by activation of antioxidant defence. Y_2_O_3_ NPs also modified oxidative stress-related parameters in chicken embryos, as demonstrated by increased SOD activity and higher MDA content observed especially at later stages of the experiment, on days 8 and 9. Despite differences in NPs surface charge, as the particles used by Emad et al. had a negative zeta potential, whereas those used in our study were positively charged [39]. These results suggest that the biological response to Y_2_O_3_ NPs may involve both oxidative damage and compensatory activation of antioxidant defence mechanisms. Similarly, Mohamed et al. showed that Y_2_O_3_ NPs induced oxidative stress in human epidermoid skin cancer A-431 cells after 72 h of exposure. They showed an increase in ROS generation and MDA levels, while the activities of SOD and CAT were significantly decreased [41].

Panyala et al. reported that oral administration Y_2_O_3_ NPs disturbed redox homeostasis in adult female Wistar rats, only at the highest tested dose of 1000 mg/kg body weight, but lower doses of 250 and 500 mg/kg did not cause significant changes in oxidative stress markers. In that study, oxidative stress markers were measured in serum, liver and kidney after 24 and 72 h, and increased MDA levels were accompanied by decreased SOD activity [42]. In our study, Y_2_O_3_ NPs increased SOD activity in chicken embryos, particularly during later stages of development while MDA levels changed during development and were higher at later stages of experiments. The response of chicken embryos to Y_2_O_3_ NPs may be associated with the activation of antioxidant defence mechanisms and development-dependent oxidative changes. Partially similar changes were observed in our previous study on LONG ZnO NPs, in which the MDA content also increased during the later stages of incubation [13]. Akhtar et al. exposed MCF-7 and HT-1080 cells to different concentrations (50, 100 and 200 µg/mL) of Y_2_O_3_ NPs and showed that, regardless of the applied concentration these NPs did not significantly affect the oxidative stress-related parameters including ROS production and level of MDA [40].

In addition to lipid peroxidation, Y_2_O_3_ NP administration was also associated with oxidative modification of proteins. Carbonylated protein content was higher than in the CTRL samples at almost all analysed time points, indicating that protein oxidation was a consistent component of the embryonic response to Y_2_O_3_ NPs. Together with the increased MDA content observed especially at later stages of the experiment, these results suggest that Y_2_O_3_ NPs induced oxidative damage affecting both lipids and proteins. At the same time, the increased SOD activity may reflect subsequent activation of antioxidant defence mechanisms. In our previous study, LONG ZnO NPs also increased the CP content, mainly in the later stages of incubation. However, following administration of Y_2_O_3_ nanoparticles, CP concentrations were elevated at a higher number of time points than after exposure to ZnO LONG nanoparticles, suggesting differences in the pathway of protein oxidation between these two types of nanoparticles [13].

Alhusayni et al. investigated the effects of Y_2_O_3_ NPs on mouse in vitro fertilisation and preimplantation embryo development. In their study, Y_2_O_3_ NPs were added to fertilisation and embryo culture media at concentrations of 25 and 50 µg/mL. The authors reported that Y_2_O_3_ NPs did not affect the cleavage rate, but in the higher concentration of 50 µg/mL reduced blastocyst development compared with the control and 25 µg/mL groups [11]. The reduction in blastocyst development at the higher Y_2_O_3_ nanoparticle concentration suggests that later embryonic stages may be more sensitive than early cleavage, showing the need for further developmental studies, including tissue differentiation and organ development.

The limited transcriptomic response observed after administration of ZrO_2_ NPs is consistent with data from proteomic studies, suggesting that ZrO_2_ NPs may induce weaker molecular changes than nanoparticles based on other metal oxides, such as ZnO. Aude-Garcia et al. compared the proteomic response of J774 macrophages to ZnO and ZrO_2_ NPs and demonstrated that the most pronounced nanoparticle-specific effects were mainly associated with ZnO NPs, including changes in mitochondrial proteins, signalling molecules and proteins associated with DNA damage [43]. Although this study was conducted at the proteomic rather than the transcriptomic level, it may support the interpretation that ZrO_2_ NPs may induce a more limited molecular response. In the present study, this limited response was demonstrated by the small number of transcripts showing changes in expression after ZrO_2_ NP administration and by the fact that only a few genes were assigned to biological pathways, mainly those related to signal transduction and metabolism. The pathways affected by ZrO_2_ NPs in the present study partly overlapped with those previously reported for ZnO NPs in chicken embryos, particularly signal transduction and metabolism [13]. However, in contrast to the broad transcriptomic response induced by ZnO NPs, ZrO_2_ NPs affected only a very limited number of transcripts and genes. The pathways affected by ZrO_2_ NPs in this study partially overlapped with those affected by ZnO NPs, in terms of signal transduction and energy metabolism [13]. However, the range of the transcriptomic response was significantly different. In our previous study, 1487 genes were identified following administration of OVAL ZnO NPs and 548 following administration of LONG ZnO, whilst only 7 genes showed changes in expression following administration of ZrO_2_ NPs. Therefore, despite some overlap in the pathways affected by the NPs, the transcriptomic response to ZrO_2_ NPs was significantly more limited than that observed for both forms of ZnO NPs.

A similarly weak molecular response to ZrO_2_ NPs was reported by Triboulet et al., who used zirconium oxide nanoparticles as a control material in a proteomic study of mouse macrophages exposed to copper-based nanoparticles. In that study, treatment with 10 µg/mL ZrO_2_ NPs for 24 h induced very few detectable proteomic changes [44]. The question of whether weaker transcriptomic response for ZrO_2_ NPs, when compared to control, was a result of earlier higher genomic response or a delay in gene transcription remains to be evaluated by further studies. However, the fact that no difference in mortality nor embryonic growth was observed, the former seems more viable.

The broad transcriptomic response observed following administration of Y_2_O_3_ NPs was similar to that observed after administration of LONG ZnO NPs. The administration of Y_2_O_3_ nanoparticles resulted in changes in the expression of 555 genes, while our previous study [13] identified 548 genes following the administration of LONG ZnO NPs. Additionally, both types of nanoparticles influenced pathways related to signal transduction and energy metabolism [13,45]. Moriyama, who analysed the effects of Y_2_O_3_ NPs in *Saccharomyces cerevisiae* using DNA microarrays, showed that exposure to Y_2_O_3_ NPs induced extensive changes in gene expression, including up-regulation of genes associated with oxidoreductase activity, stress response, the respiratory chain and proteasome function [45]. Although this study was performed in yeast and therefore cannot be directly compared with chicken embryos, it supports the view that Y_2_O_3_ NPs can strongly affect redox regulation and metabolic pathways at the transcriptomic level in the eucaryotic cells. Both Y_2_O_3_ NPs and ZnO NPs [13] affected pathways related to signal transduction and metabolism. This may indicate that the transcriptomic response in chicken embryos does not depend solely on the biodegradability of the NPs, but is strongly dependant on the NPs shape.

In the present study, qPCR analysis showed that the most evident expression change after administration of non-biodegradable ZrO_2_ and Y_2_O_3_ NPs was observed for *SDHA*. A significant decrease in *SDHA* expression was detected on day 5 after administration of both nanoparticle types. *SDHA* encodes succinate dehydrogenase flavoprotein subunit A, a catalytic subunit of mitochondrial complex II, which links the tricarboxylic acid cycle with the mitochondrial electron transport chain [46]. Therefore, the decreased expression of this gene may suggest that non-biodegradable NPs affect mitochondrial energy metabolism during chicken embryo development. This response was different from that observed after administration of ZnO NPs in our previous study in which the same set of genes was analysed by qPCR in chicken embryos exposed to biodegradable ZnO NPs [13]. In that study, the response was mainly associated with *COL6A2* expression, whereas *SDHA* expression was not significantly affected. This suggests that non-biodegradable ZrO_2_ and Y_2_O_3_ NPs may induce a different molecular response than ZnO NPs, with a stronger impact on genes related to mitochondrial metabolism. At the transcriptomic level, Y_2_O_3_ NPs strongly affected pathways related to metabolism and signal transduction, whereas ZrO_2_ NPs induced a much more limited transcriptional response. At the biochemical level, both nanoparticle types modified redox-related parameters, including SOD activity, MDA content and CP levels. The qPCR results support the view that mitochondrial function, energy metabolism and oxidative stress are closely involved in the embryonic response to non-biodegradable ZrO_2_ and Y_2_O_3_ NPs.

## 5. Conclusions

Comprehensive physicochemical characterisation of non-biodegradable Y_2_O_3_ and ZrO_2_ nanoparticles doped with rare-earth ions showed different fluorescent properties. Distinct luminescence responses were observed, with intense Eu^3+^ emission in Y_2_O_3_ and characteristic green Tb^3+^ emission in ZrO_2_. The shape of studied nanoparticles and their hydrodynamic diameters resemble those of earlier-studied ZnO NPs. Presence of both ZrO_2_ and Y_2_O_3_ NPs was confirmed in the tissues of chicken embryos.

Both nondegradable ZrO_2_ and Y_2_O_3_ nanoparticles induced prominent changes in oxidative stress markers in the chicken embryonic tissues, when compared with CTRL and earlier ZnO study [13]. Whole-genome expression analysis showed that oval ZrO_2_ nanoparticles, induced the severe reduction in gene expression, when compared to both CTRL and our earlier results for oval ZnO nanoparticles. This hitherto undocumented observation prompts a more pronounced investigation on the effect of ZrO_2_ NPs on developing embryo and raises a significant question about their, assumed, biosafety. On the other hand, elongated Y_2_O_3_ nanoparticles induce expression changes similar to earlier-observed for ZnO NPs, mostly affecting signal transduction, intracellular trafficking and intermediate metabolism. This early-stage observation indicates previously undocumented effect of the nanoparticles; however, the full impact of the described whole genome expression analysis will be known following full Reactome analyses and the evaluation of changes at the key protein checkpoints.

## Figures and Tables

**Table 1:** Hydrodynamic diameter and polydispersity index obtained from dynamic light scattering (DLS) together with the zeta potential measured by electrophoretic light scattering (ELS) for the Y_2_O_3_:Eu and Y-stabilised ZrO_2_:Tb NPs.

Sample	Hydrodynamic Size d (nm)	Polydispersity Index	Zeta Potential Analysis ζ(mV)
Y_2_O_3_: Eu	364	0.6	25 ± 1
ZrO_2_: 0.5% mol. Tb. 3% mol. Y	471	0.2	−27 ± 1

## Data Availability

The microarray data presented in this study are openly available in the Gene Expression Omnibus data repository under the accession number: GSE326080. Original, unprocessed confocal microphotographs are attached as the Supplementary Materials to this article.

## Supplementary Materials

The following supporting information can be downloaded at: https://www.mdpi.com/article/10.3390/nano16211347/s1, Figure S1: Confocal microscopy image of chicken embryo tissues following administration of Y_2_O_3_ NPs at concentration 10 μg/mL at day 5 of incubation. Lens magnification 20×. Blue fluorescence corresponds to nuclei stained with Hoechest 33342, while red fluorescence indicates Y_2_O_3_ NPs; Figure S2: Confocal microscopy image of chicken embryo tissues following administration of Y_2_O_3_ NPs at concentration 10 μg/mL at day 5 of incubation. Lens magnification 20×. Blue fluorescence corresponds to nuclei stained with Hoechest 33342, while red fluorescence indicates Y_2_O_3_ NPs; Figure S3: Confocal microscopy image of chicken embryo tissues following administration of Y_2_O_3_ NPs at concentration 10 μg/mL at day 7 of incubation. Lens magnification 20×. Blue fluorescence corresponds to nuclei stained with Hoechest 33342, while red fluorescence indicates Y_2_O_3_ NPs; Figure S4: Confocal microscopy image of chicken embryo tissues following administration of Y_2_O_3_ NPs at concentration 10 μg/mL at day 7 of incubation. Lens magnification 20×. Blue fluorescence corresponds to nuclei stained with Hoechest 33342, while red fluorescence indicates Y_2_O_3_ NPs; Figure S5: Confocal microscopy image of chicken embryo tissues following administration of Y_2_O_3_ NPs at concentration 10 μg/mL at day 10 of incubation. Lens magnification 20×. Blue fluorescence corresponds to nuclei stained with Hoechest 33342, while red fluorescence indicates Y_2_O_3_ NPs; Figure S6: Confocal microscopy image of chicken embryo tissues following administration of Y_2_O_3_ NPs at concentration 10 μg/mL at day 10 of incubation. Lens magnification 20×. Blue fluorescence corresponds to nuclei stained with Hoechest 33342, while red fluorescence indicates Y_2_O_3_ NPs; Figure S7: Confocal microscopy image of chicken embryo tissues following administration of Y_2_O_3_ NPs at concentration 100 μg/mL at day 5 of incubation. Lens magnification 20×. Blue fluorescence corresponds to nuclei stained with Hoechest 33342, while red fluorescence indicates Y_2_O_3_ NPs; Figure S8: Confocal microscopy image of chicken embryo tissues following administration of Y_2_O_3_ NPs at concentration 100 μg/mL at day 5 of incubation. Lens magnification 20×. Blue fluorescence corresponds to nuclei stained with Hoechest 33342, while red fluorescence indicates Y_2_O_3_ NPs; Figure S9: Confocal microscopy image of chicken embryo tissues following administration of Y_2_O_3_ NPs at concentration 100 μg/mL at day 7 of incubation. Lens magnification 20×. Blue fluorescence corresponds to nuclei stained with Hoechest 33342, while red fluorescence indicates Y_2_O_3_ NPs; Figure S10: Confocal microscopy image of chicken embryo tissues following administration of Y2O3 NPs at concentration 100 μg/mL at day 7 of incubation. Lens magnification 20×. Blue fluorescence corresponds to nuclei stained with Hoechest 33342, while red fluorescence indicates Y_2_O_3_ NPs; Figure S11: Confocal microscopy image of chicken embryo tissues following administration of Y_2_O_3_ NPs at concentration 100 μg/mL at day 10 of incubation. Lens magnification 20×. Blue fluorescence corresponds to nuclei stained with Hoechest 33342, while red fluorescence indicates Y_2_O_3_ NPs; Figure S12: Confocal microscopy image of chicken embryo tissues following administration of Y_2_O_3_ NPs at concentration 100 μg/mL at day 10 of incubation. Lens magnification 20×. Blue fluorescence corresponds to nuclei stained with Hoechest 33342, while red fluorescence indicates Y_2_O_3_ NPs; Figure S13: Confocal microscopy image of chicken embryo tissues following administration of ZrO_2_ NPs at concentration 10 μg/mL at day 5 of incubation. Lens magnification 20×. Blue fluorescence corresponds to nuclei stained with Hoechest 33342, while green fluorescence indicates ZrO_2_ NPs; Figure S14: Confocal microscopy image of chicken embryo tissues following administration of ZrO_2_ NPs at concentration 10 μg/mL at day 5 of incubation. Lens magnification 20×. Blue fluorescence corresponds to nuclei stained with Hoechest 33342, while green fluorescence indicates ZrO_2_ NPs; Figure S15: Confocal microscopy image of chicken embryo tissues following administration of ZrO_2_ NPs at concentration 10 μg/mL at day 7 of incubation. Lens magnification 20×. Blue fluorescence corresponds to nuclei stained with Hoechest 33342, while green fluorescence indicates ZrO_2_ NPs; Figure S16: Confocal microscopy image of chicken embryo tissues following administration of ZrO_2_ NPs at concentration 10 μg/mL at day 7 of incubation. Lens magnification 20×. Blue fluorescence corresponds to nuclei stained with Hoechest 33342, while green fluorescence indicates ZrO_2_ NPs; Figure S17: Confocal microscopy image of chicken embryo tissues following administration of ZrO_2_ NPs at concentration 10 μg/mL at day 10 of incubation. Lens magnification 20×. Blue fluorescence corresponds to nuclei stained with Hoechest 33342, while green fluorescence indicates ZrO_2_ NPs; Figure S18: Confocal microscopy image of chicken embryo tissues following administration of ZrO_2_ NPs at concentration 10 μg/mL at day 10 of incubation. Lens magnification 20×. Blue fluorescence corresponds to nuclei stained with Hoechest 33342, while green fluorescence indicates ZrO_2_ NPs; Figure S19: Confocal microscopy image of chicken embryo tissues following administration of ZrO_2_ NPs at concentration 100 μg/mL at day 5 of incubation. Lens magnification 20×. Blue fluorescence corresponds to nuclei stained with Hoechest 33342, while green fluorescence indicates ZrO_2_ NPs; Figure S20: Confocal microscopy image of chicken embryo tissues following administration of ZrO_2_ NPs at concentration 100 μg/mL at day 5 of incubation. Lens magnification 20×. Blue fluorescence corresponds to nuclei stained with Hoechest 33342, while green fluorescence indicates ZrO_2_ NPs; Figure S21: Confocal microscopy image of chicken embryo tissues following administration of ZrO_2_ NPs at concentration 100 μg/mL at day 7 of incubation. Lens magnification 20×. Blue fluorescence corresponds to nuclei stained with Hoechest 33342, while green fluorescence indicates ZrO_2_ NPs; Figure S22: Confocal microscopy image of chicken embryo tissues following administration of ZrO_2_ NPs at concentration 100 μg/mL at day 7 of incubation. Lens magnification 20×. Blue fluorescence corresponds to nuclei stained with Hoechest 33342, while green fluorescence indicates ZrO_2_ NPs; Figure S23: Confocal microscopy image of chicken embryo tissues following administration of ZrO_2_ NPs at concentration 100 μg/mL at day 10 of incubation. Lens magnification 20×. Blue fluorescence corresponds to nuclei stained with Hoechest 33342, while green fluorescence indicates ZrO_2_ NPs; Figure S24: Confocal microscopy image of chicken embryo tissues following administration of ZrO_2_ NPs at concentration 100 μg/mL at day 10 of incubation. Lens magnification 20×. Blue fluorescence corresponds to nuclei stained with Hoechest 33342, while green fluorescence indicates ZrO_2_ NPs.

## Abbreviations

The following abbreviations are used in this manuscript:

BHT	butylated hydroxytoluene
CEFs	(primary) chicken embryo fibroblasts
CP	carbonylated proteins
CTRL	control
DLS	dynamic light scattering
DMEM	Dulbecco’s modified eagle medium
DNPH	2,4-dinitrophenylhydrazine
ELS	electrophoretic light scattering
FBS	foetal bovine serum
MDA	malondialdehyde
NBT	nitro blue tetrazolium chloride
NPs	nanoparticles
PDI	polydispersity indices
PL	photoluminescence emission
PLE	photoluminescence excitation
PMSF	phenylmethanesulfonyl fluoride
ROS	reactive oxygen species
SEM	scanning electron microscopy
SOD	superoxide dismutase
TBA	thiobarbituric acid
TCA	trichloroacetic acid
XRD	X-ray Diffraction
