# Supplementary material for: Influence of Non-Biodegradable ZrO2 and Y2O3 Nanoparticles on the Genomic and Redox Status of Chicken Embryo
Source: Nanomaterials (Basel). 2026 Oct 23;16(21):1347. doi: 10.3390/nano16211347 (PMC13764832; doi:10.3390/nano16211347)
Supplement: Supplementary file 1 [file nanomaterials-16-01347-s001.zip › Dominiak et al 2026 supplementary materials.pdf]

Supplementary information:

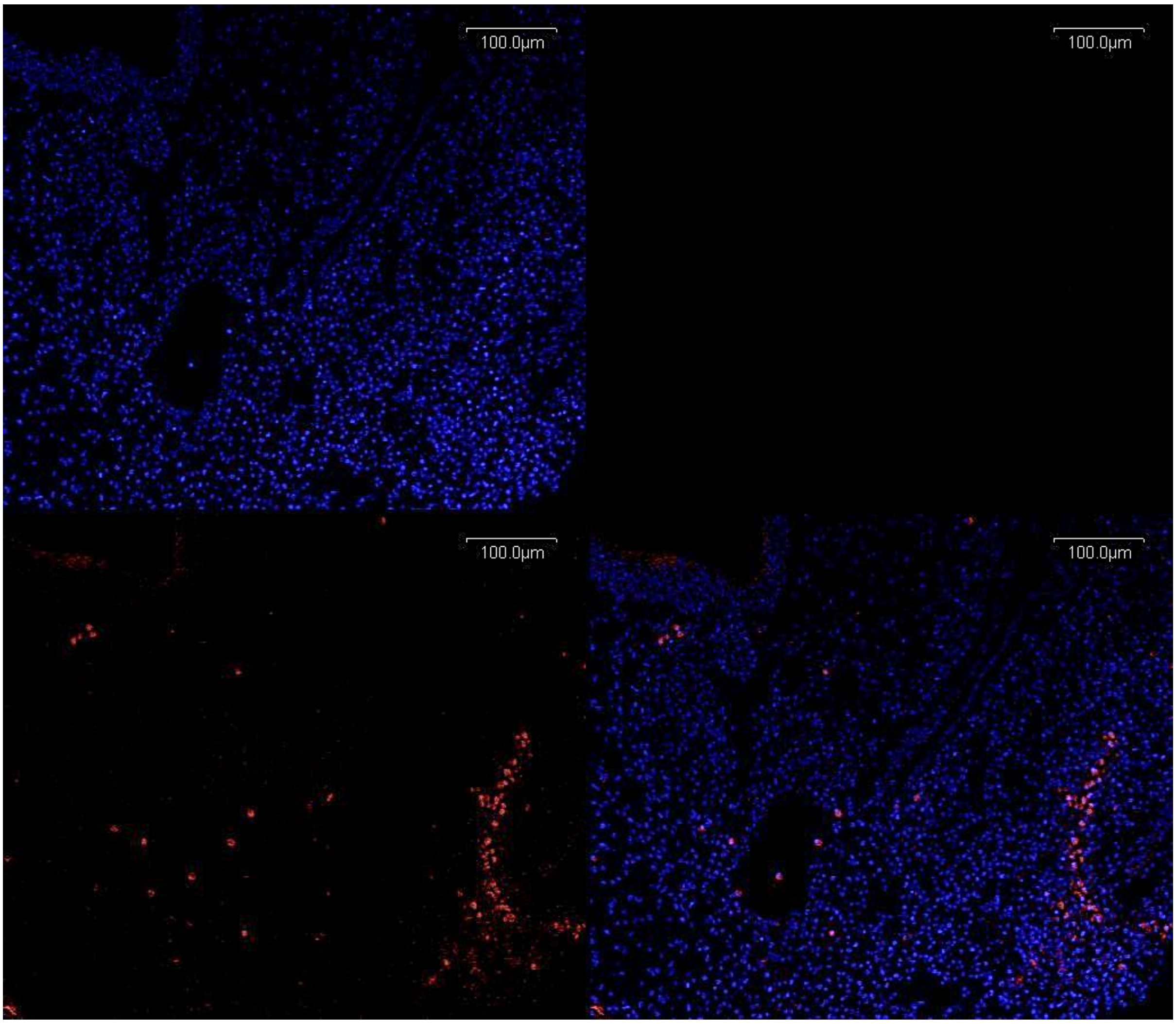

Figure S1. Confocal microscopy image of chicken embryo tissues following administration of Y_2O_3 NPs at concentration 10 μg/ml at day 5 of incubation. Lens magnification 20x. Blue fluorescence corresponds to nuclei stained with Hoechest 33342, while red fluorescence indicates Y_2O_3 NPs.

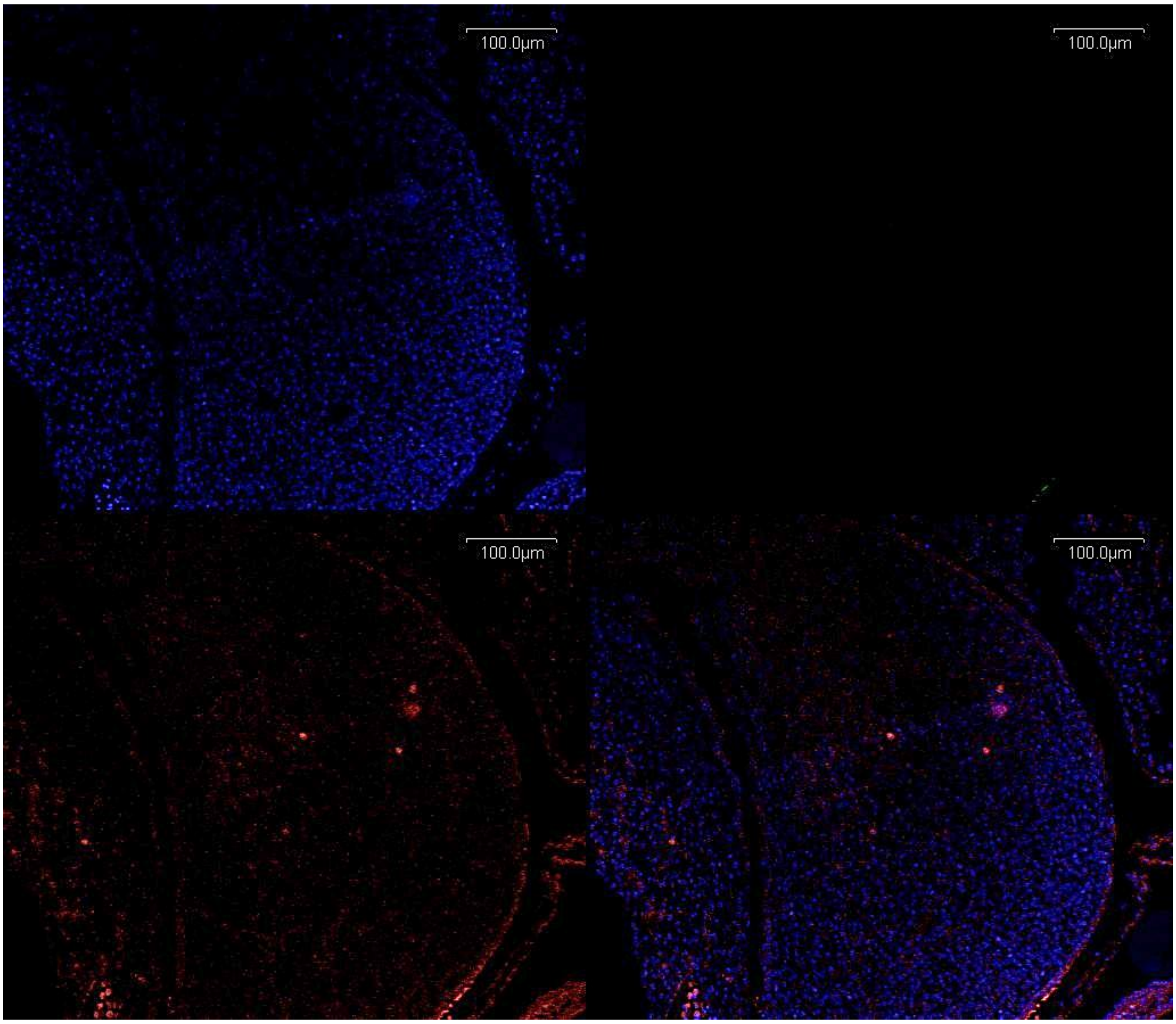

Figure S2. Confocal microscopy image of chicken embryo tissues following administration of Y_2O_3 NPs at concentration 10 μg/ml at day 5 of incubation. Lens magnification 20x. Blue fluorescence corresponds to nuclei stained with Hoechest 33342, while red fluorescence indicates Y_2O_3 NPs.

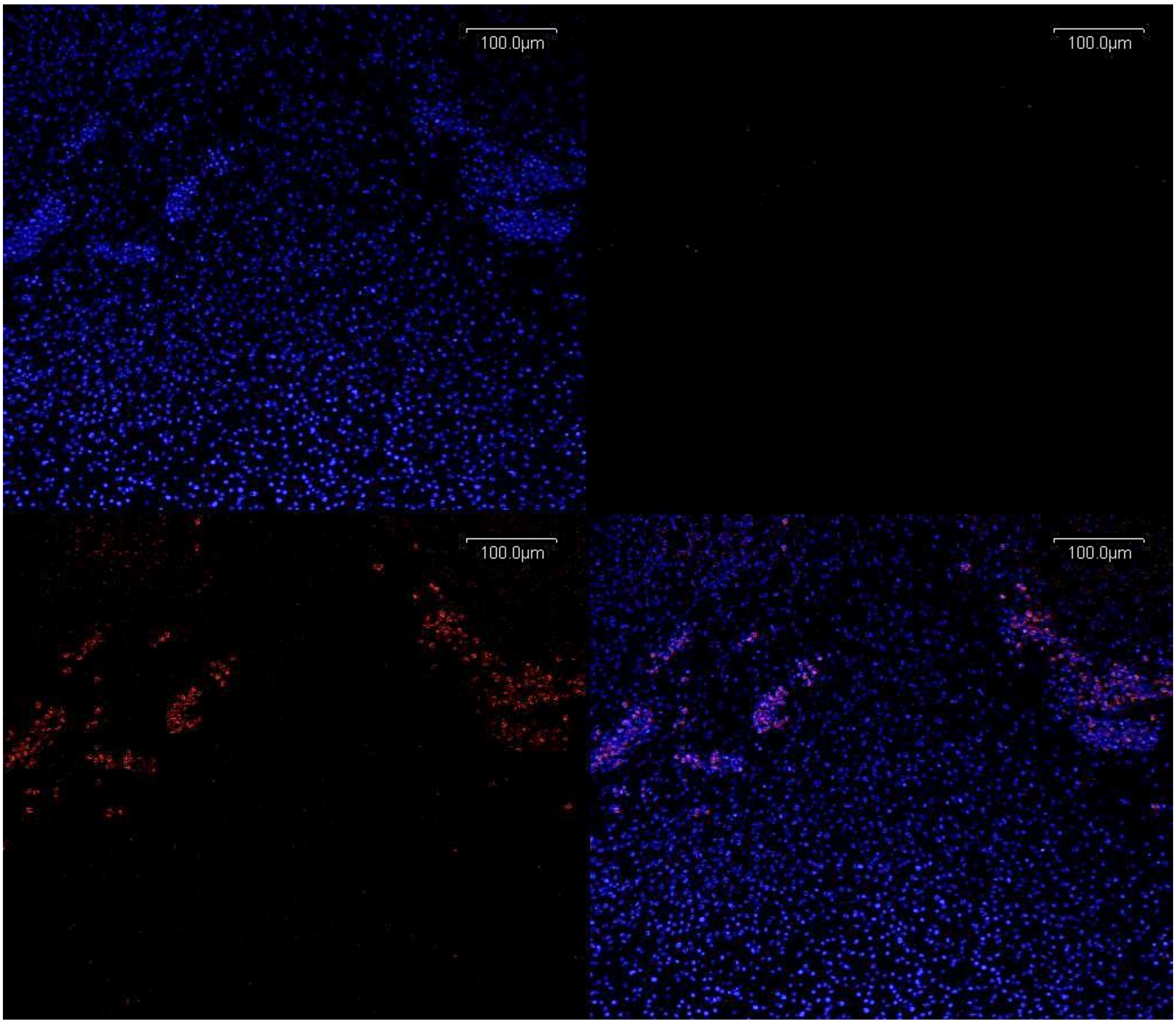

Figure S3. Confocal microscopy image of chicken embryo tissues following administration of Y_2O_3 NPs at concentration 10 μg/ml at day 7 of incubation. Lens magnification 20x. Blue fluorescence corresponds to nuclei stained with Hoechest 33342, while red fluorescence indicates Y_2O_3 NPs.

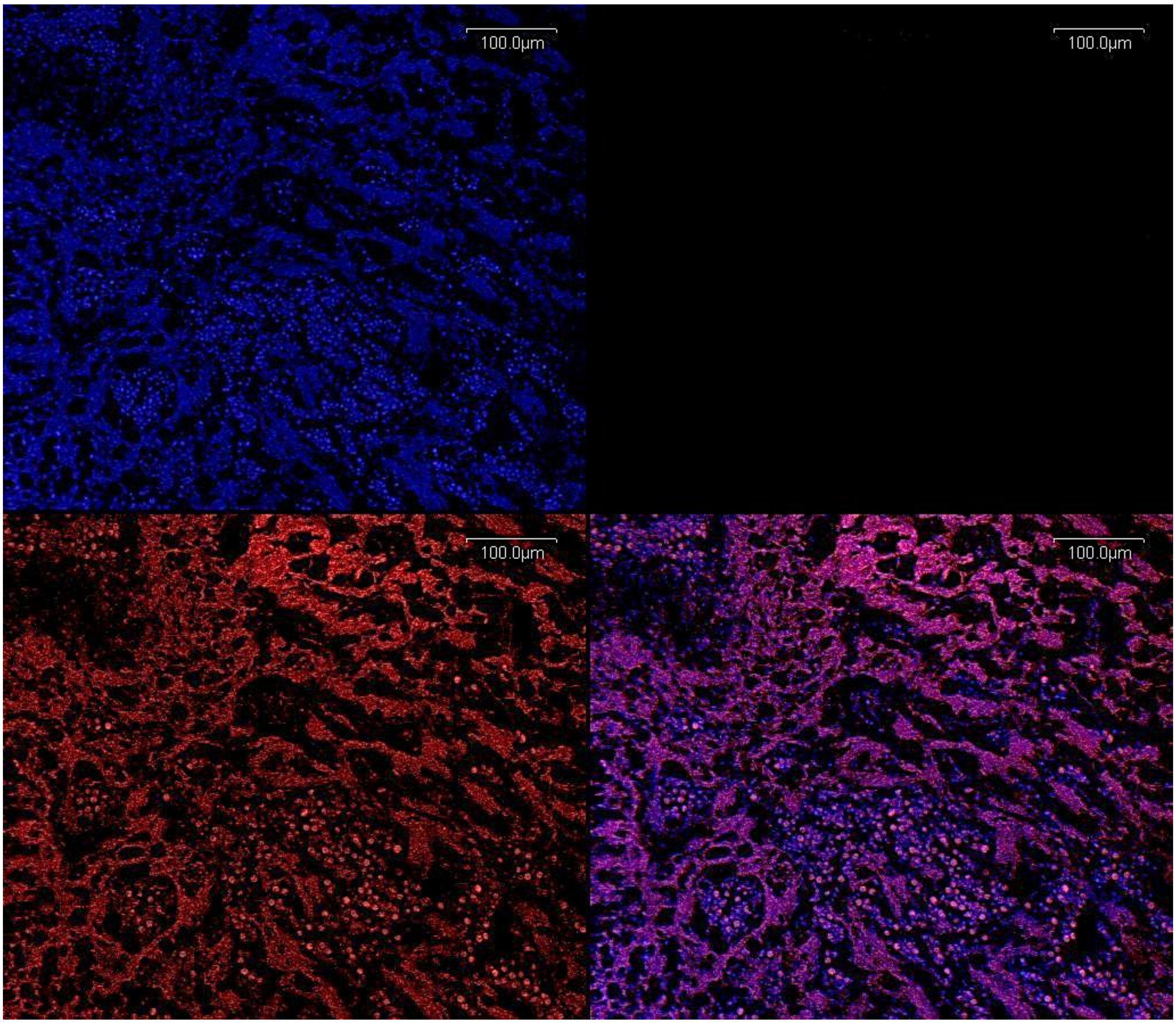

Figure S4. Confocal microscopy image of chicken embryo tissues following administration of Y_2O_3 NPs at concentration 10 μg/ml at day 7 of incubation. Lens magnification 20x. Blue fluorescence corresponds to nuclei stained with Hoechest 33342, while red fluorescence indicates Y_2O_3 NPs.

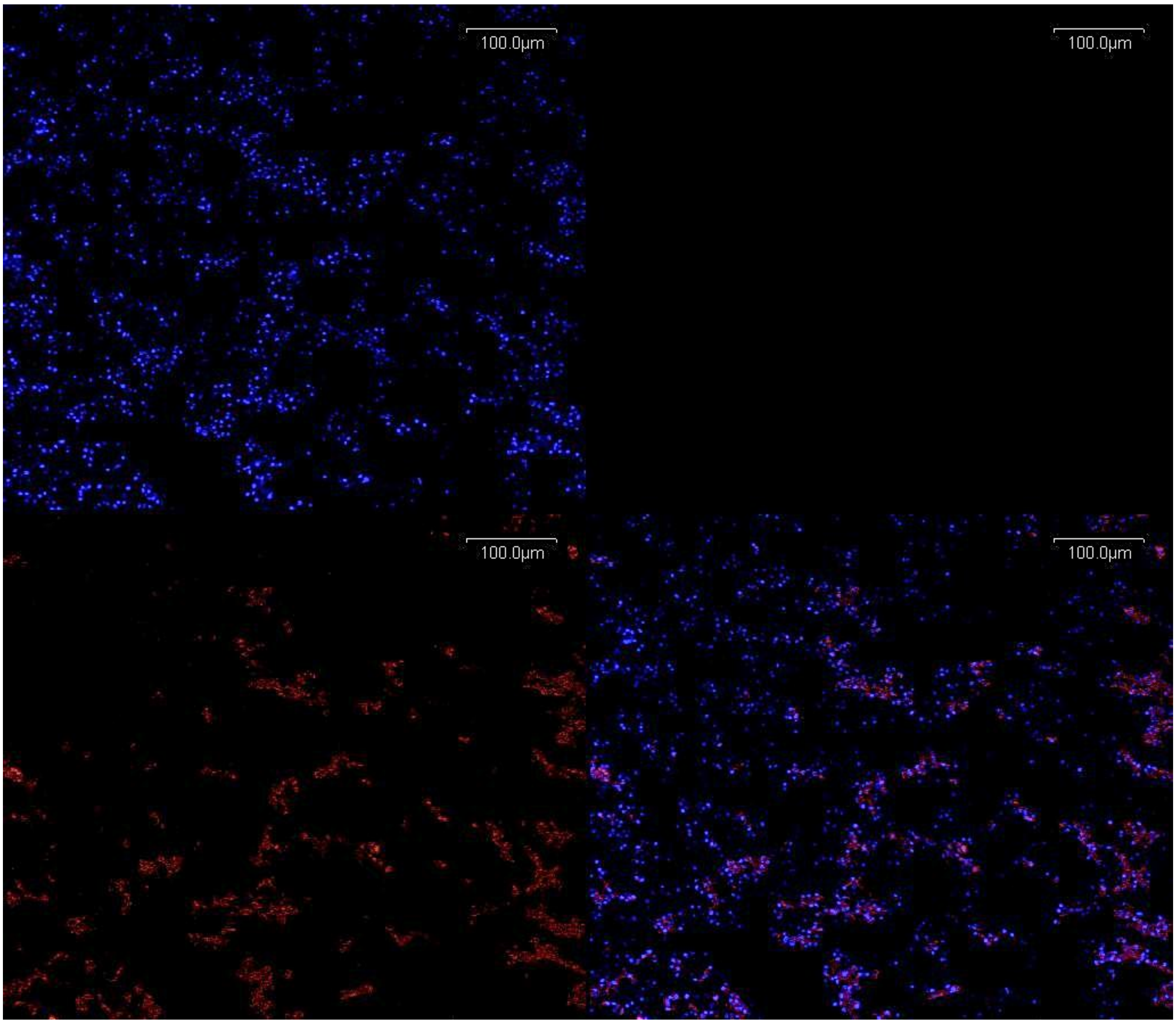

Figure S5. Confocal microscopy image of chicken embryo tissues following administration of Y_2O_3 NPs at concentration 10 μg/ml at day 10 of incubation. Lens magnification 20x. Blue fluorescence corresponds to nuclei stained with Hoechest 33342, while red fluorescence indicates Y_2O_3 NPs.

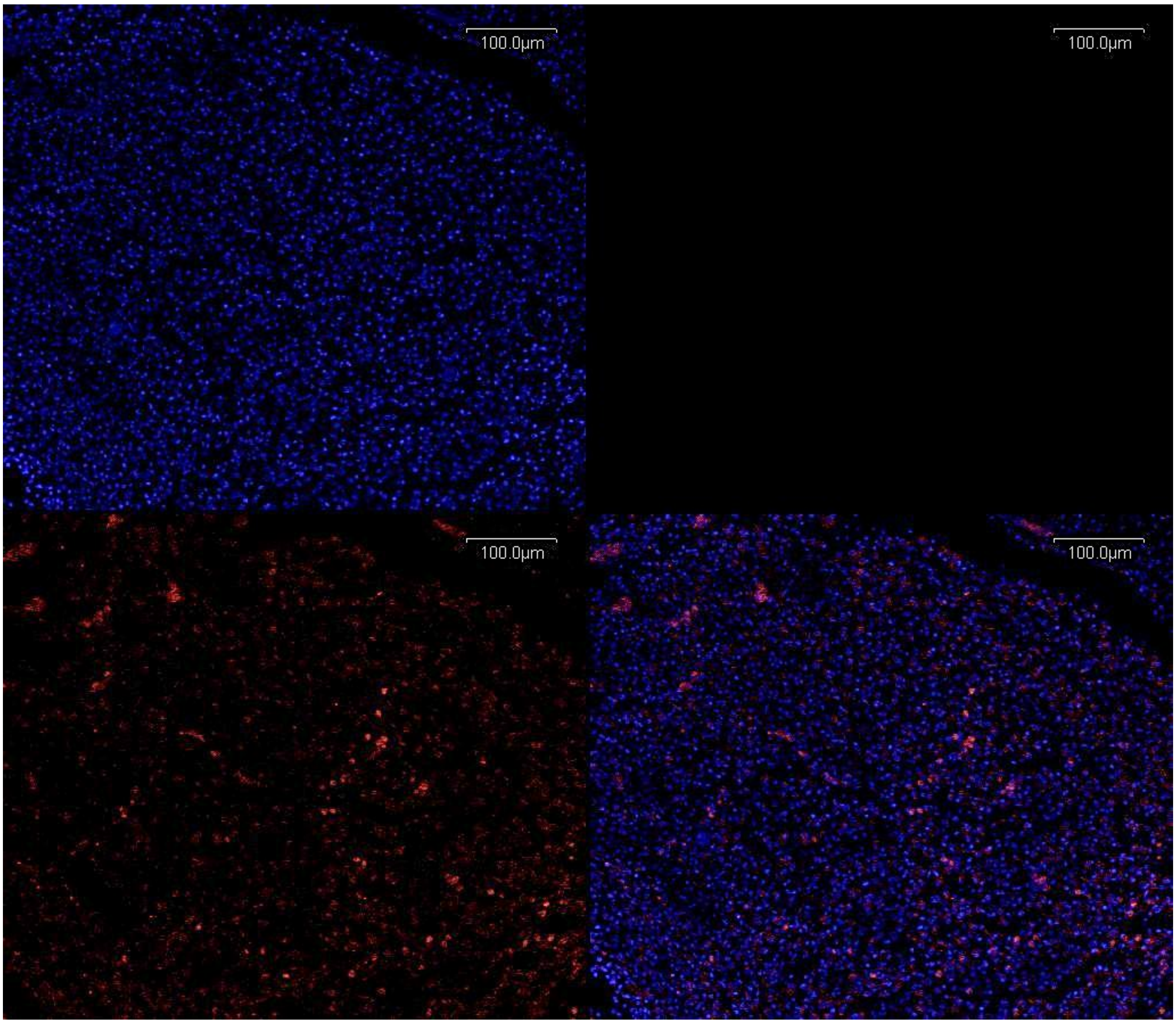

Figure S6. Confocal microscopy image of chicken embryo tissues following administration of Y_2O_3 NPs at concentration 10 μg/ml at day 10 of incubation. Lens magnification 20x. Blue fluorescence corresponds to nuclei stained with Hoechest 33342, while red fluorescence indicates Y_2O_3 NPs.

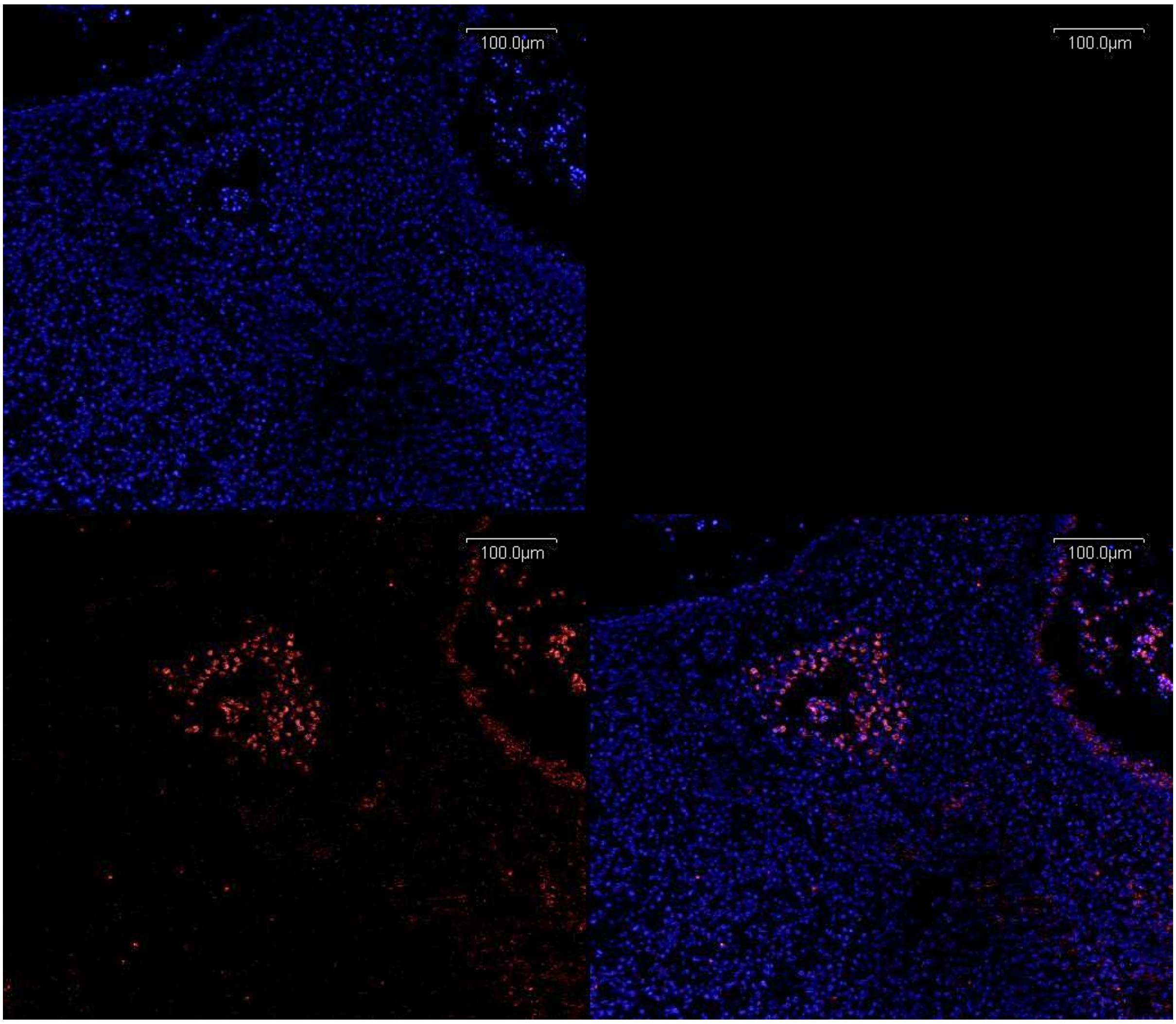

Figure S7. Confocal microscopy image of chicken embryo tissues following administration of Y_2O_3 NPs at concentration 100 µg/ml at day 5 of incubation. Lens magnification 20x. Blue fluorescence corresponds to nuclei stained with Hoechest 33342, while red fluorescence indicates Y_2O_3 NPs.

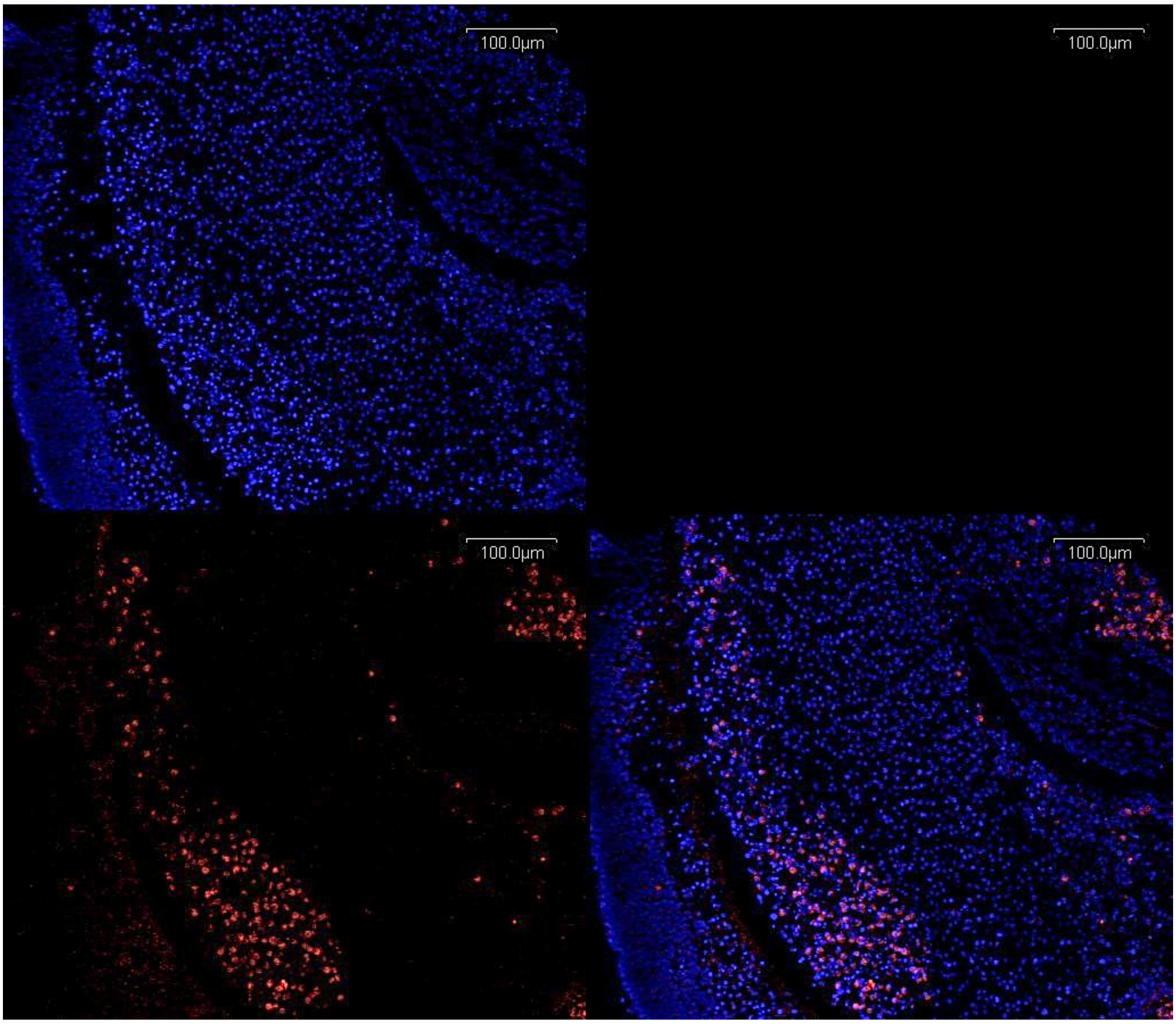

Figure S8. Confocal microscopy image of chicken embryo tissues following administration of Y_2O_3 NPs at concentration 100 μg/ml at day 5 of incubation. Lens magnification 20x. Blue fluorescence corresponds to nuclei stained with Hoechest 33342, while red fluorescence indicates Y_2O_3 NPs.

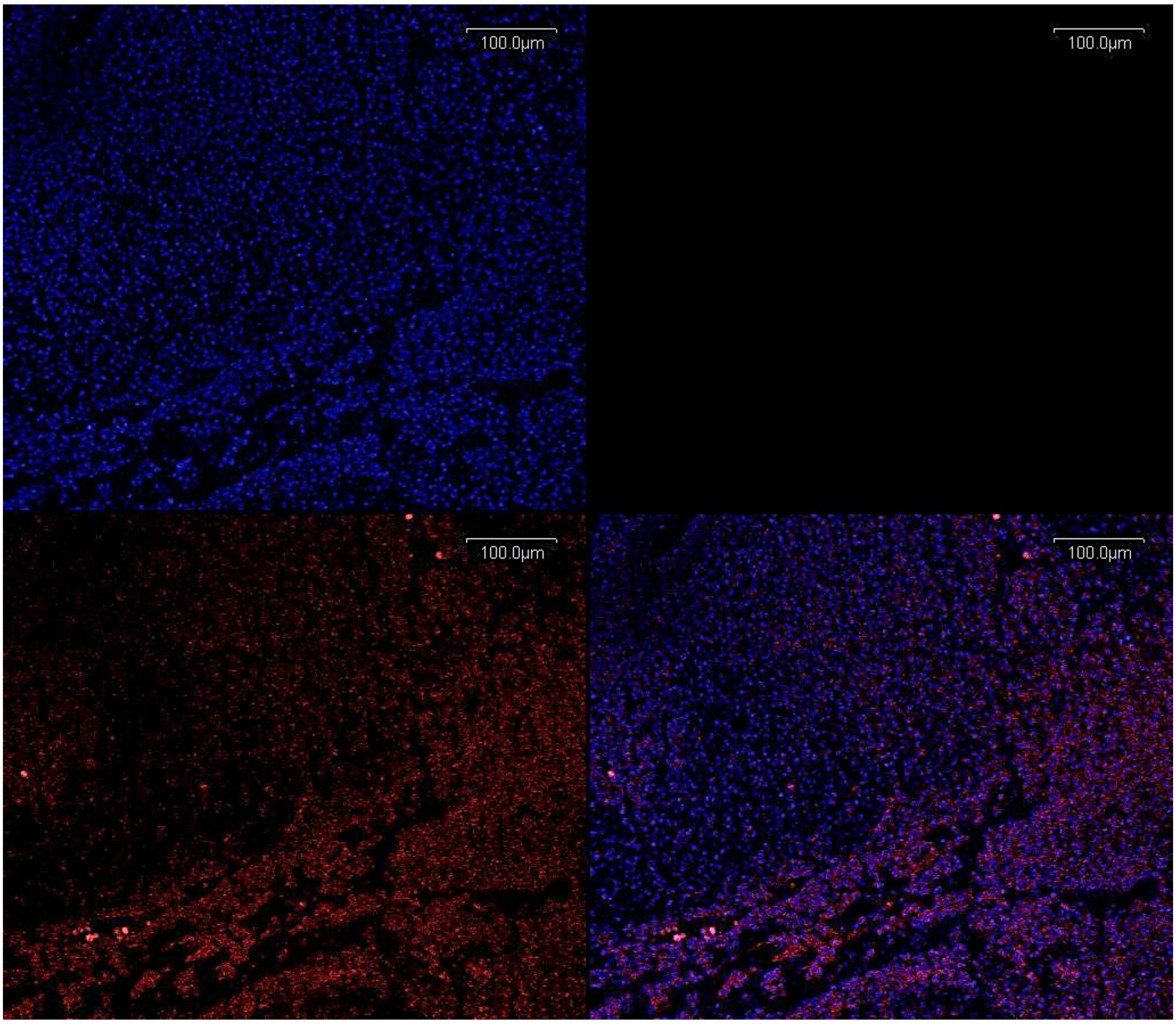

Figure S9. Confocal microscopy image of chicken embryo tissues following administration of Y_2O_3 NPs at concentration 100 µg/ml at day 7 of incubation. Lens magnification 20x. Blue fluorescence corresponds to nuclei stained with Hoechest 33342, while red fluorescence indicates Y_2O_3 NPs.

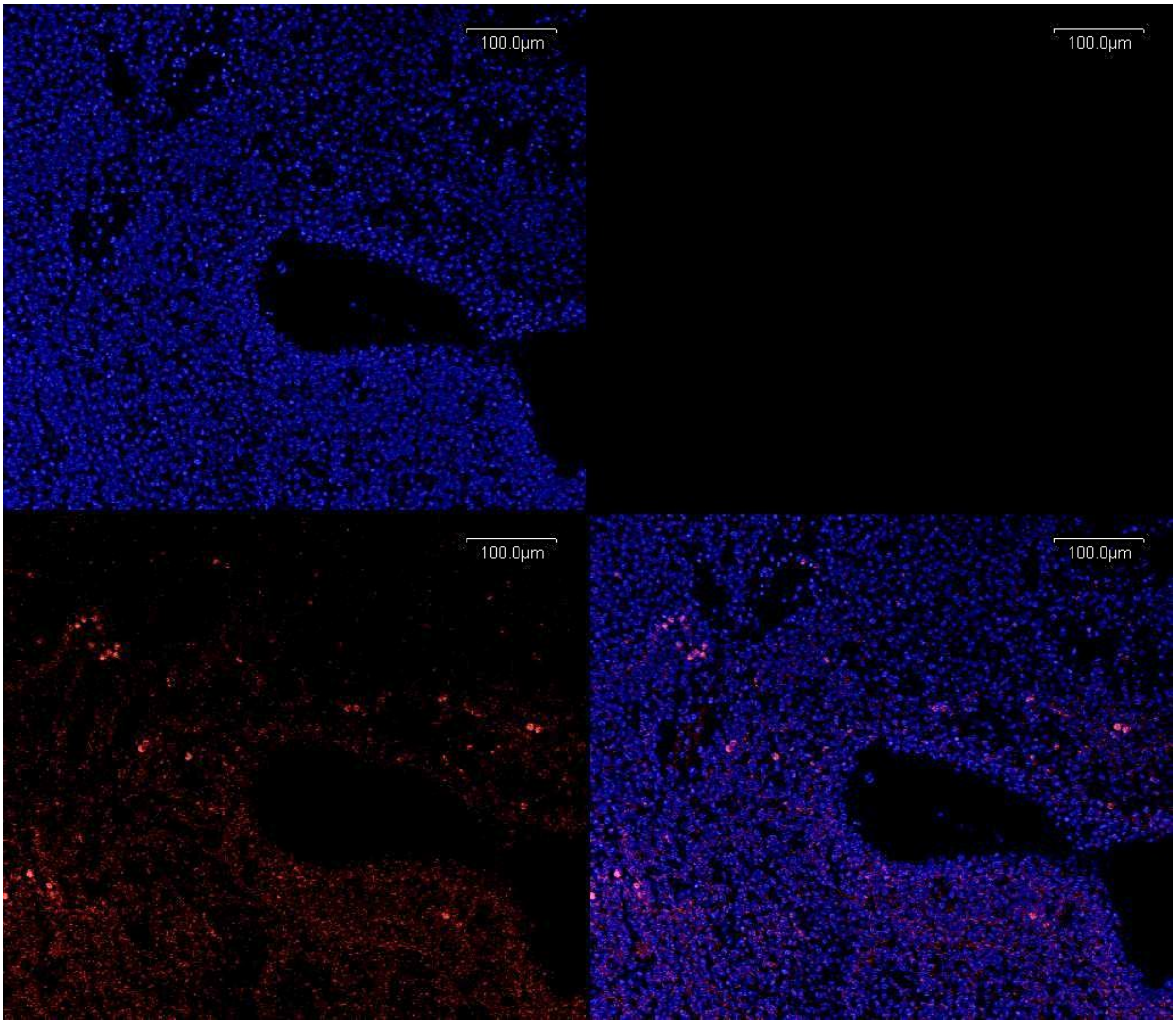

Figure S10. Confocal microscopy image of chicken embryo tissues following administration of Y_2O_3 NPs at concentration 100 µg/ml at day 7 of incubation. Lens magnification 20x. Blue fluorescence corresponds to nuclei stained with Hoechest 33342, while red fluorescence indicates Y_2O_3 NPs.

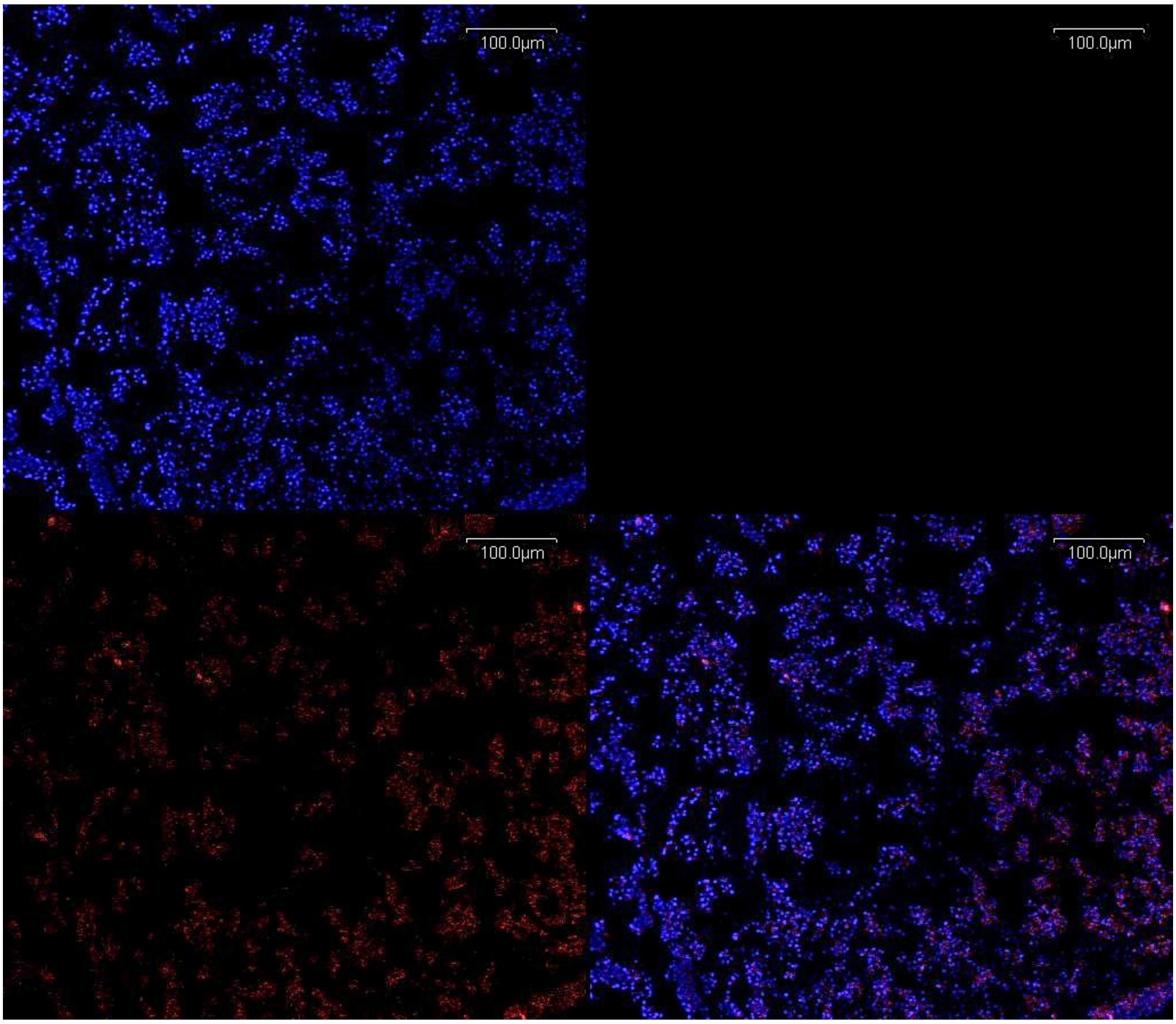

Figure S11. Confocal microscopy image of chicken embryo tissues following administration of Y_2O_3 NPs at concentration 100 μg/ml at day 10 of incubation. Lens magnification 20x. Blue fluorescence corresponds to nuclei stained with Hoechest 33342, while red fluorescence indicates Y_2O_3 NPs.

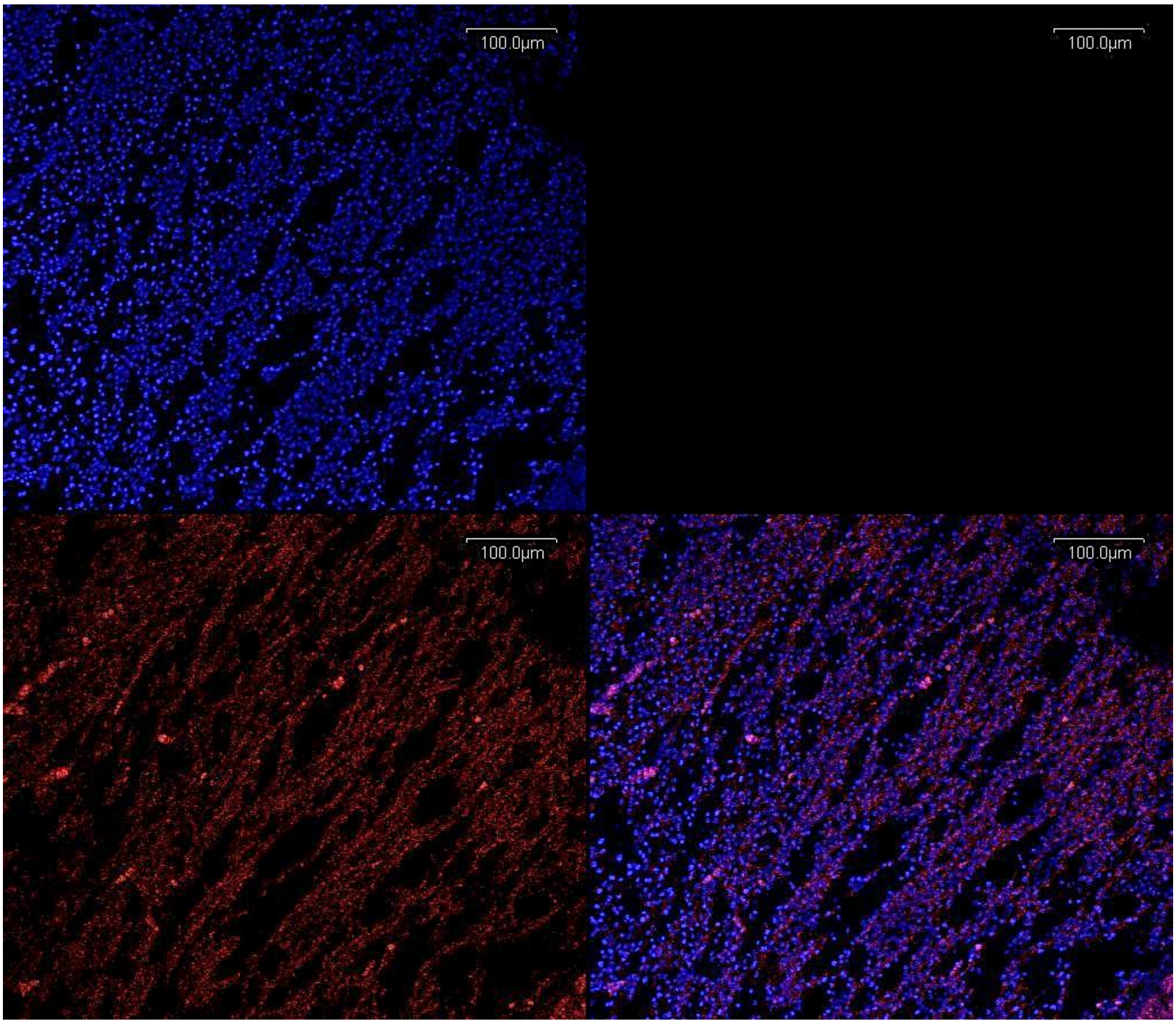

Figure S12. Confocal microscopy image of chicken embryo tissues following administration of Y_2O_3 NPs at concentration 100 µg/ml at day 10 of incubation. Lens magnification 20x. Blue fluorescence corresponds to nuclei stained with Hoechest 33342, while red fluorescence indicates Y_2O_3 NPs.

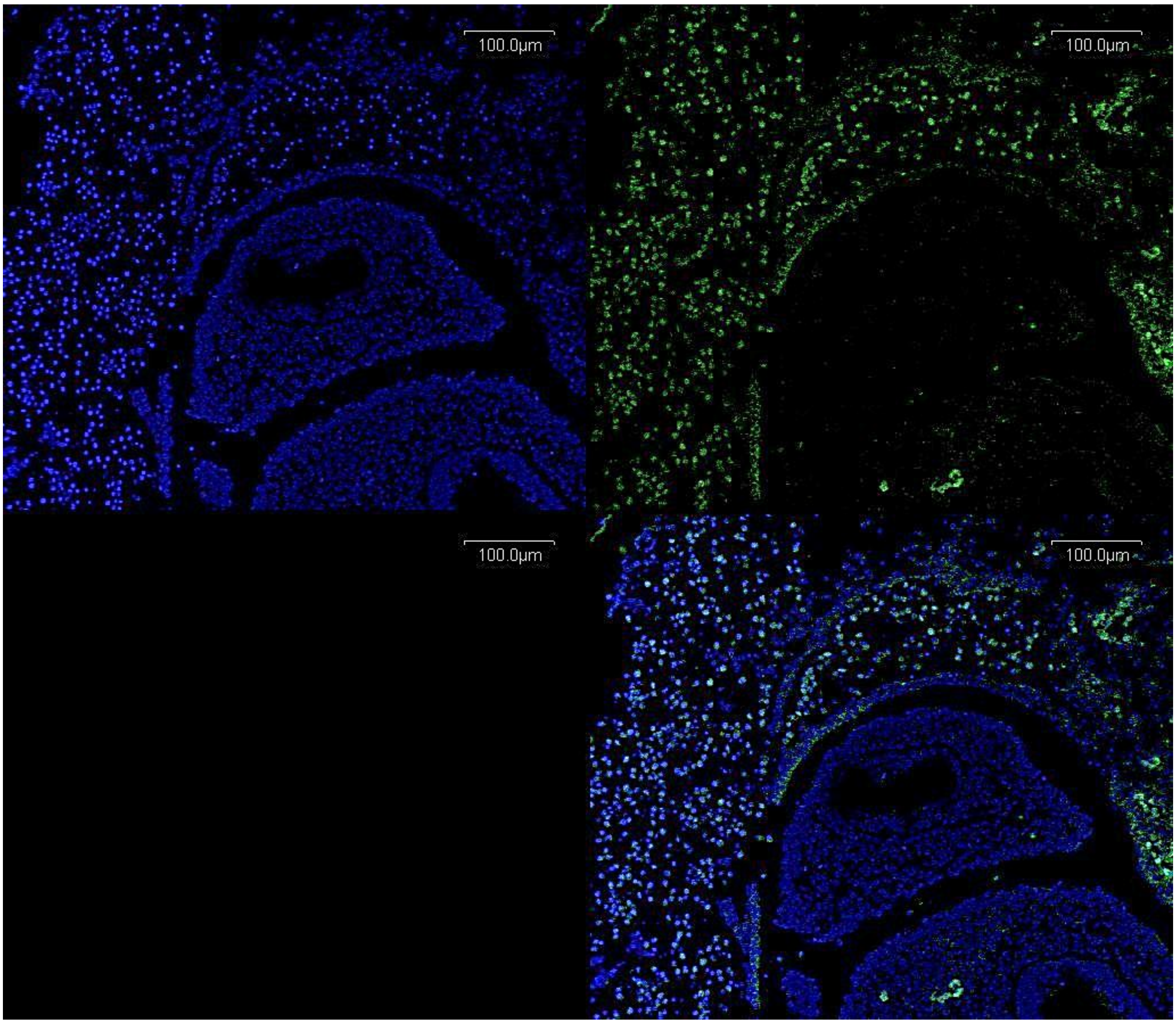

Figure S13. Confocal microscopy image of chicken embryo tissues following administration of ZrO_2 NPs at concentration 10 μg/ml at day 5 of incubation. Lens magnification 20x. Blue fluorescence corresponds to nuclei stained with Hoechest 33342, while green fluorescence indicates ZrO_2 NPs.

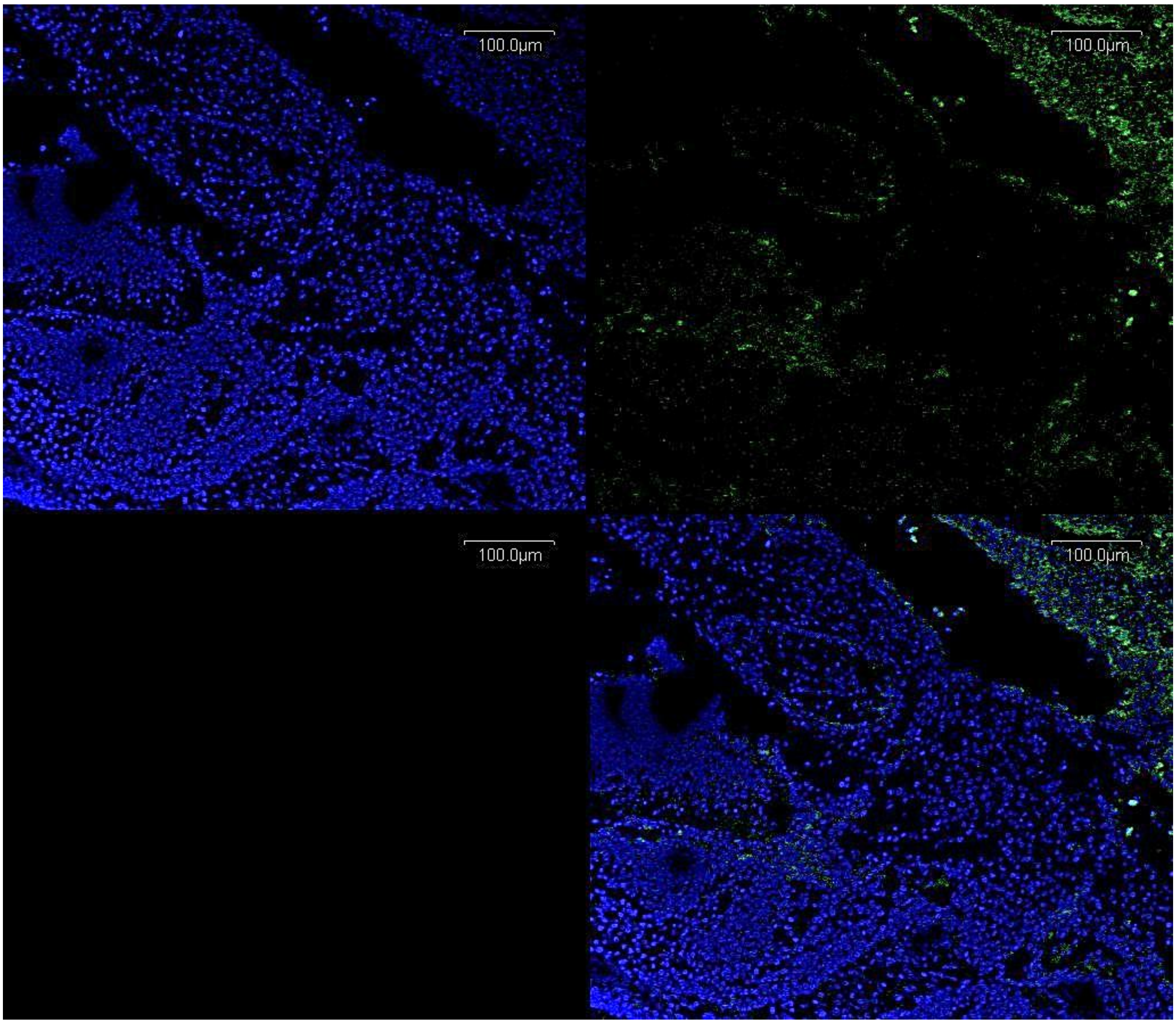

Figure S14. Confocal microscopy image of chicken embryo tissues following administration of ZrO_2 NPs at concentration 10 μg/ml at day 5 of incubation. Lens magnification 20x. Blue fluorescence corresponds to nuclei stained with Hoechest 33342, while green fluorescence indicates ZrO_2 NPs.

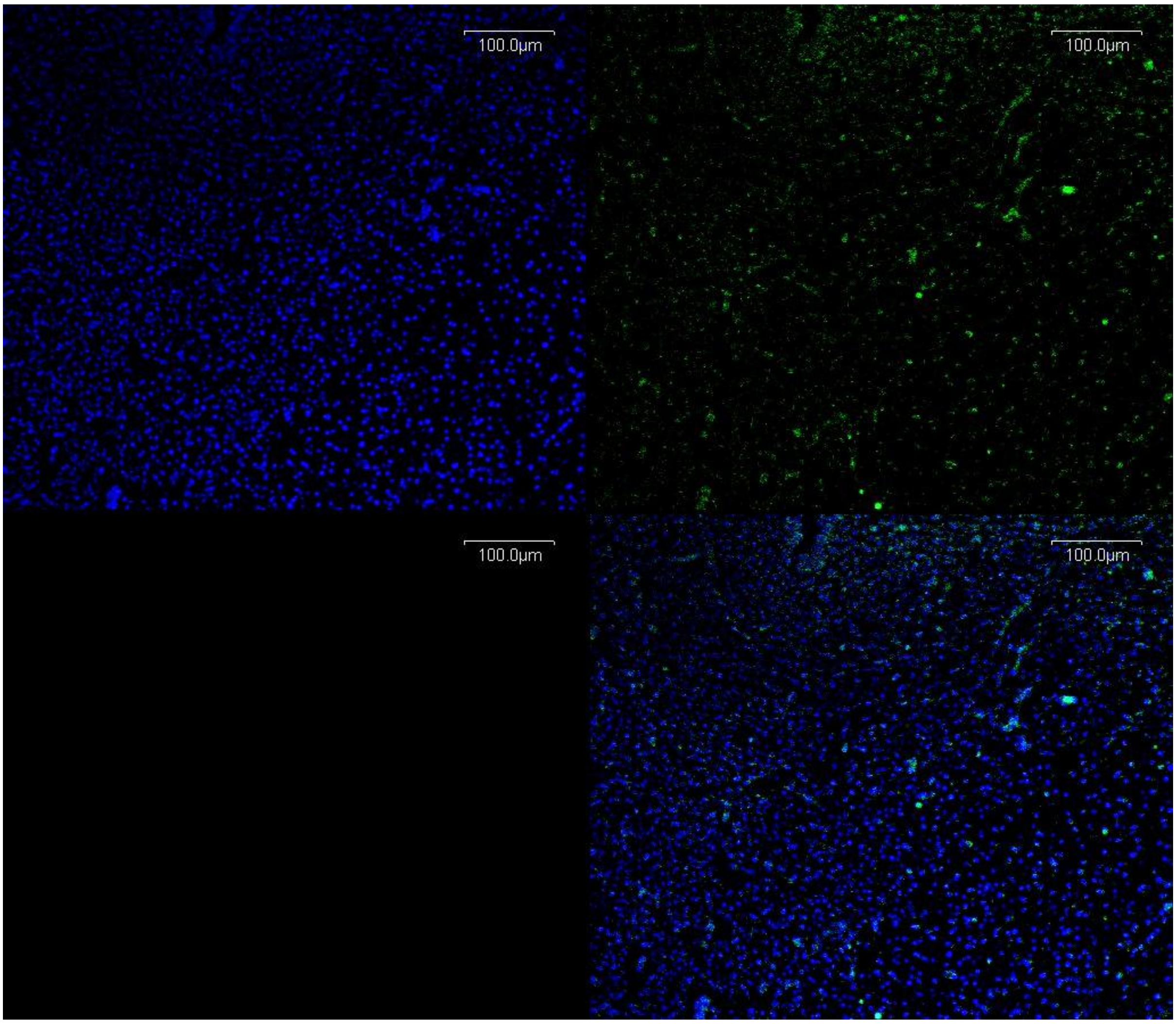

Figure S15. Confocal microscopy image of chicken embryo tissues following administration of ZrO_2 NPs at concentration 10 μg/ml at day 7 of incubation. Lens magnification 20x. Blue fluorescence corresponds to nuclei stained with Hoechest 33342, while green fluorescence indicates ZrO_2 NPs.

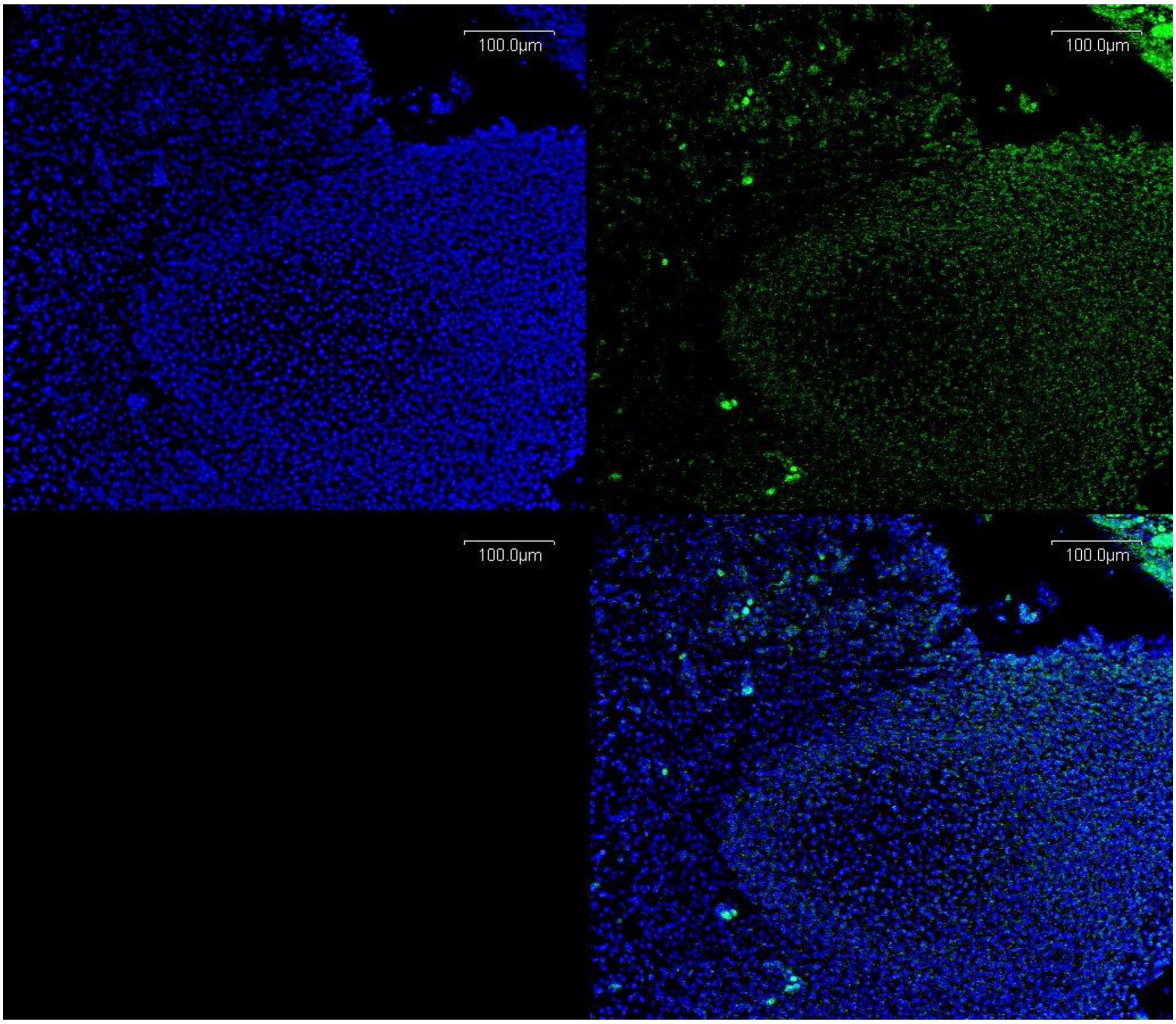

Figure S16. Confocal microscopy image of chicken embryo tissues following administration of ZrO_2 NPs at concentration 10 μg/ml at day 7 of incubation. Lens magnification 20x. Blue fluorescence corresponds to nuclei stained with Hoechest 33342, while green fluorescence indicates ZrO_2 NPs.

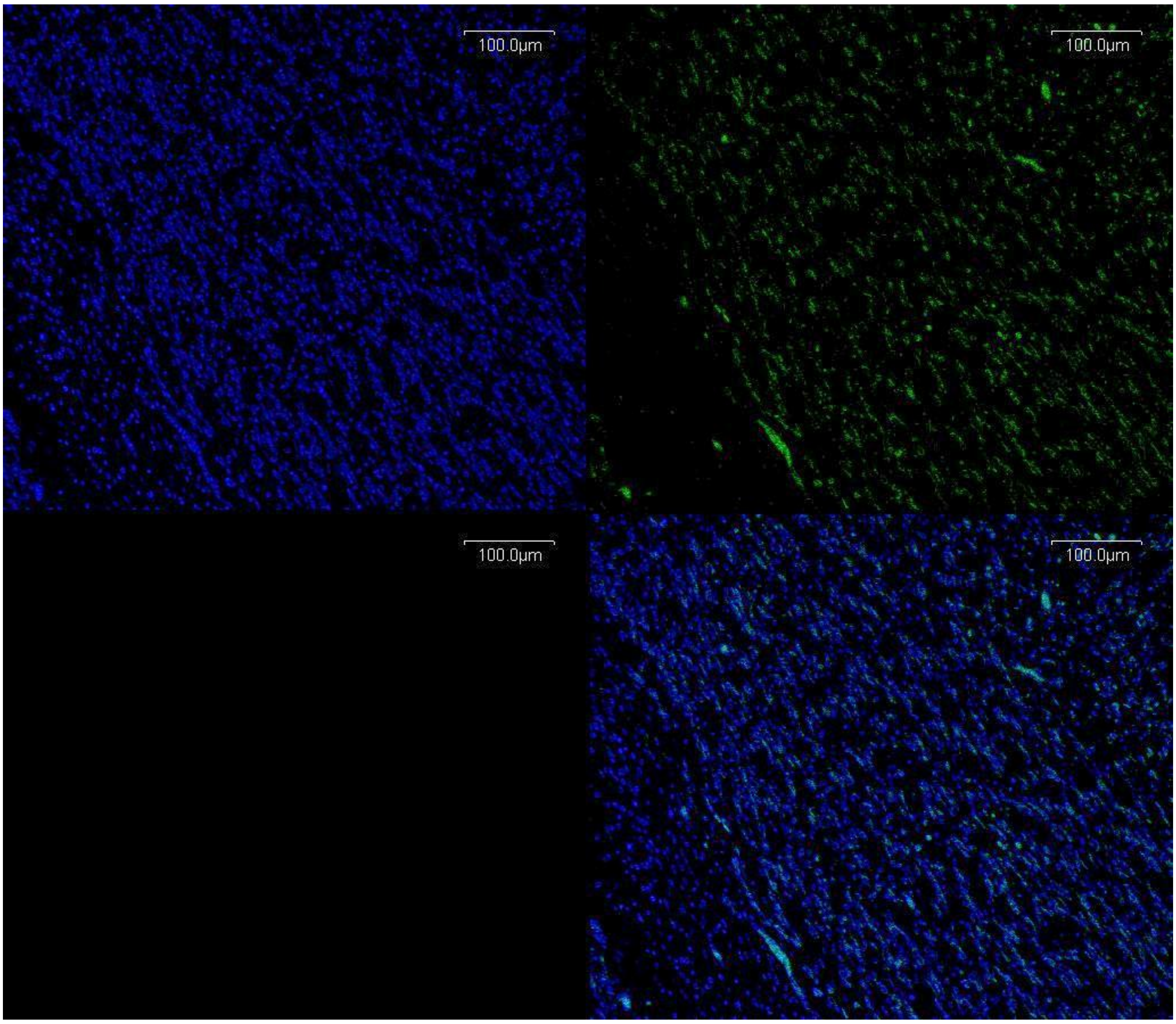

Figure S17. Confocal microscopy image of chicken embryo tissues following administration of ZrO_2 NPs at concentration 10 μg/ml at day 10 of incubation. Lens magnification 20x. Blue fluorescence corresponds to nuclei stained with Hoechest 33342, while green fluorescence indicates ZrO_2 NPs.

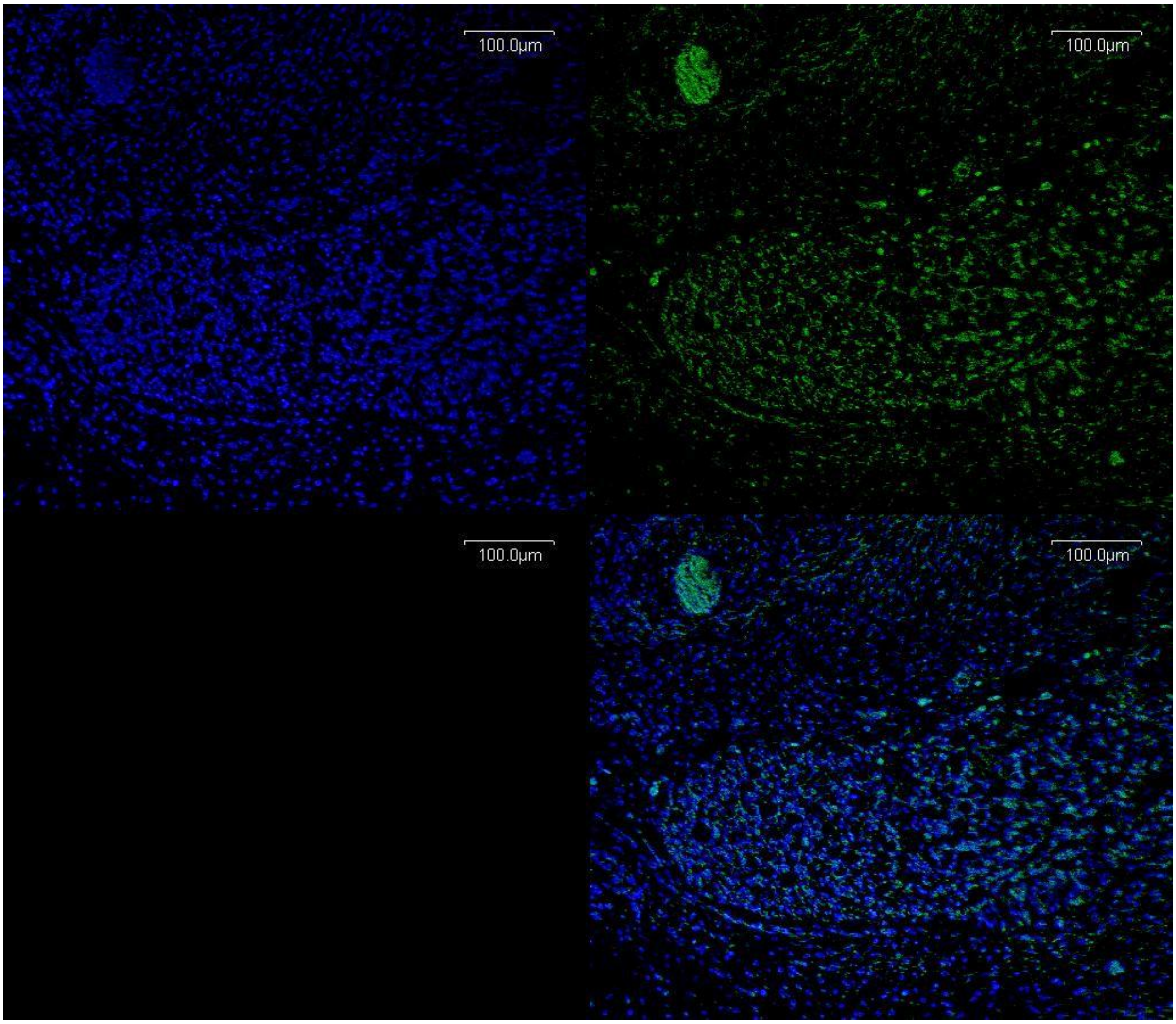

Figure S18. Confocal microscopy image of chicken embryo tissues following administration of ZrO_2 NPs at concentration 10 µg/ml at day 10 of incubation. Lens magnification 20x. Blue fluorescence corresponds to nuclei stained with Hoechest 33342, while green fluorescence indicates ZrO_2 NPs.

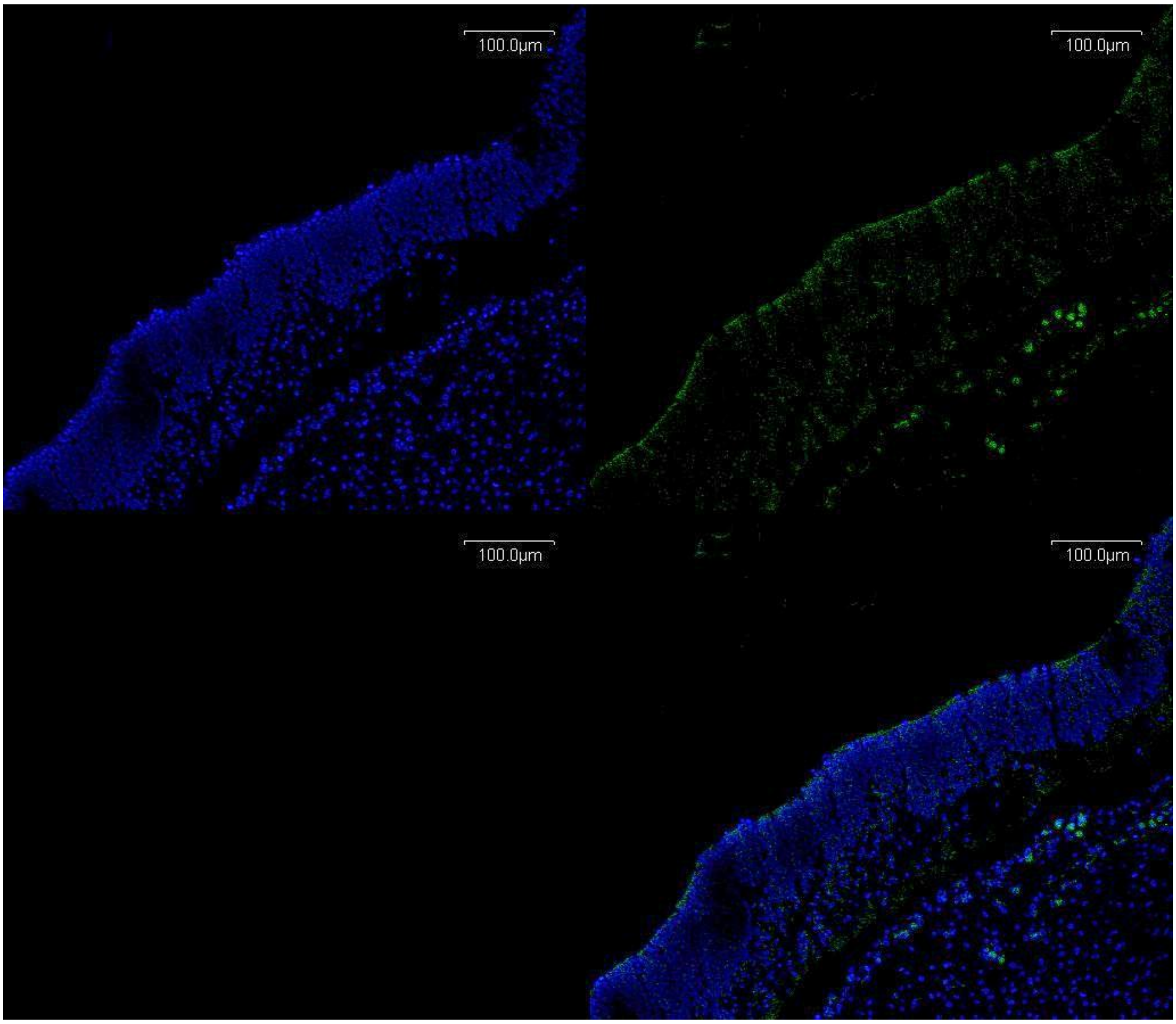

Figure S19. Confocal microscopy image of chicken embryo tissues following administration of ZrO_2 NPs at concentration 100 μg/ml at day 5 of incubation. Lens magnification 20x. Blue fluorescence corresponds to nuclei stained with Hoechest 33342, while green fluorescence indicates ZrO_2 NPs.

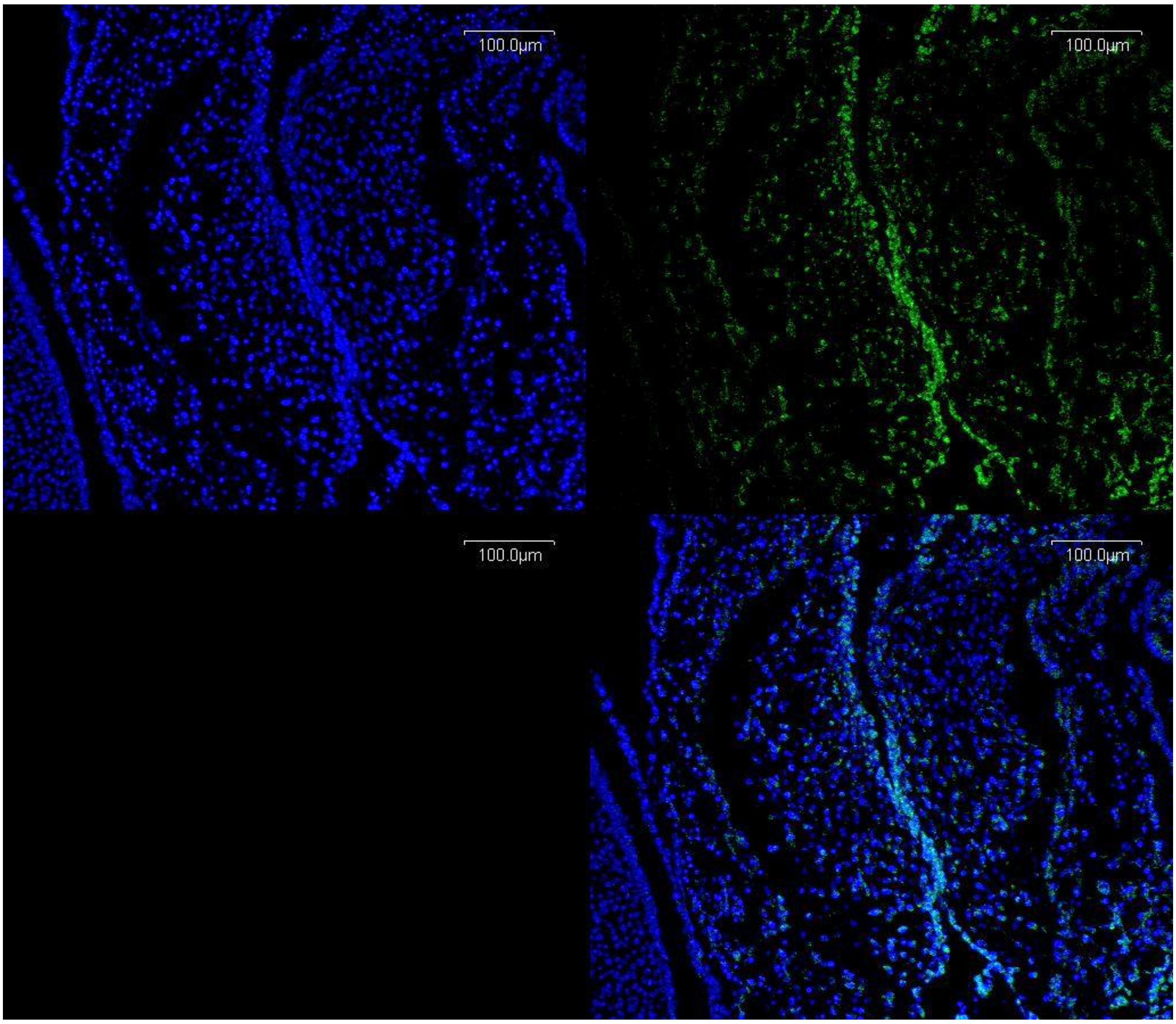

Figure S20. Confocal microscopy image of chicken embryo tissues following administration of ZrO_2 NPs at concentration 100 μg/ml at day 5 of incubation. Lens magnification 20x. Blue fluorescence corresponds to nuclei stained with Hoechest 33342, while green fluorescence indicates ZrO_2 NPs.

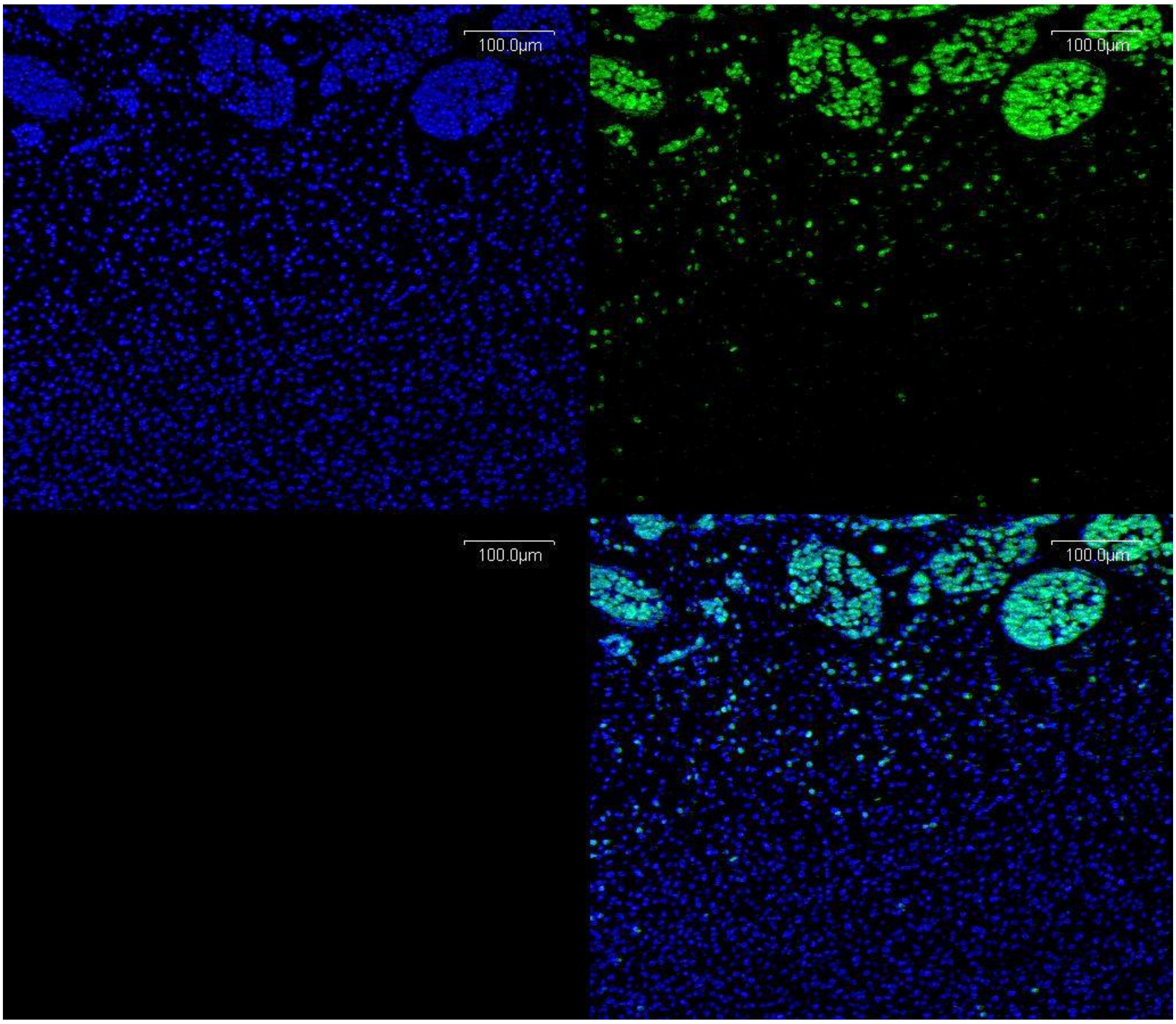

Figure S21. Confocal microscopy image of chicken embryo tissues following administration of ZrO_2 NPs at concentration 100 µg/ml at day 7 of incubation. Lens magnification 20x. Blue fluorescence corresponds to nuclei stained with Hoechest 33342, while green fluorescence indicates ZrO_2 NPs.

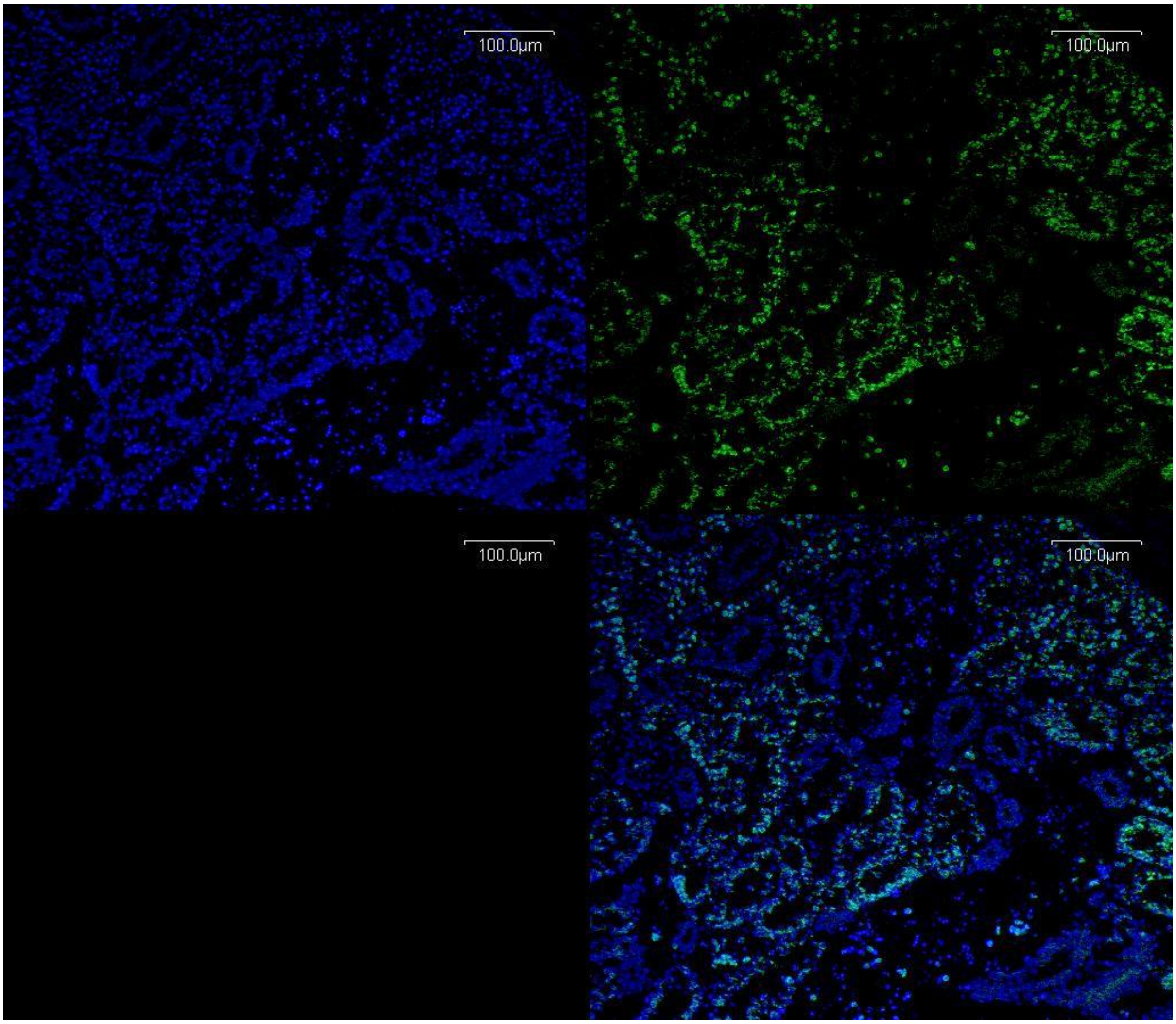

Figure S22. Confocal microscopy image of chicken embryo tissues following administration of ZrO_2 NPs at concentration 100 μg/ml at day 7 of incubation. Lens magnification 20x. Blue fluorescence corresponds to nuclei stained with Hoechest 33342, while green fluorescence indicates ZrO_2 NPs.

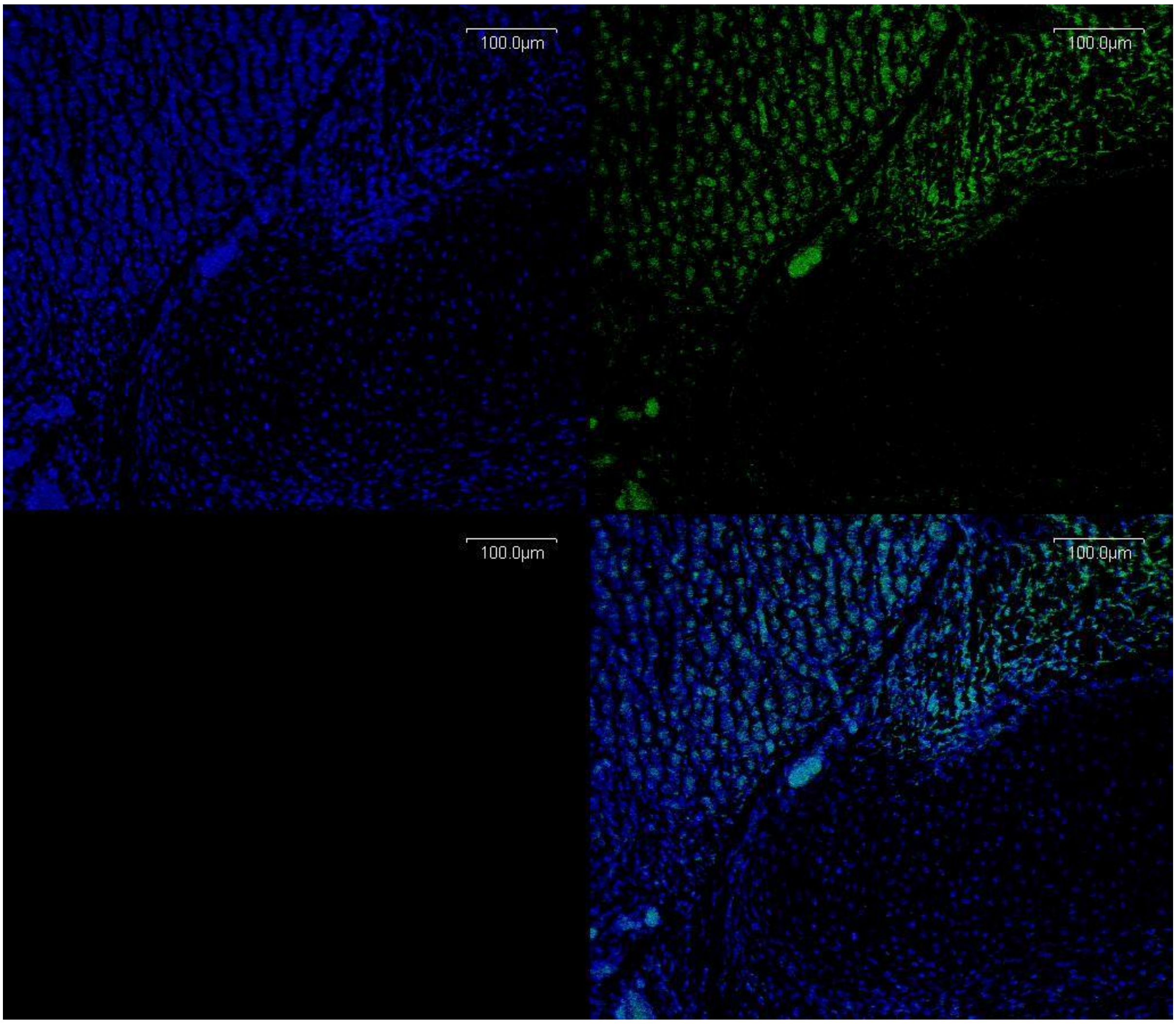

Figure S23. Confocal microscopy image of chicken embryo tissues following administration of ZrO_2 NPs at concentration 100 µg/ml at day 10 of incubation. Lens magnification 20x. Blue fluorescence corresponds to nuclei stained with Hoechest 33342, while green fluorescence indicates ZrO_2 NPs.

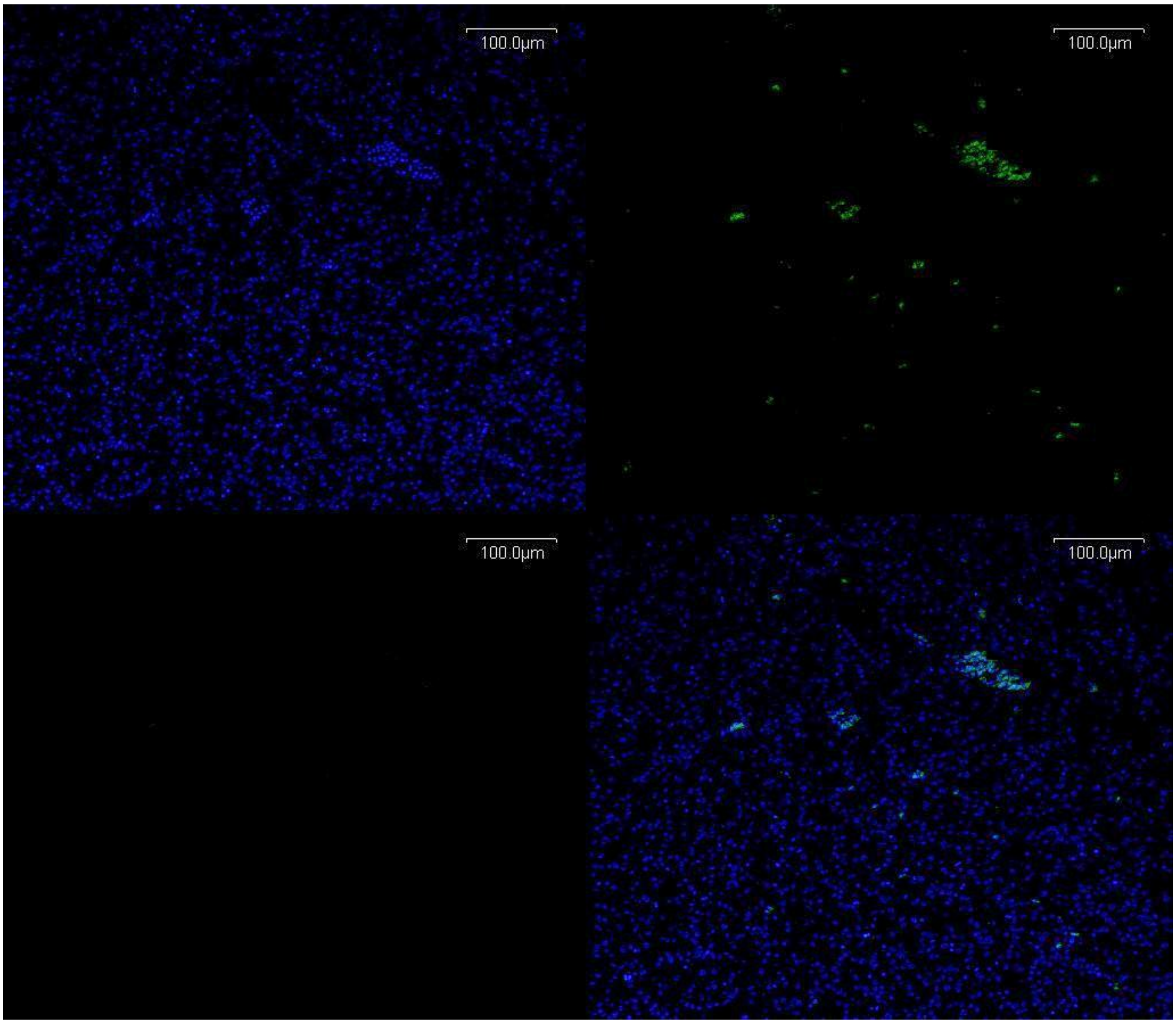

Figure S24. Confocal microscopy image of chicken embryo tissues following administration of ZrO_2 NPs at concentration 100 µg/ml at day 10 of incubation. Lens magnification 20x. Blue fluorescence corresponds to nuclei stained with Hoechest 33342, while green fluorescence indicates ZrO_2 NPs.
